# Supplementary figures and images for: Silencing NRF2 enhances arsenic trioxide-induced ferroptosis in hepatocellular carcinoma cells
Source: PLoS One. 2025 May 22;20(5):e0322746. doi: 10.1371/journal.pone.0322746 (PMC12097587; doi:10.1371/journal.pone.0322746)

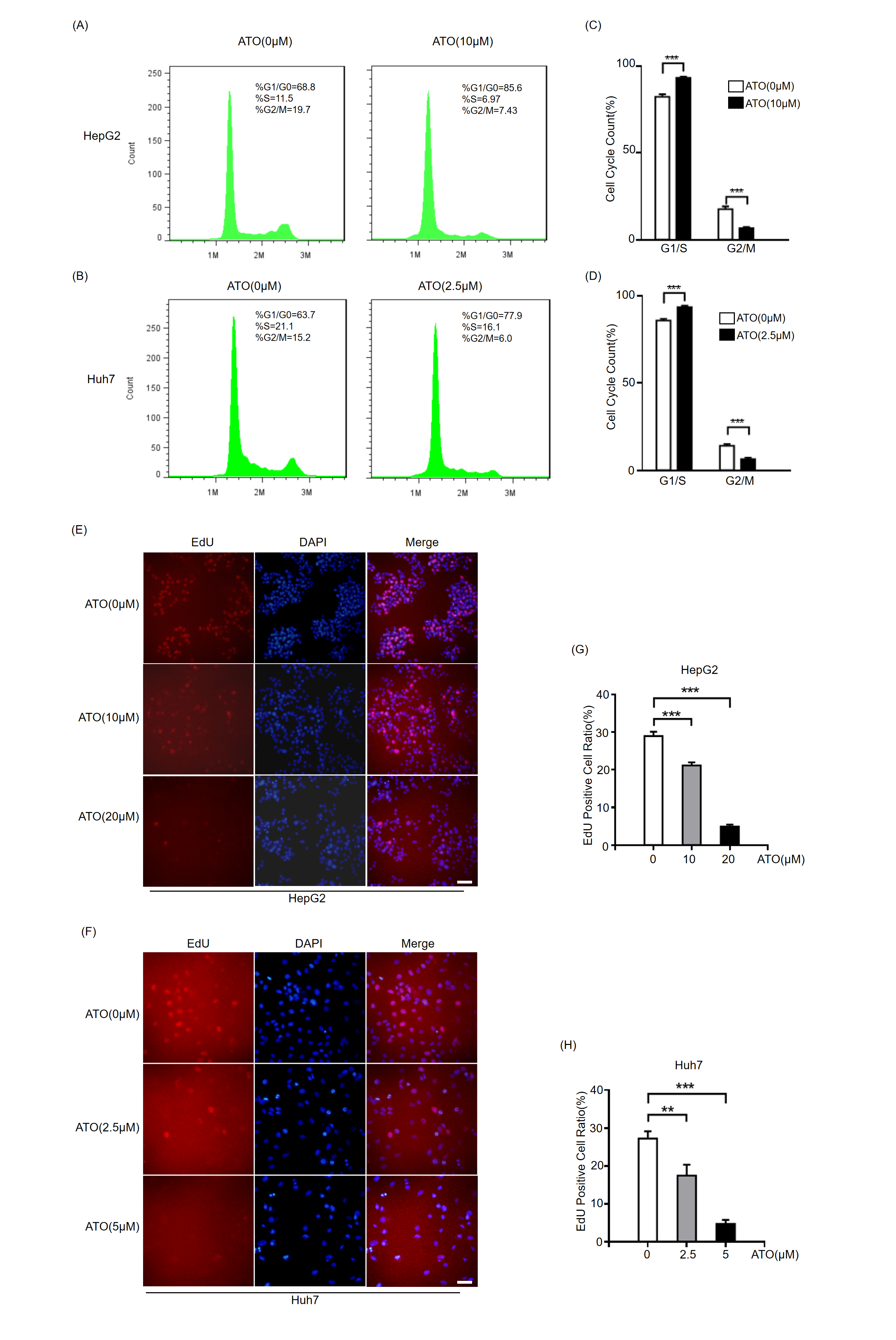

Supplement: S1 Fig — After ATO treatment for 24 hours, HepG2(A) and Huh7(B) cell cycle were analyzed by flow cytometry. Statistical analysis of the cell cycle count in HepG2(C) and Huh7(D). HepG2(E) and Huh7(F) were treated with indicated concentration ATO for 24 hours, then used for EdU cell proliferation assays,which measure the incorporation of EdU into newly synthesized DNA. The nuclei were stained with DAPI. Bar indicates 50μm. Statistical analysis of relative EdU-positive HepG2(G) and Huh7(H) cells. Error bars indicate SD. **,p < 0.01,***,p < 0.001. n = 3. (TIFF) [file pone.0322746.s001.tif]

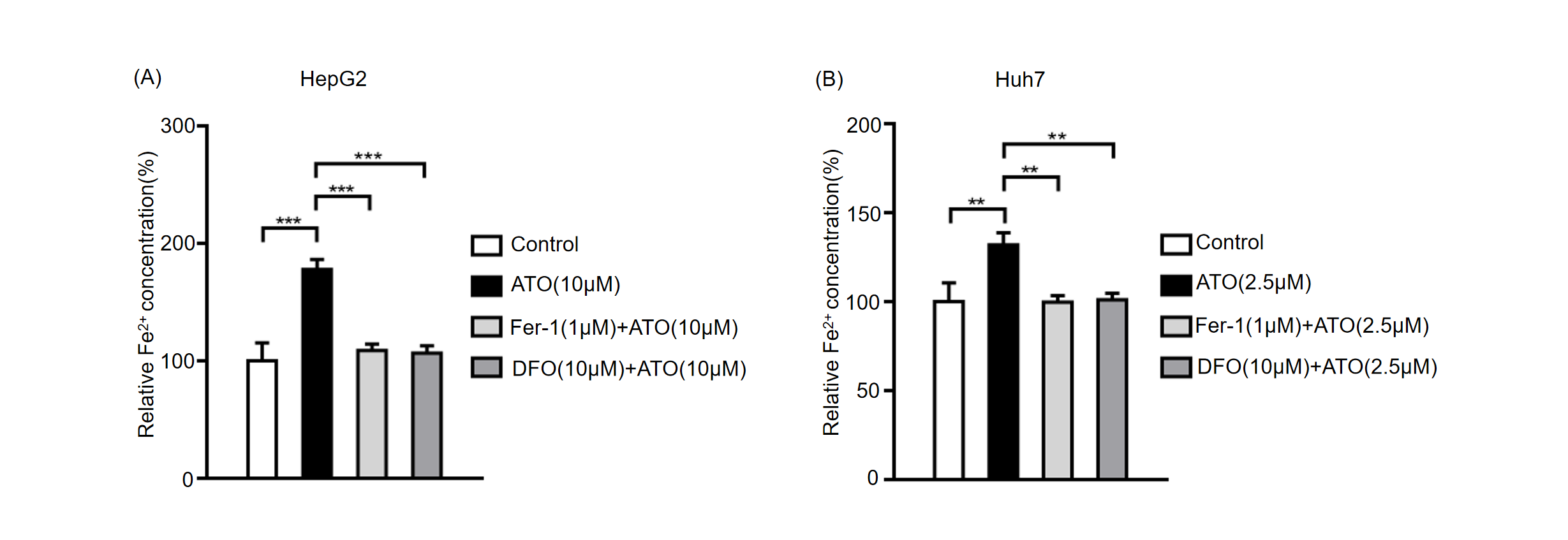

Supplement: S2 Fig — HepG2(A) and Huh7(B) cells were treated with the indicated conditions for 24h, then harvested to analyze the Fe2+ concentration. (TIFF) [file pone.0322746.s002.tif]

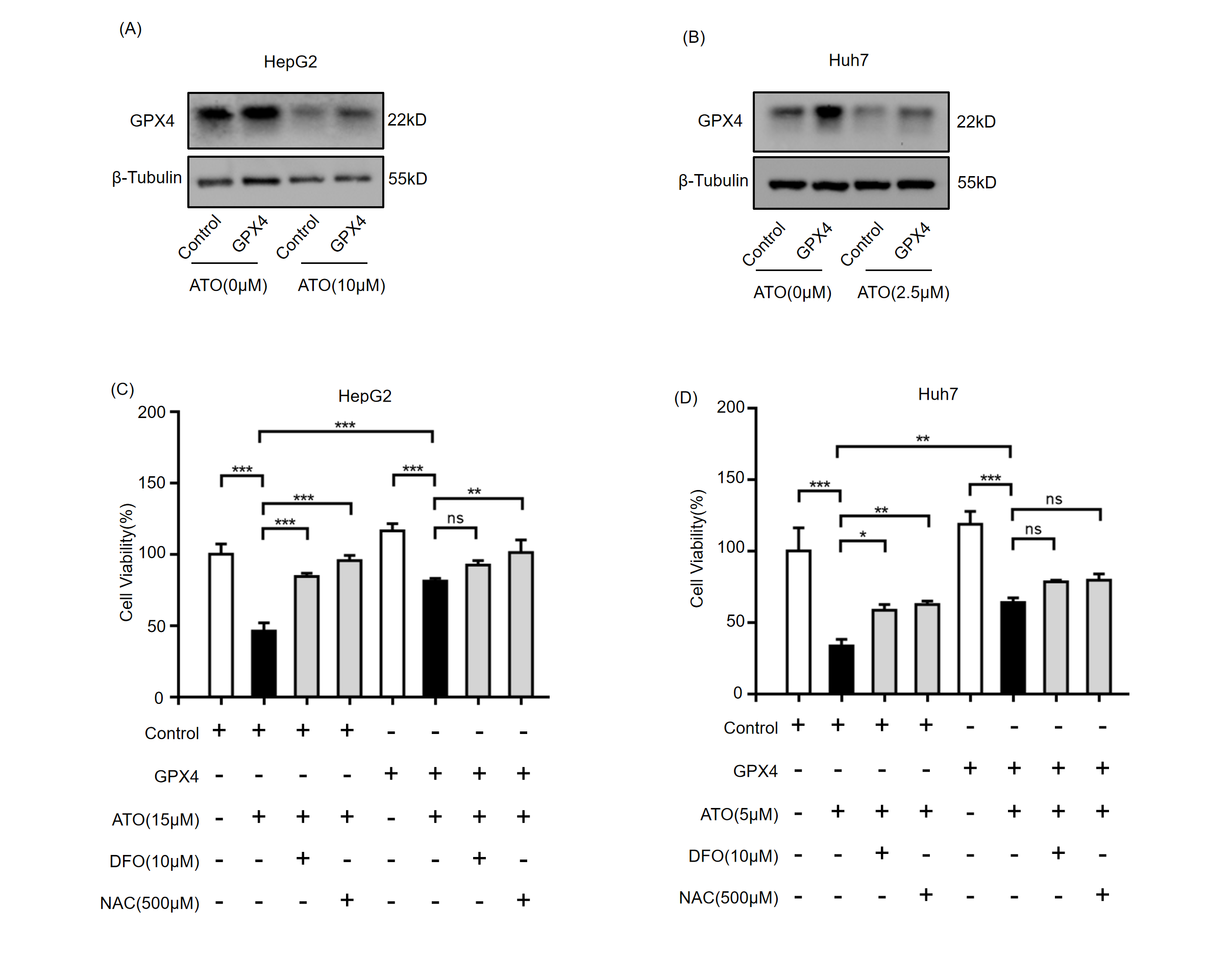

Supplement: S3 Fig — HepG2(A) and Huh7(B) were transfected with pcDNA3.1(-)-GPX4 or control vectors for 24h, then treated with the indicated concentration ATO 24h, then harvested to western blot. HepG2(C) and Huh7(D) were transfected with pcDNA3.1(-)-GPX4 or control vectors for 24h, then treated with the indicated condition for 24h, then harvested to CCK-8 assay. Error bars indicate SD. *,p < 0.05, **,p < 0.01,***,p < 0.001. n = 3. (TIFF) [file pone.0322746.s003.tif]

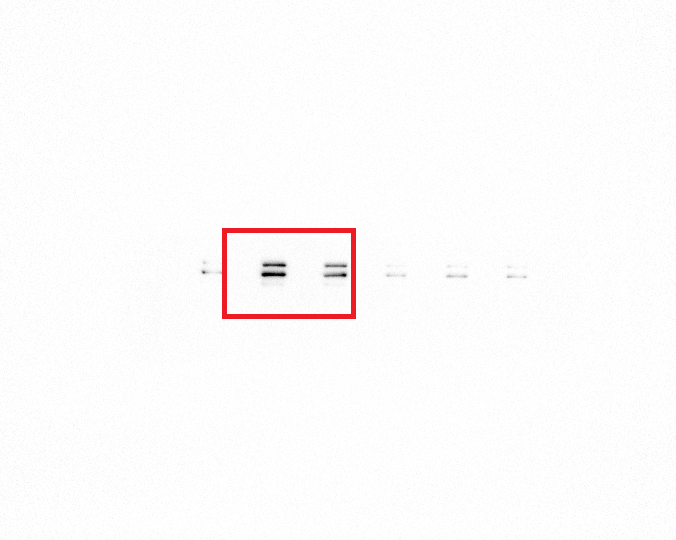

Supplement: S1 Raw images — (ZIP) [file pone.0322746.s004.zip › S1_RAW_images/Fig 4 raw data/Fig 4 c LaminA lane 2-5.tif]

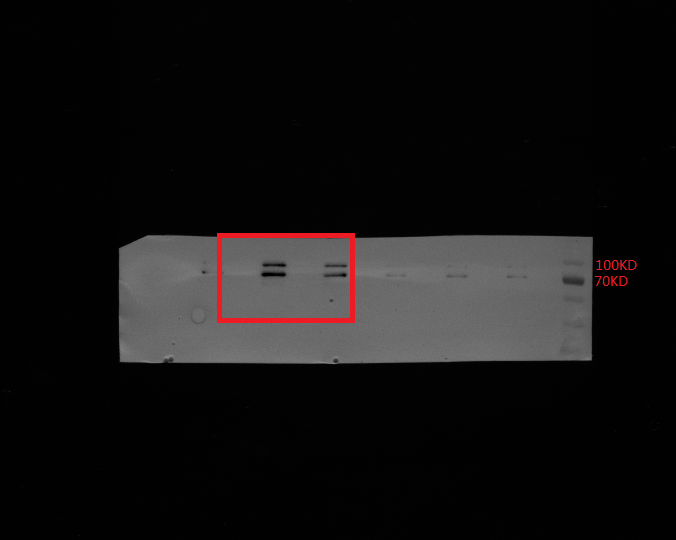

Supplement: S1 Raw images — (ZIP) [file pone.0322746.s004.zip › S1_RAW_images/Fig 4 raw data/Fig 4 c LaminA lane 2-5B.tif]

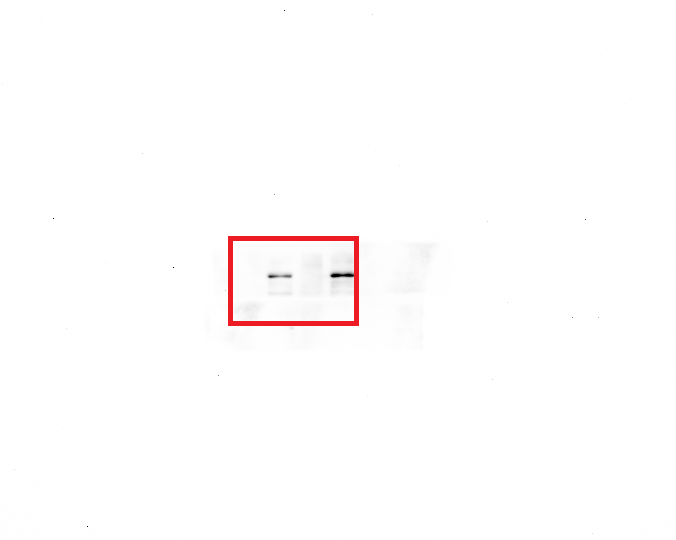

Supplement: S1 Raw images — (ZIP) [file pone.0322746.s004.zip › S1_RAW_images/Fig 4 raw data/Fig 4 c NRF2 lane 2-5.tif]

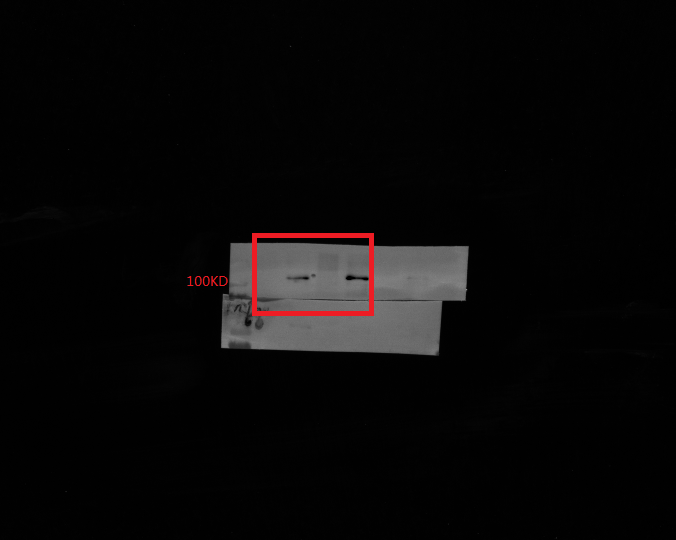

Supplement: S1 Raw images — (ZIP) [file pone.0322746.s004.zip › S1_RAW_images/Fig 4 raw data/Fig 4 c NRF2 lane 2-5B.tif]

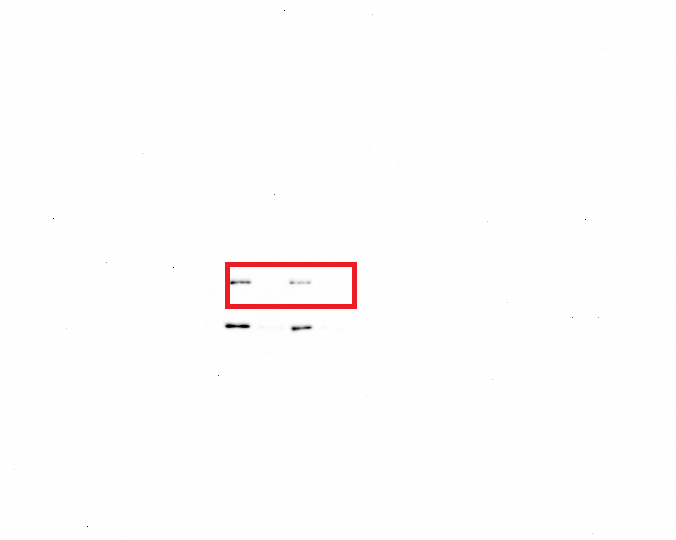

Supplement: S1 Raw images — (ZIP) [file pone.0322746.s004.zip › S1_RAW_images/Fig 4 raw data/Fig 4 c β-Tubulin lane 2-5.tif]

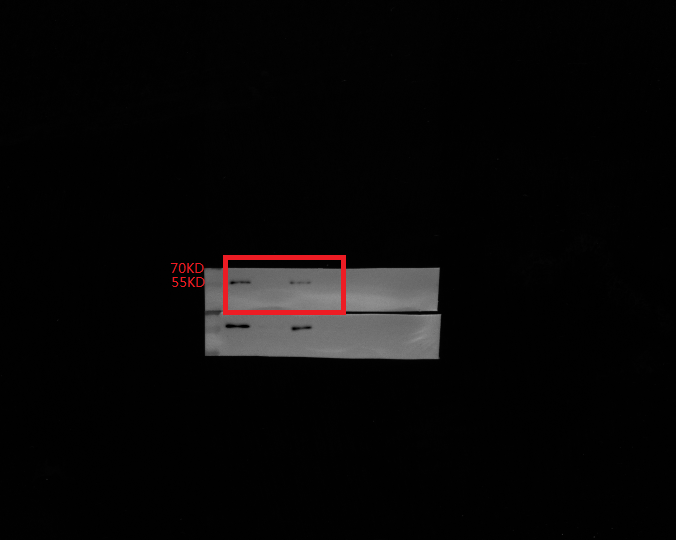

Supplement: S1 Raw images — (ZIP) [file pone.0322746.s004.zip › S1_RAW_images/Fig 4 raw data/Fig 4 c β-Tubulin lane 2-5B.tif]

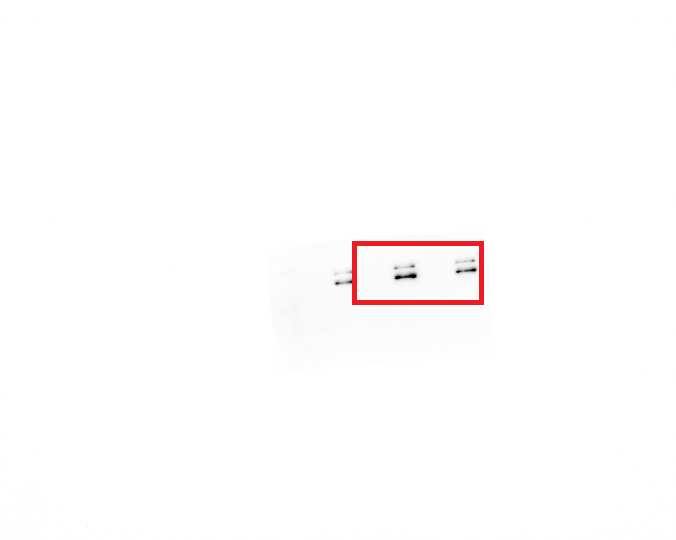

Supplement: S1 Raw images — (ZIP) [file pone.0322746.s004.zip › S1_RAW_images/Fig 4 raw data/Fig 4 d LaminA lane 4-7.tif]

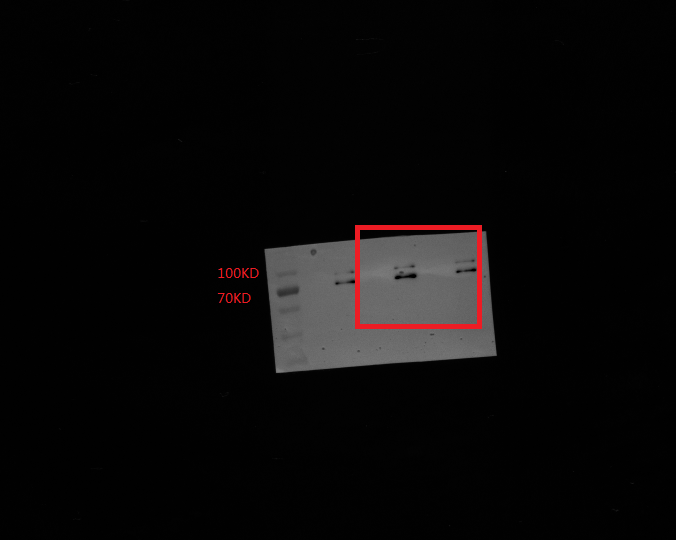

Supplement: S1 Raw images — (ZIP) [file pone.0322746.s004.zip › S1_RAW_images/Fig 4 raw data/Fig 4 d LaminA lane 4-7B.tif]

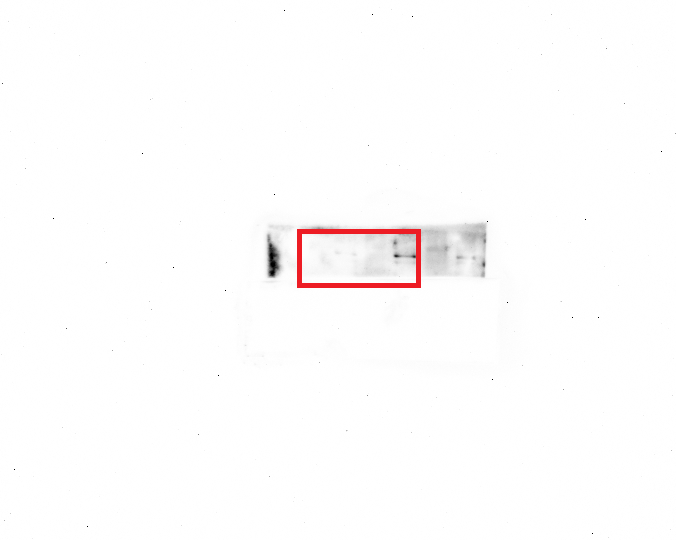

Supplement: S1 Raw images — (ZIP) [file pone.0322746.s004.zip › S1_RAW_images/Fig 4 raw data/Fig 4 d NRF2 lane 2-5.tif]

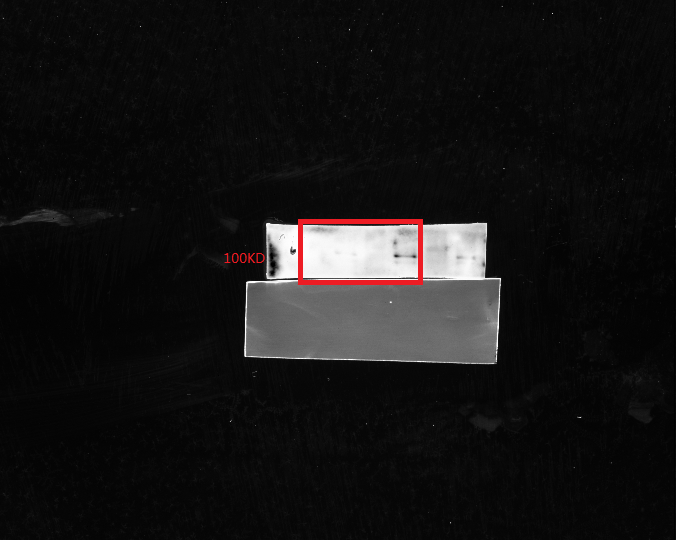

Supplement: S1 Raw images — (ZIP) [file pone.0322746.s004.zip › S1_RAW_images/Fig 4 raw data/Fig 4 d NRF2 lane 2-5B.tif]

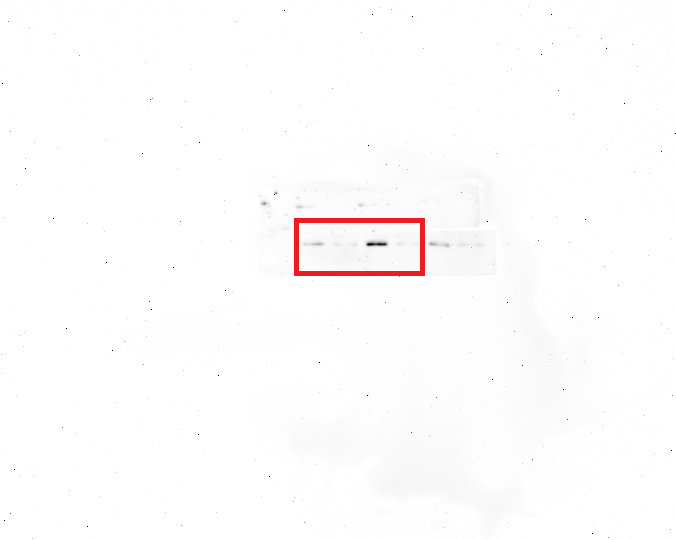

Supplement: S1 Raw images — (ZIP) [file pone.0322746.s004.zip › S1_RAW_images/Fig 4 raw data/Fig 4 d β-Tubulin lane 2-5.tif]

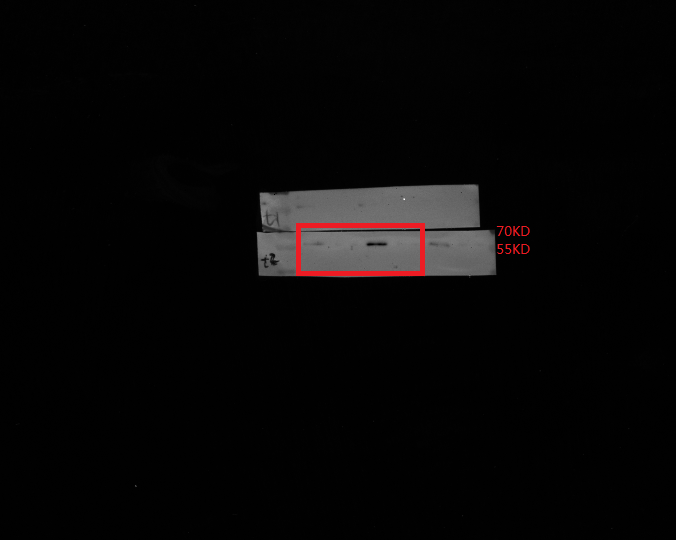

Supplement: S1 Raw images — (ZIP) [file pone.0322746.s004.zip › S1_RAW_images/Fig 4 raw data/Fig 4 d β-Tubulin lane 2-5B.tif]

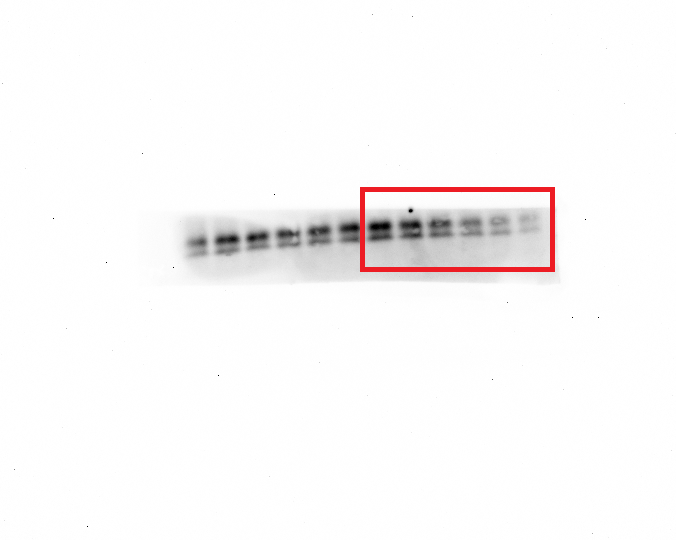

Supplement: S1 Raw images — (ZIP) [file pone.0322746.s004.zip › S1_RAW_images/Fig 4 raw data/Fig 4 e GPX4 lane 8-13.tif]

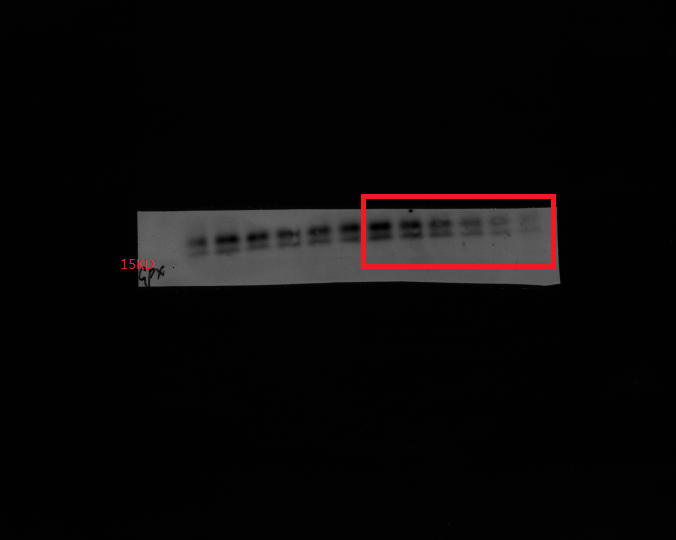

Supplement: S1 Raw images — (ZIP) [file pone.0322746.s004.zip › S1_RAW_images/Fig 4 raw data/Fig 4 e GPX4 lane 8-13B.tif]

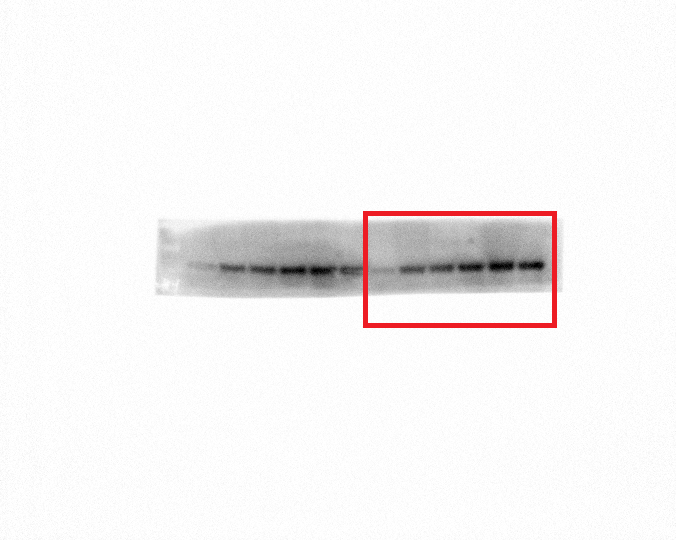

Supplement: S1 Raw images — (ZIP) [file pone.0322746.s004.zip › S1_RAW_images/Fig 4 raw data/Fig 4 e HO-1 lane 8-13.tif]

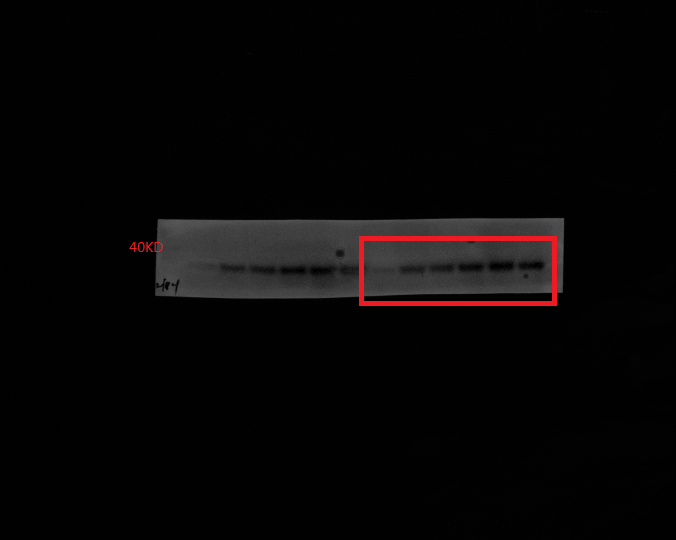

Supplement: S1 Raw images — (ZIP) [file pone.0322746.s004.zip › S1_RAW_images/Fig 4 raw data/Fig 4 e HO-1 lane 8-13B.tif]

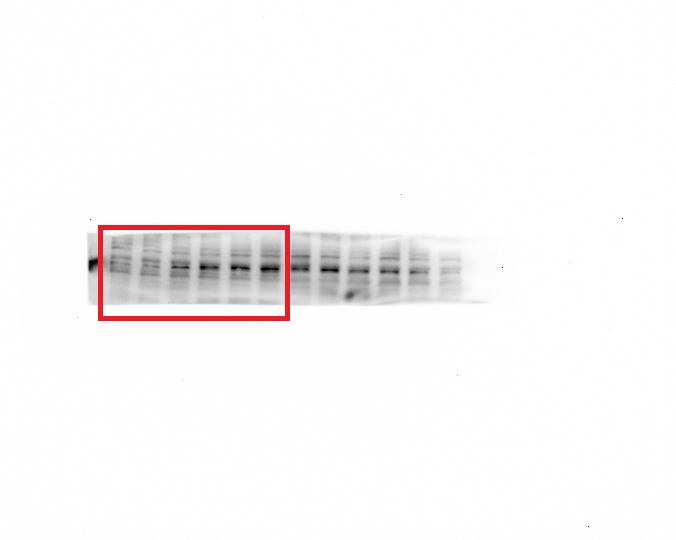

Supplement: S1 Raw images — (ZIP) [file pone.0322746.s004.zip › S1_RAW_images/Fig 4 raw data/Fig 4 e NRF2 lane 2-7.tif]

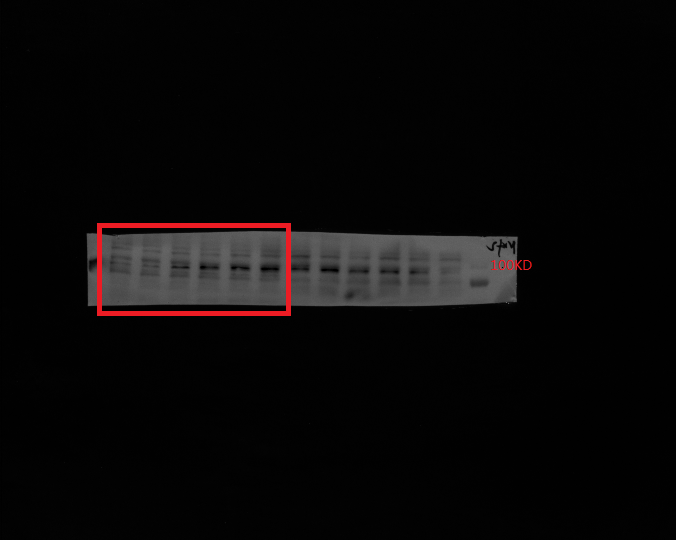

Supplement: S1 Raw images — (ZIP) [file pone.0322746.s004.zip › S1_RAW_images/Fig 4 raw data/Fig 4 e NRF2 lane 2-7B.tif]

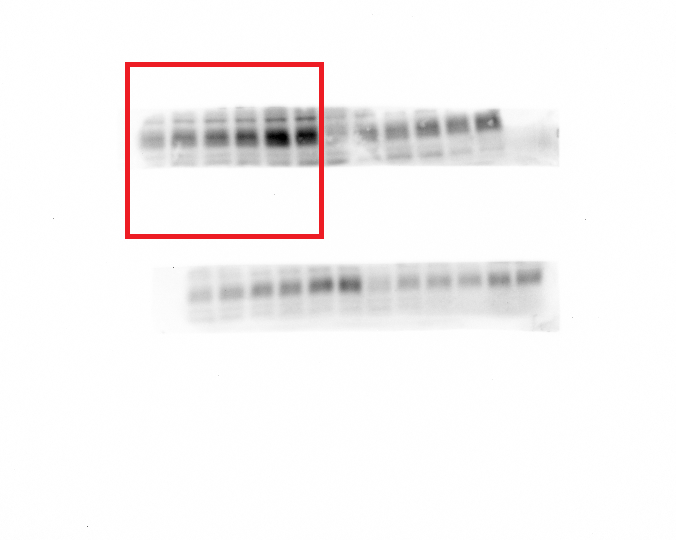

Supplement: S1 Raw images — (ZIP) [file pone.0322746.s004.zip › S1_RAW_images/Fig 4 raw data/Fig 4 e SLC7A11 lane 2- 5.tif]

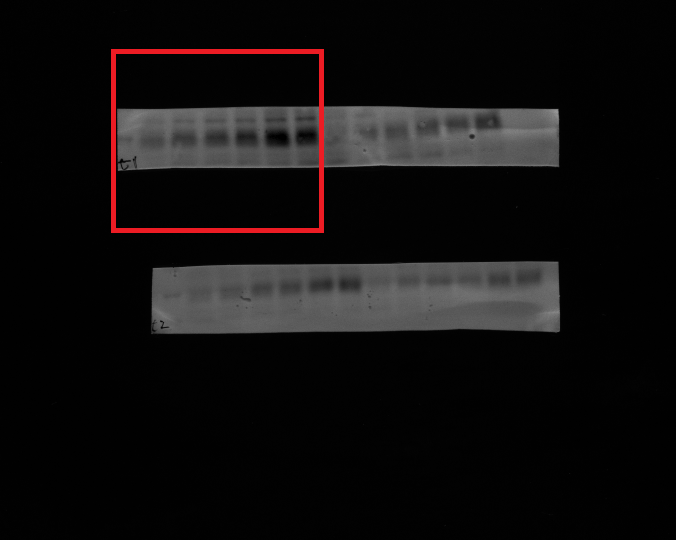

Supplement: S1 Raw images — (ZIP) [file pone.0322746.s004.zip › S1_RAW_images/Fig 4 raw data/Fig 4 e SLC7A11 lane 2- 5B.tif]

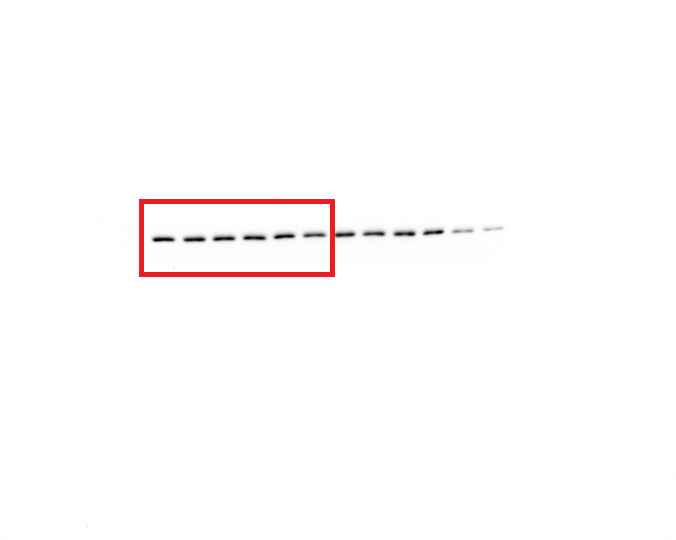

Supplement: S1 Raw images — (ZIP) [file pone.0322746.s004.zip › S1_RAW_images/Fig 4 raw data/Fig 4 e β-Tubulin lane 2-7.tif]

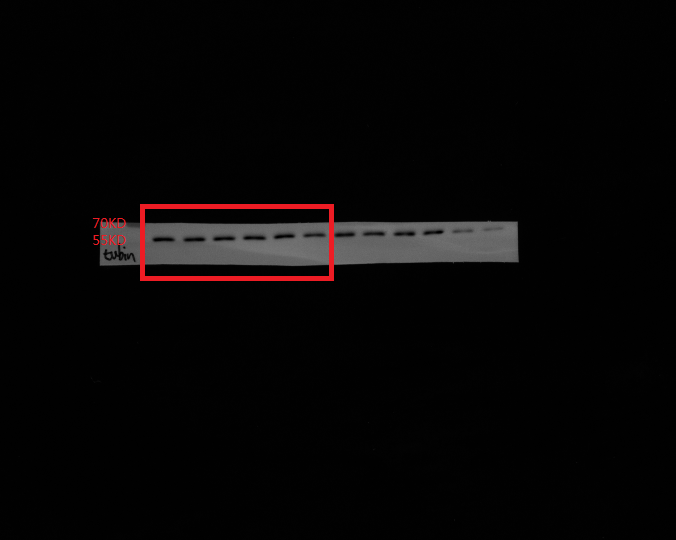

Supplement: S1 Raw images — (ZIP) [file pone.0322746.s004.zip › S1_RAW_images/Fig 4 raw data/Fig 4 e β-Tubulin lane 2-7B.tif]

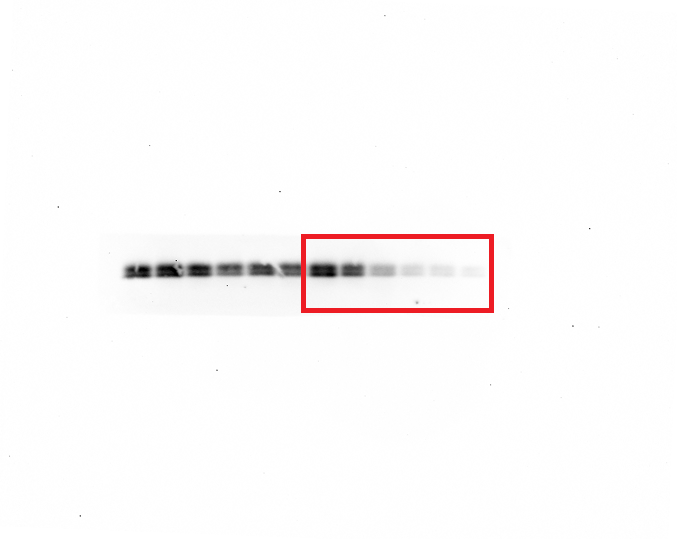

Supplement: S1 Raw images — (ZIP) [file pone.0322746.s004.zip › S1_RAW_images/Fig 4 raw data/Fig 4 f GPX4 lane 8-13.tif]

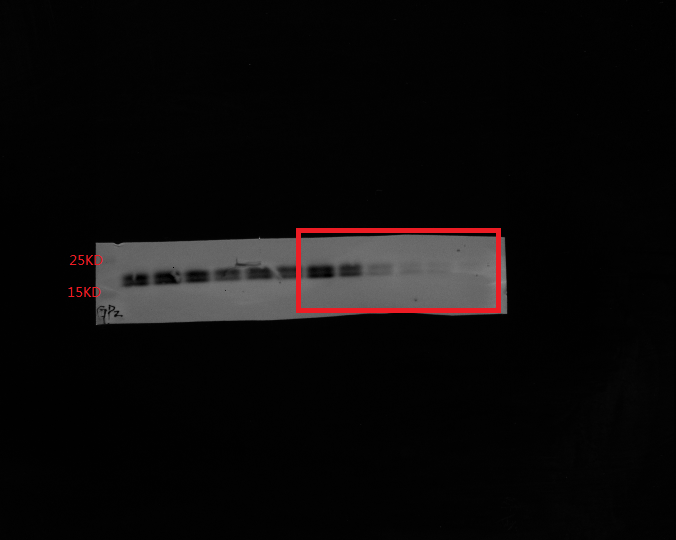

Supplement: S1 Raw images — (ZIP) [file pone.0322746.s004.zip › S1_RAW_images/Fig 4 raw data/Fig 4 f GPX4 lane 8-13B.tif]

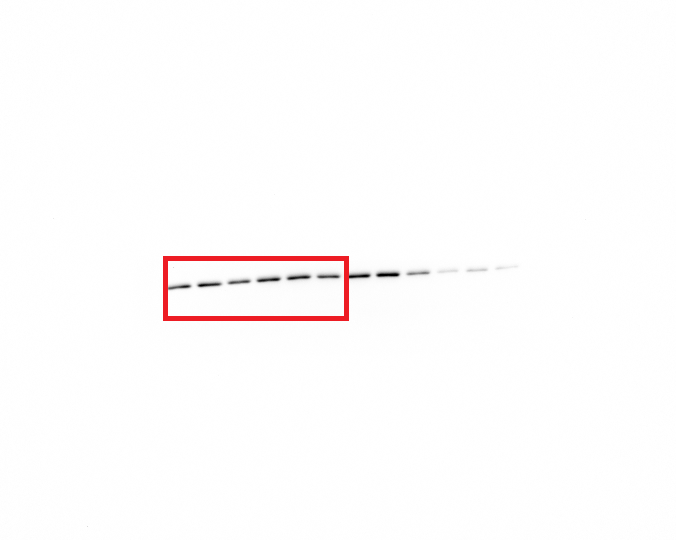

Supplement: S1 Raw images — (ZIP) [file pone.0322746.s004.zip › S1_RAW_images/Fig 4 raw data/Fig 4 f β-Tubulin lane 2-7.tif]

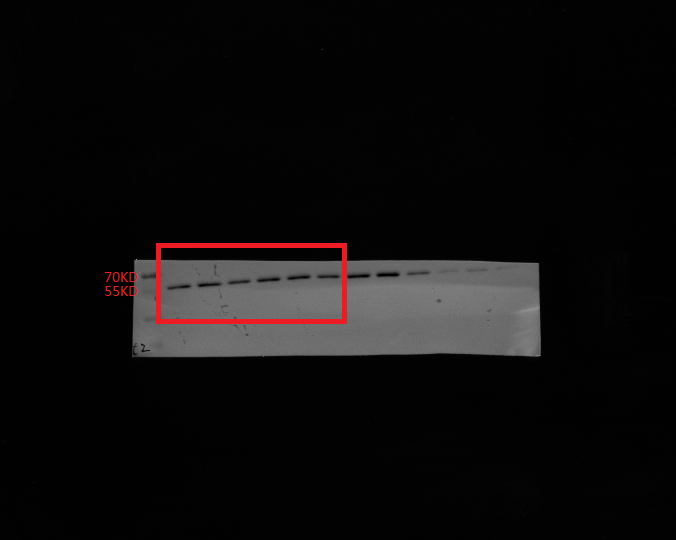

Supplement: S1 Raw images — (ZIP) [file pone.0322746.s004.zip › S1_RAW_images/Fig 4 raw data/Fig 4 f β-Tubulin lane 2-7B.tif]

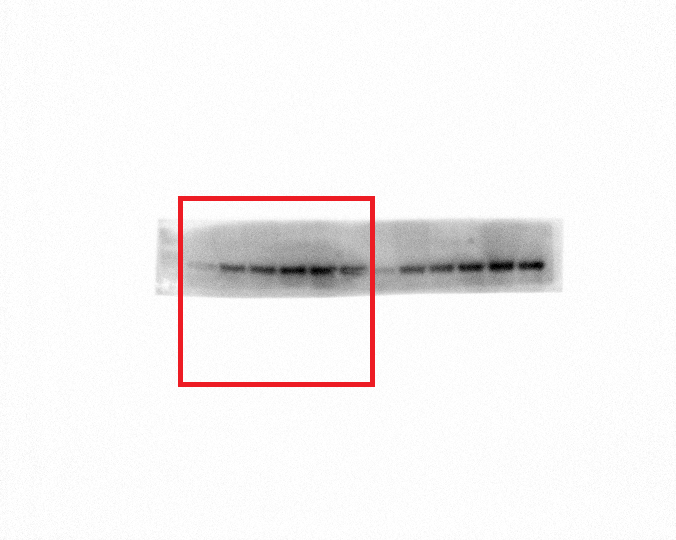

Supplement: S1 Raw images — (ZIP) [file pone.0322746.s004.zip › S1_RAW_images/Fig 4 raw data/Fig 4 f HO-1 lane 2-7.tif]

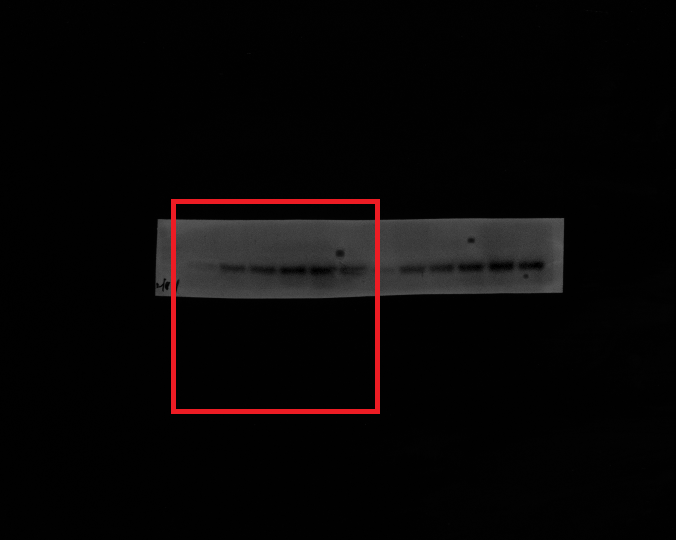

Supplement: S1 Raw images — (ZIP) [file pone.0322746.s004.zip › S1_RAW_images/Fig 4 raw data/Fig 4 f HO-1 lane 2-7B.tif]

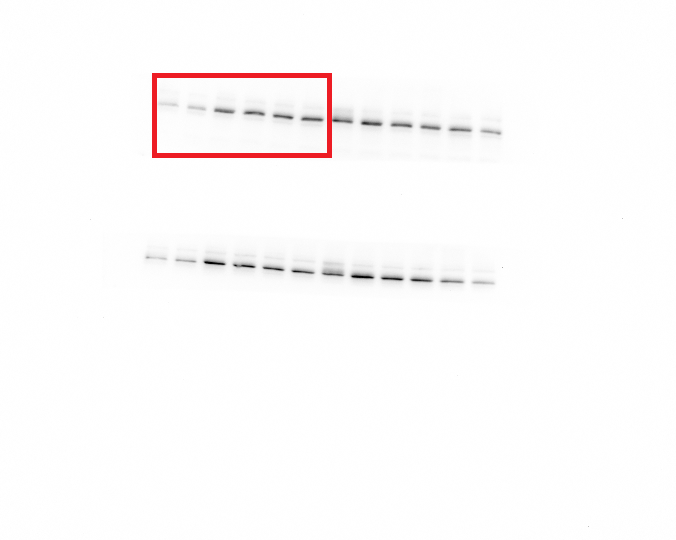

Supplement: S1 Raw images — (ZIP) [file pone.0322746.s004.zip › S1_RAW_images/Fig 4 raw data/Fig 4 f NRF2 lane 2-7.tif]

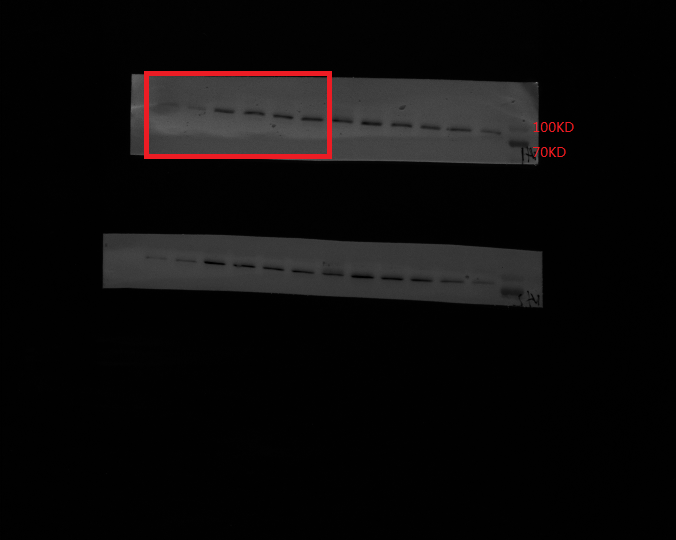

Supplement: S1 Raw images — (ZIP) [file pone.0322746.s004.zip › S1_RAW_images/Fig 4 raw data/Fig 4 f NRF2 lane 2-7B.tif]

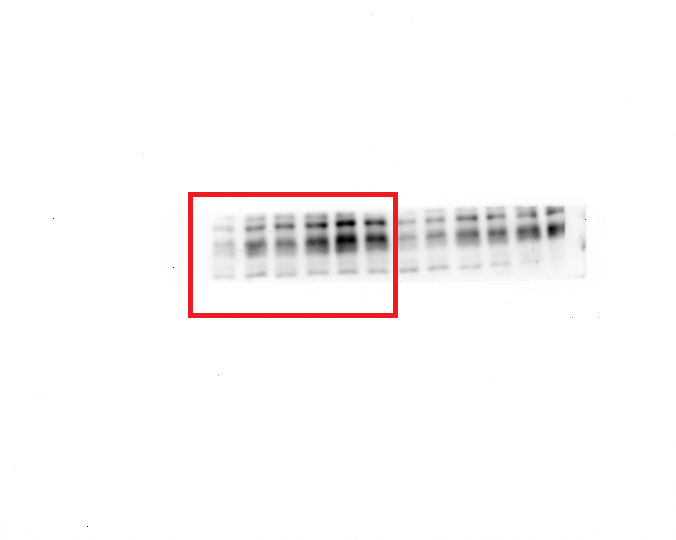

Supplement: S1 Raw images — (ZIP) [file pone.0322746.s004.zip › S1_RAW_images/Fig 4 raw data/Fig 4 f SLC7A11 lane 2-7.tif]

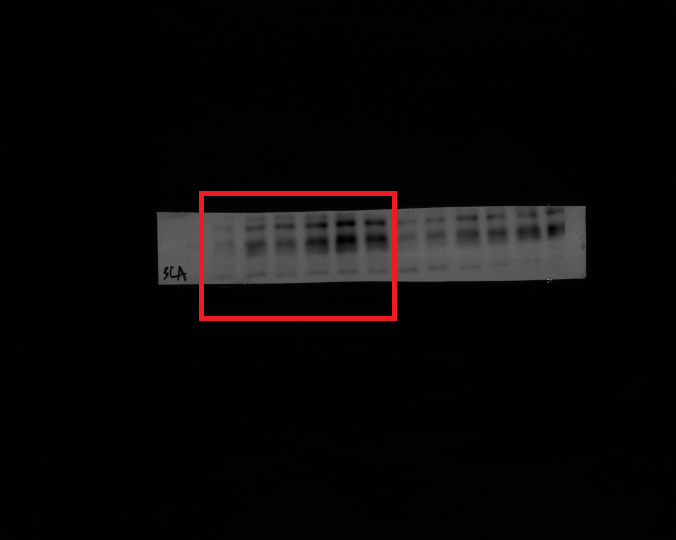

Supplement: S1 Raw images — (ZIP) [file pone.0322746.s004.zip › S1_RAW_images/Fig 4 raw data/Fig 4 f SLC7A11 lane 2-7B.tif]

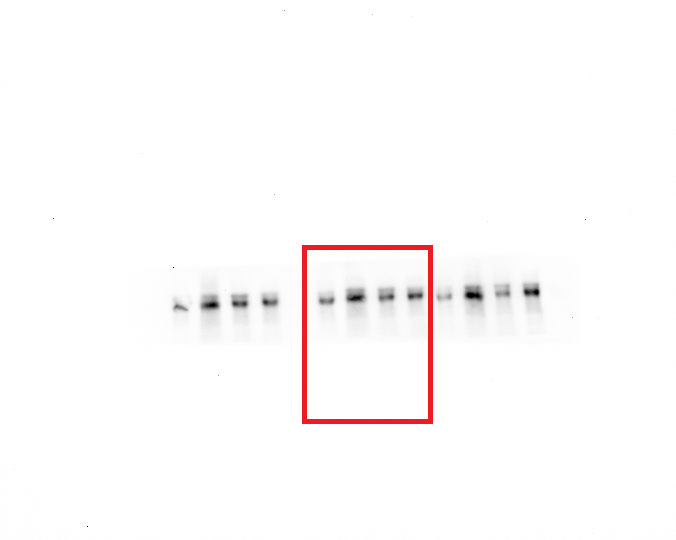

Supplement: S1 Raw images — (ZIP) [file pone.0322746.s004.zip › S1_RAW_images/Fig 5 raw data/Fig 5 A NRF2 lane7-10.tif]

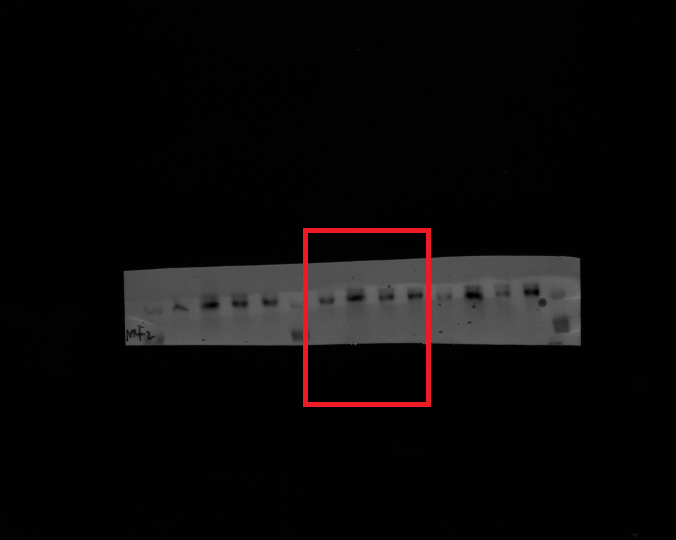

Supplement: S1 Raw images — (ZIP) [file pone.0322746.s004.zip › S1_RAW_images/Fig 5 raw data/Fig 5 A NRF2 lane7-10B.tif]

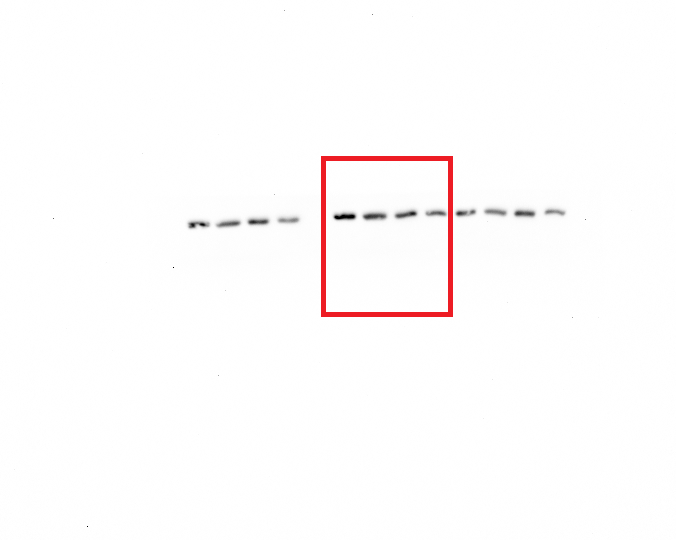

Supplement: S1 Raw images — (ZIP) [file pone.0322746.s004.zip › S1_RAW_images/Fig 5 raw data/Fig 5 A TUBLIN2 lane7-10.tif]

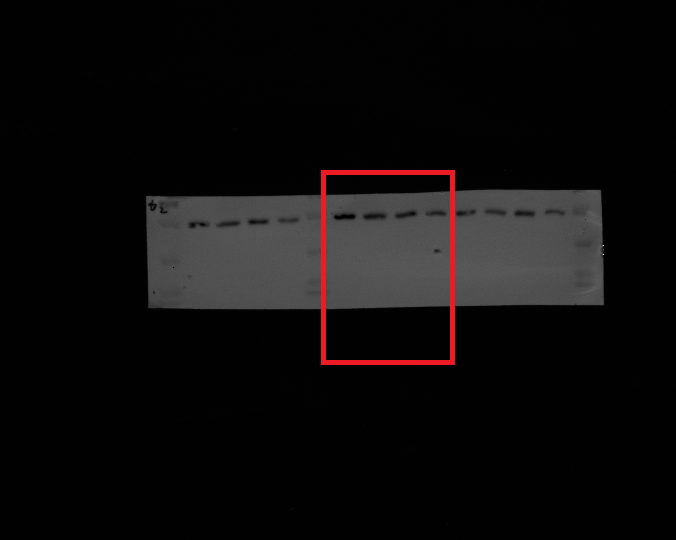

Supplement: S1 Raw images — (ZIP) [file pone.0322746.s004.zip › S1_RAW_images/Fig 5 raw data/Fig 5 A TUBLIN2 lane7-10B.tif]

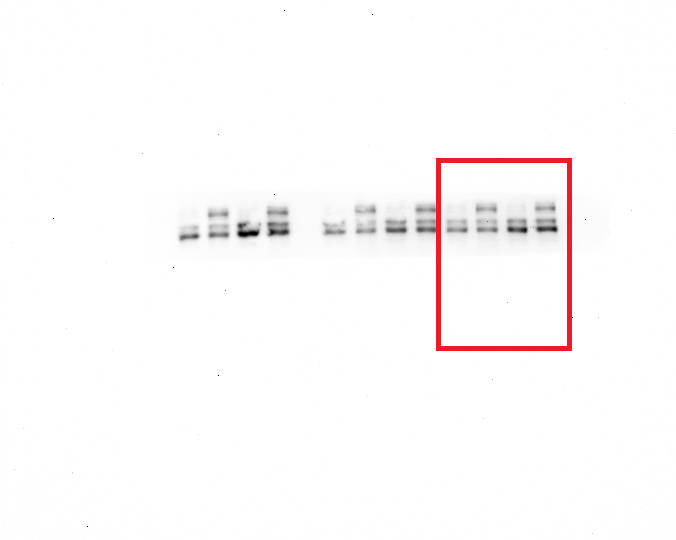

Supplement: S1 Raw images — (ZIP) [file pone.0322746.s004.zip › S1_RAW_images/Fig 5 raw data/Fig 5 D NRF2 lane11-14.tif]

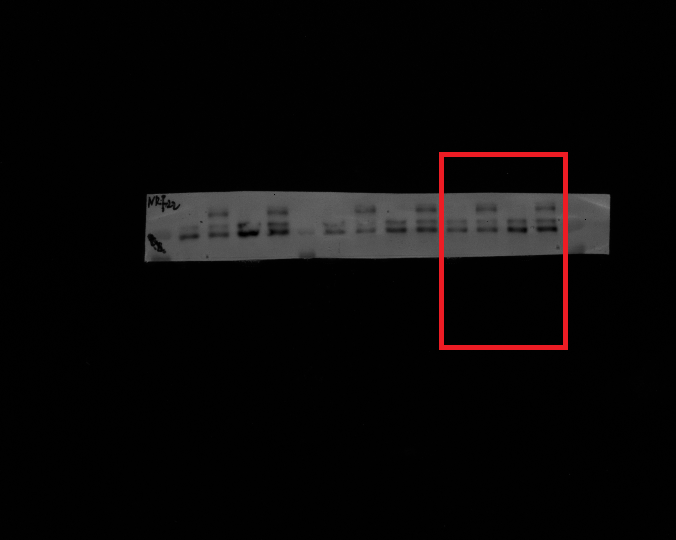

Supplement: S1 Raw images — (ZIP) [file pone.0322746.s004.zip › S1_RAW_images/Fig 5 raw data/Fig 5 D NRF2 lane11-14B.tif]

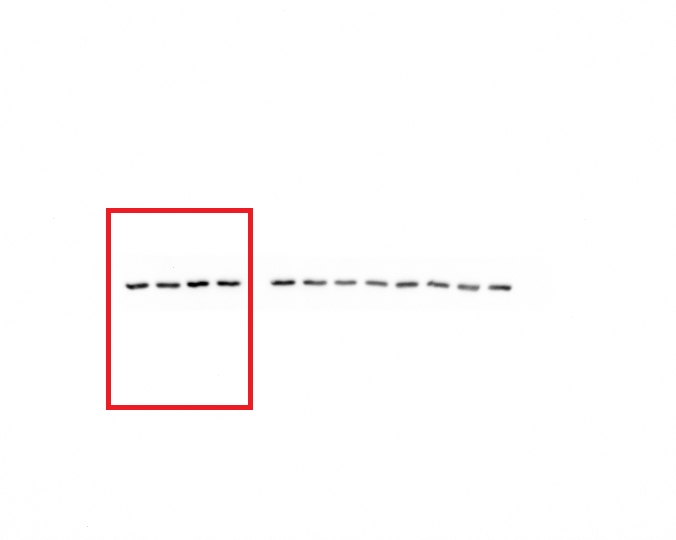

Supplement: S1 Raw images — (ZIP) [file pone.0322746.s004.zip › S1_RAW_images/Fig 5 raw data/Fig 5 D TUBLIN2 lane 2-5.tif]

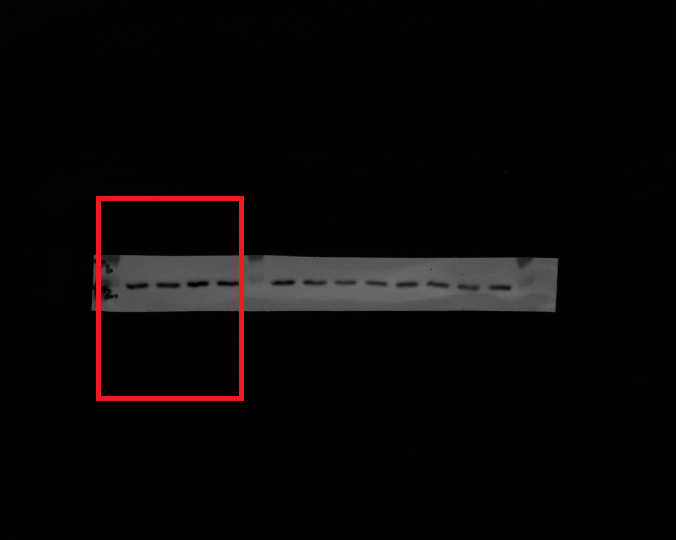

Supplement: S1 Raw images — (ZIP) [file pone.0322746.s004.zip › S1_RAW_images/Fig 5 raw data/Fig 5 D TUBLIN2 lane 2-5B.tif]

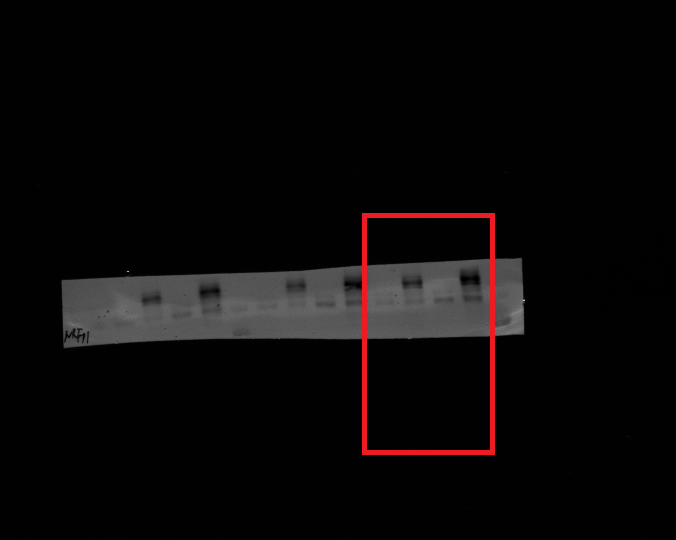

Supplement: S1 Raw images — (ZIP) [file pone.0322746.s004.zip › S1_RAW_images/Fig 5 raw data/Fig 5 E NRF2 lane11-14 B.tif]

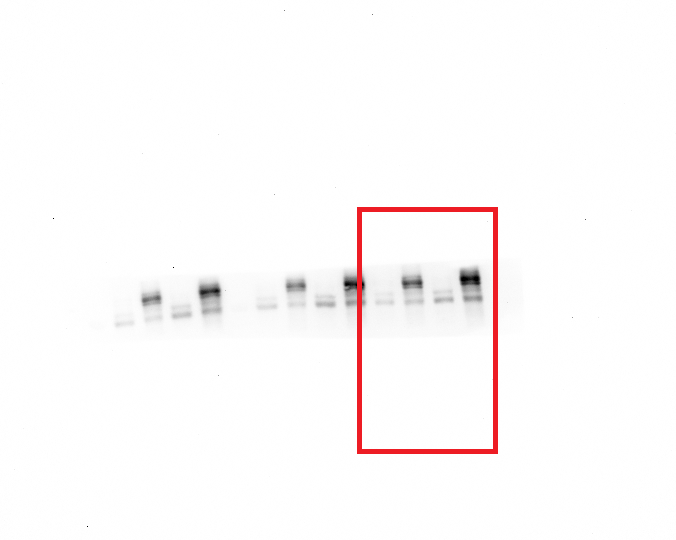

Supplement: S1 Raw images — (ZIP) [file pone.0322746.s004.zip › S1_RAW_images/Fig 5 raw data/Fig 5 E NRF2 lane11-14.tif]

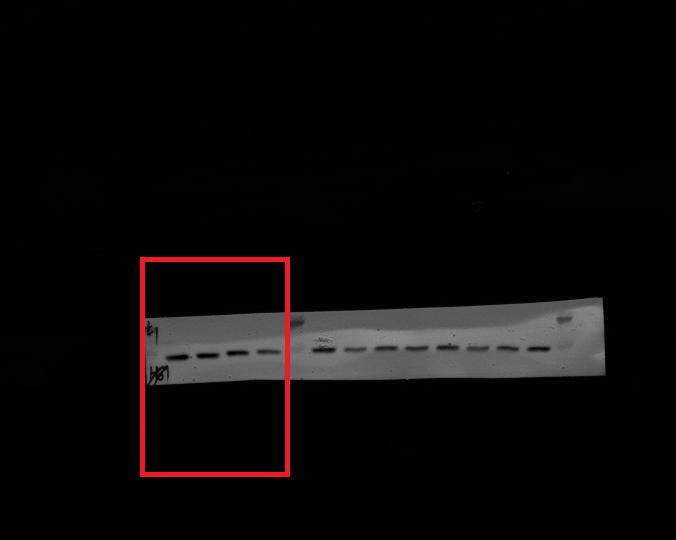

Supplement: S1 Raw images — (ZIP) [file pone.0322746.s004.zip › S1_RAW_images/Fig 5 raw data/Fig 5 E tublin lane2-5 B.tif]

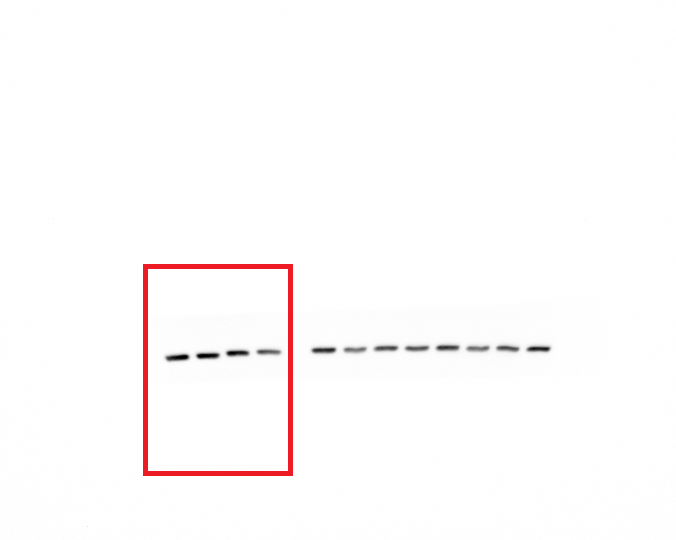

Supplement: S1 Raw images — (ZIP) [file pone.0322746.s004.zip › S1_RAW_images/Fig 5 raw data/Fig 5 E tublin lane2-5.tif]

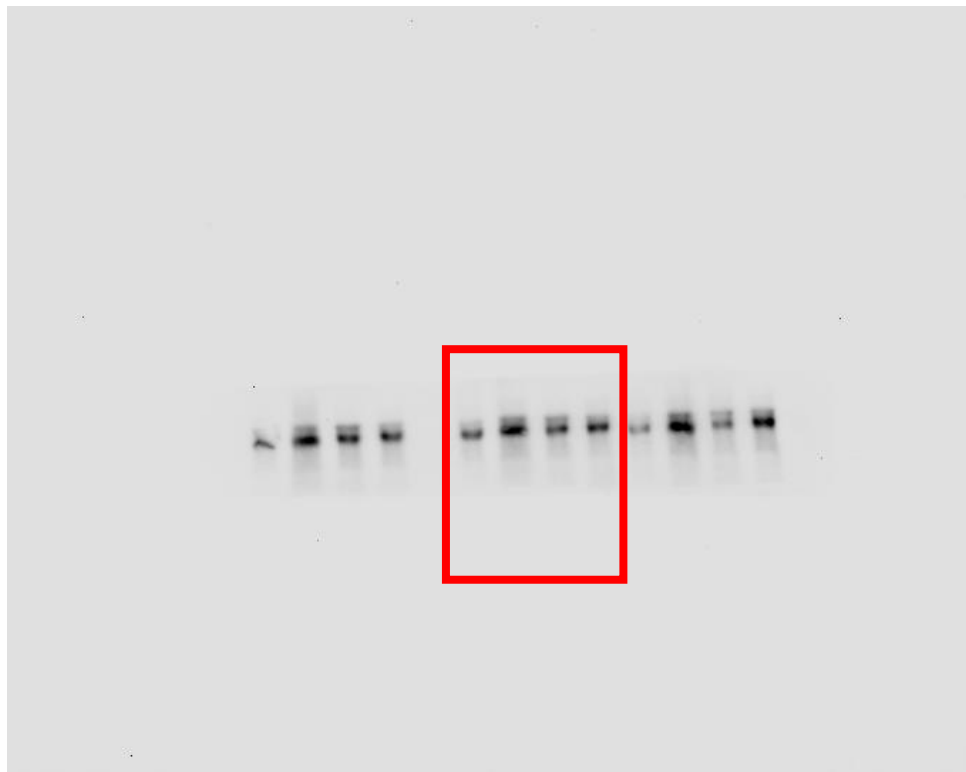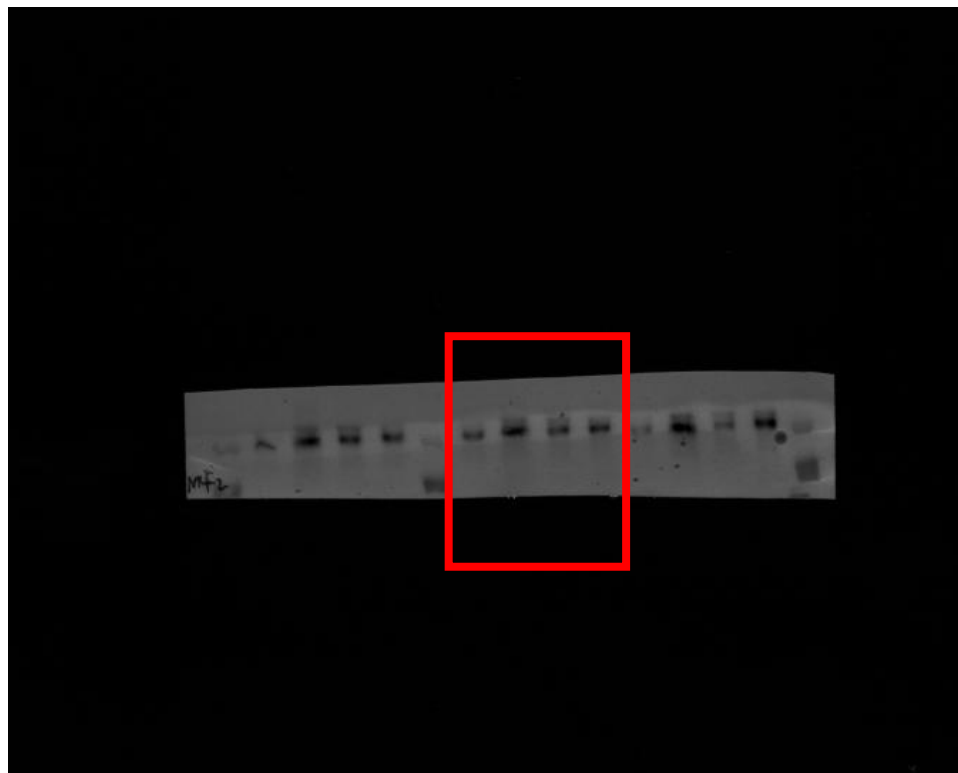

Fig 5 A NRF2 lane7-10

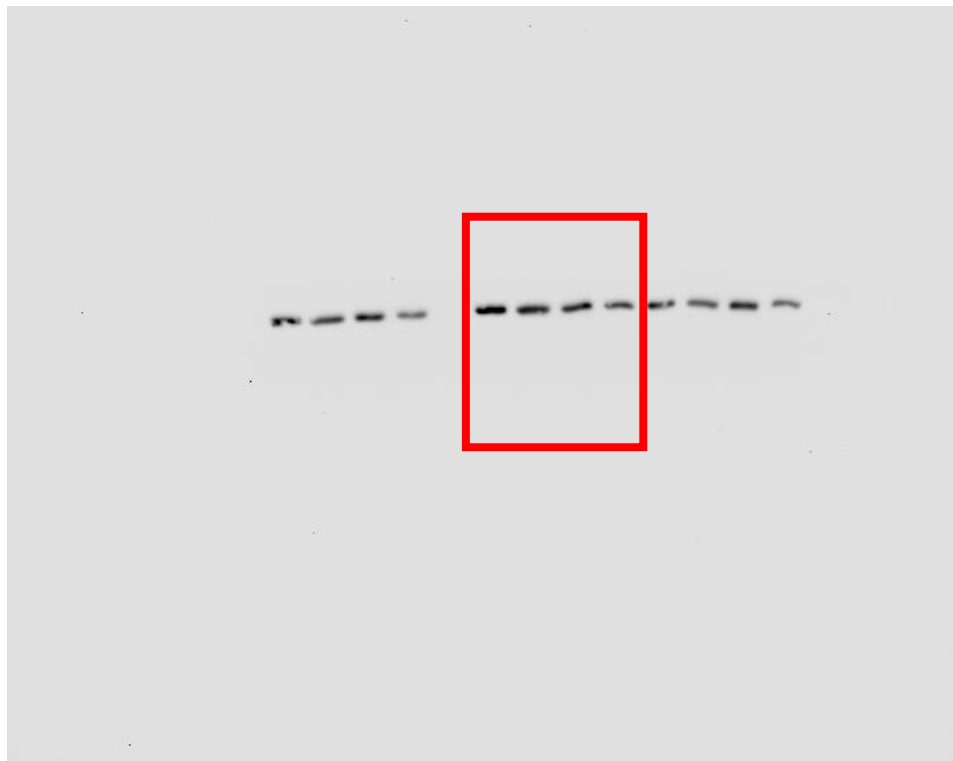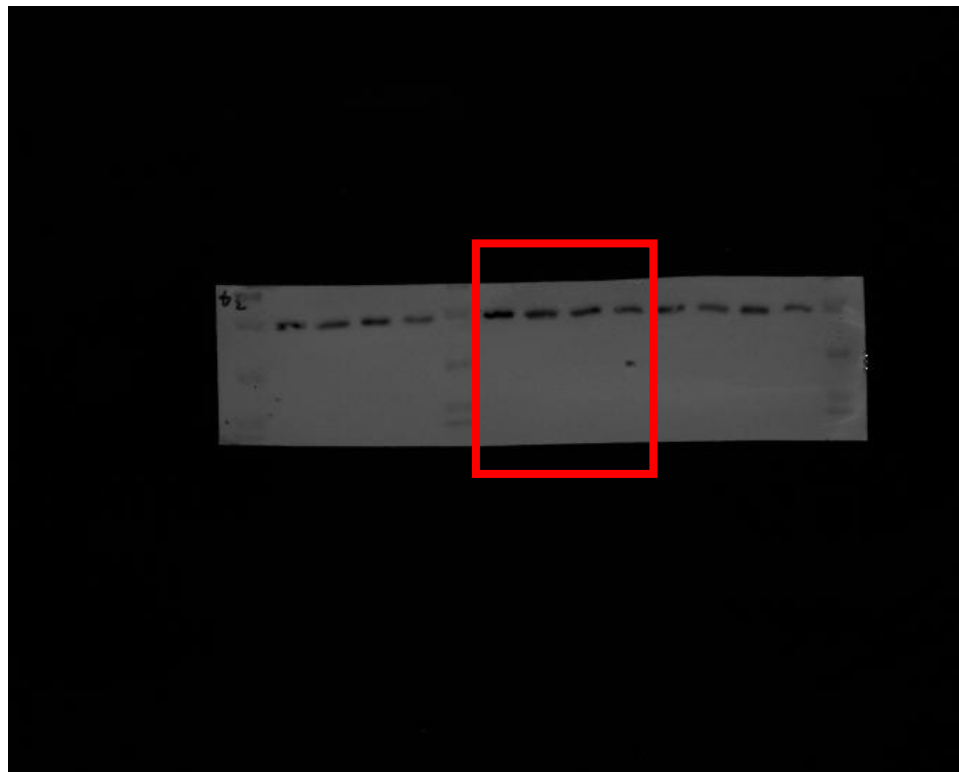

Fig 5 A tublin lane7-10

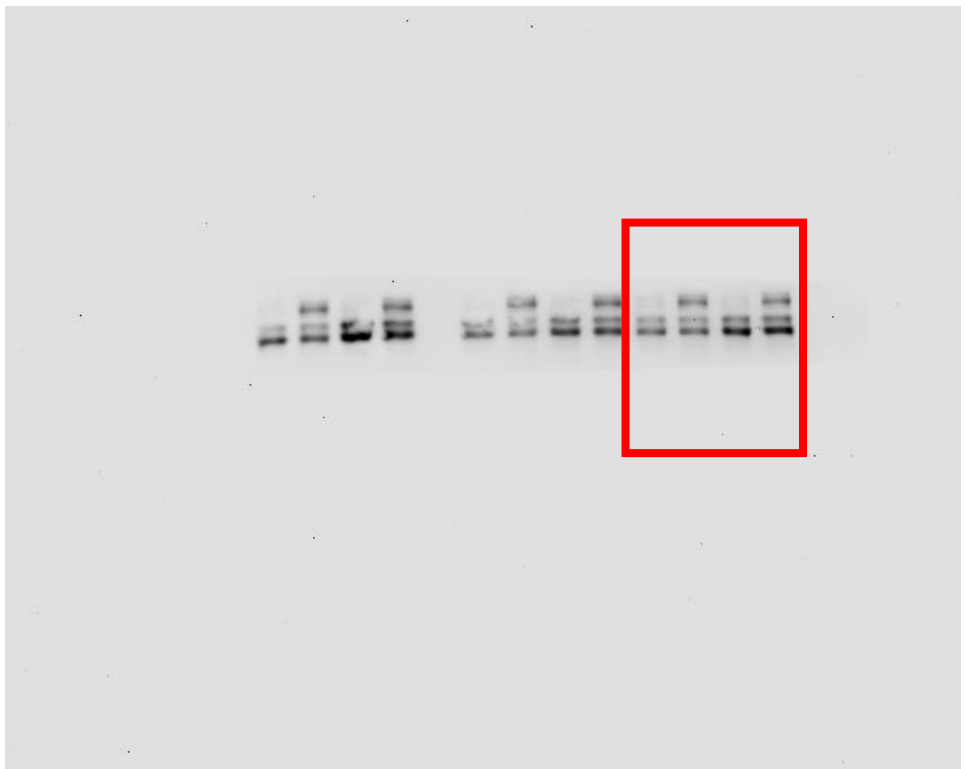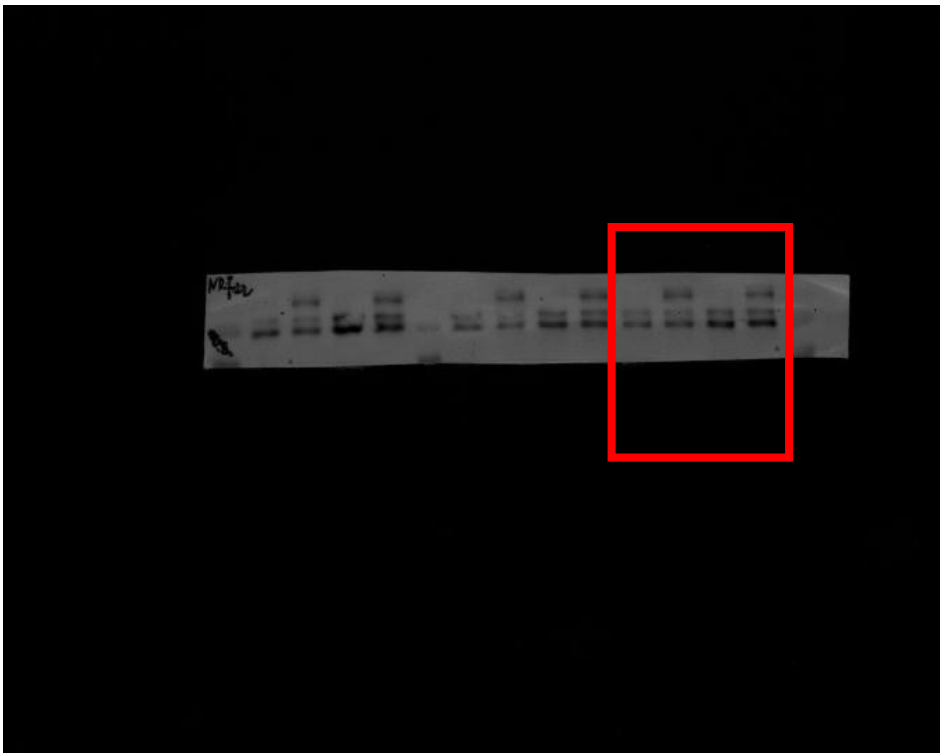

Fig 5 D NRF2 lane11-14

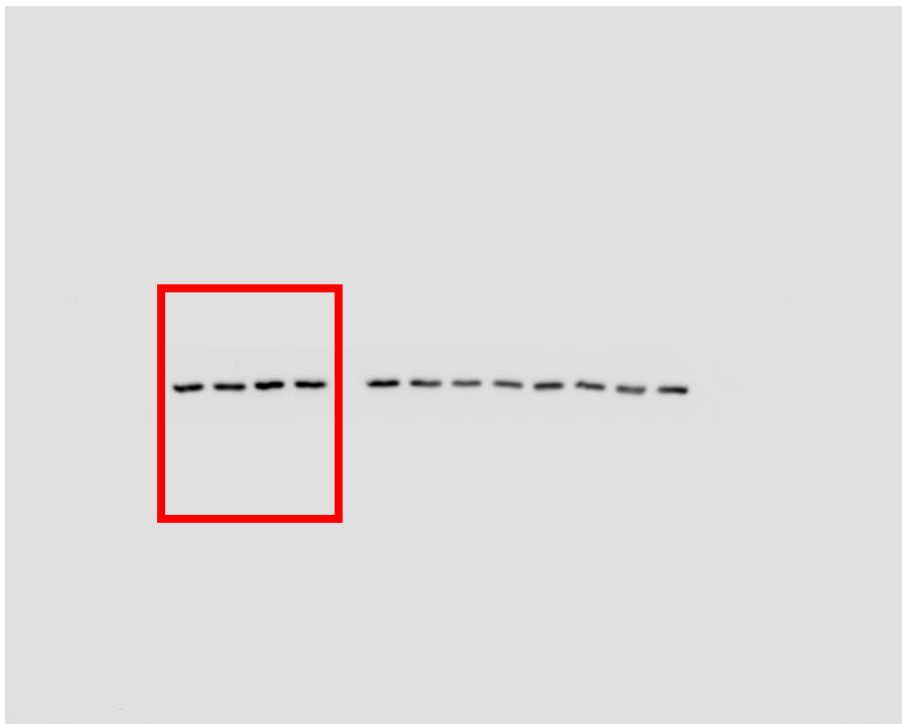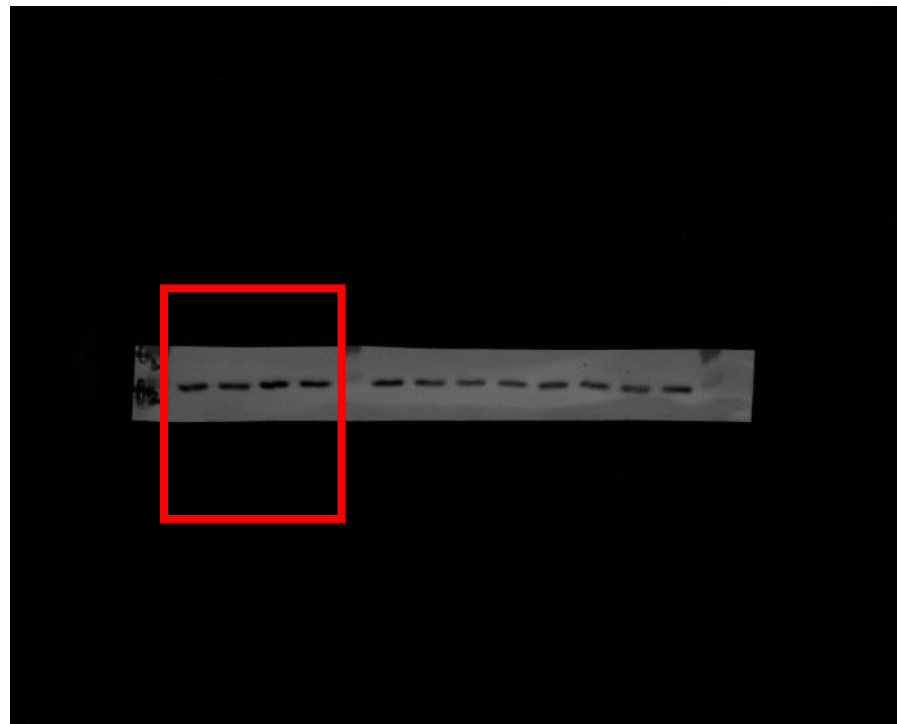

Fig 5 D tublin lane2-5

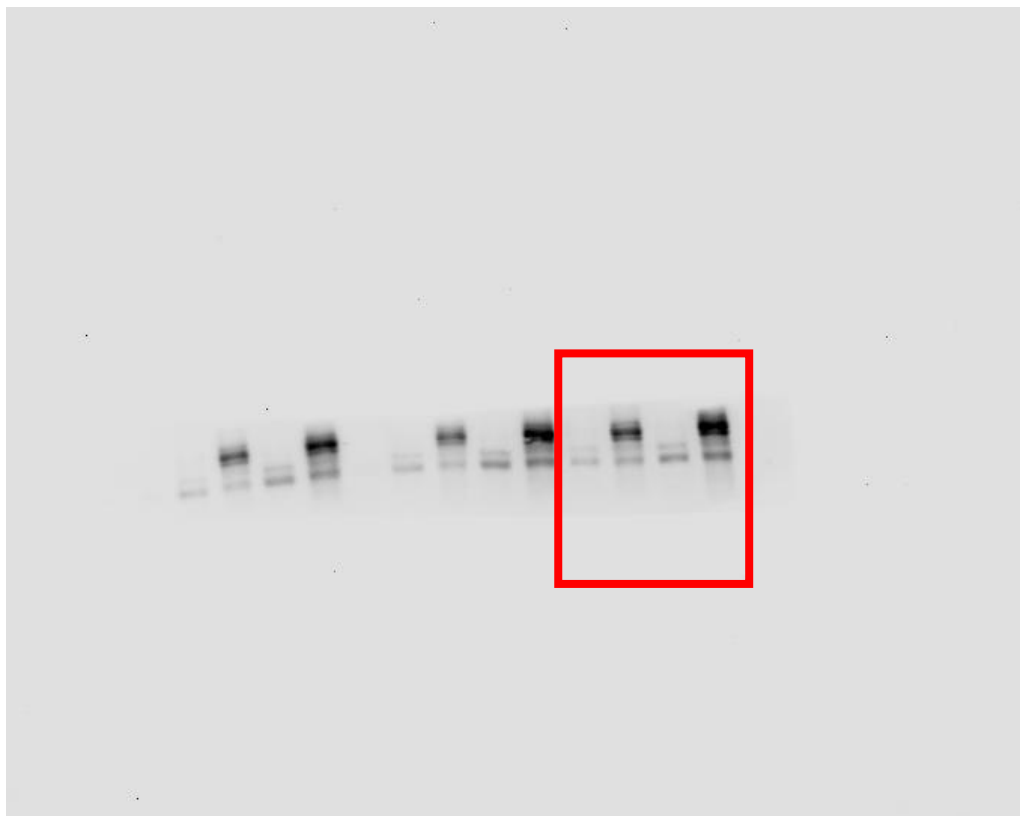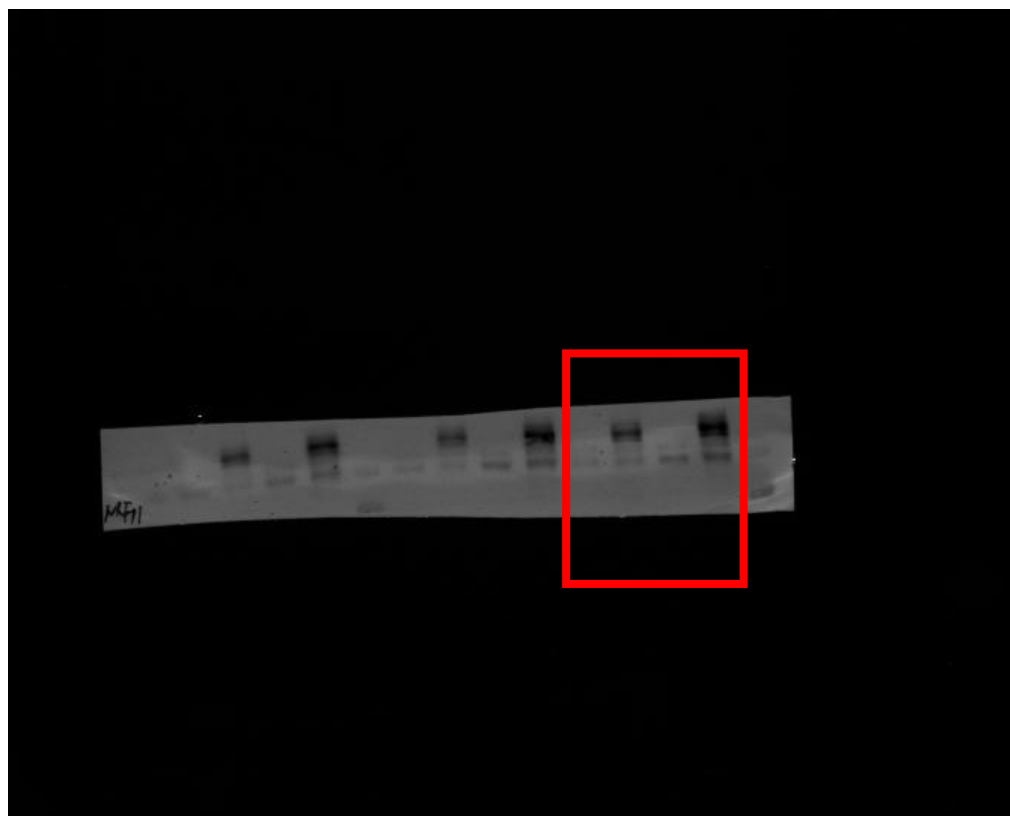

Fig 5 E NRF2 lane11-14

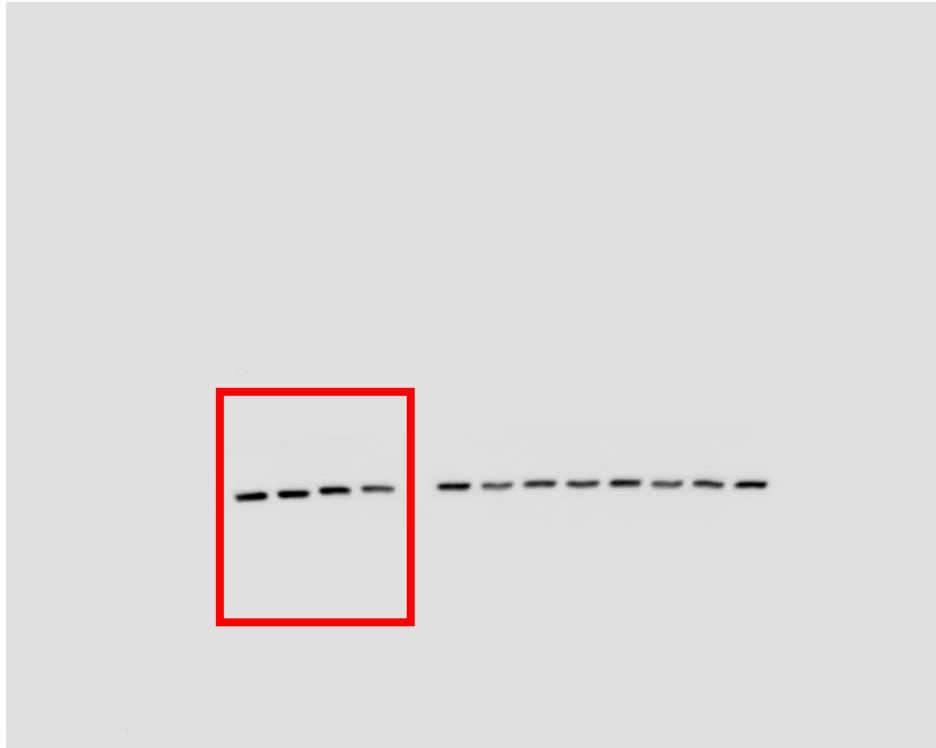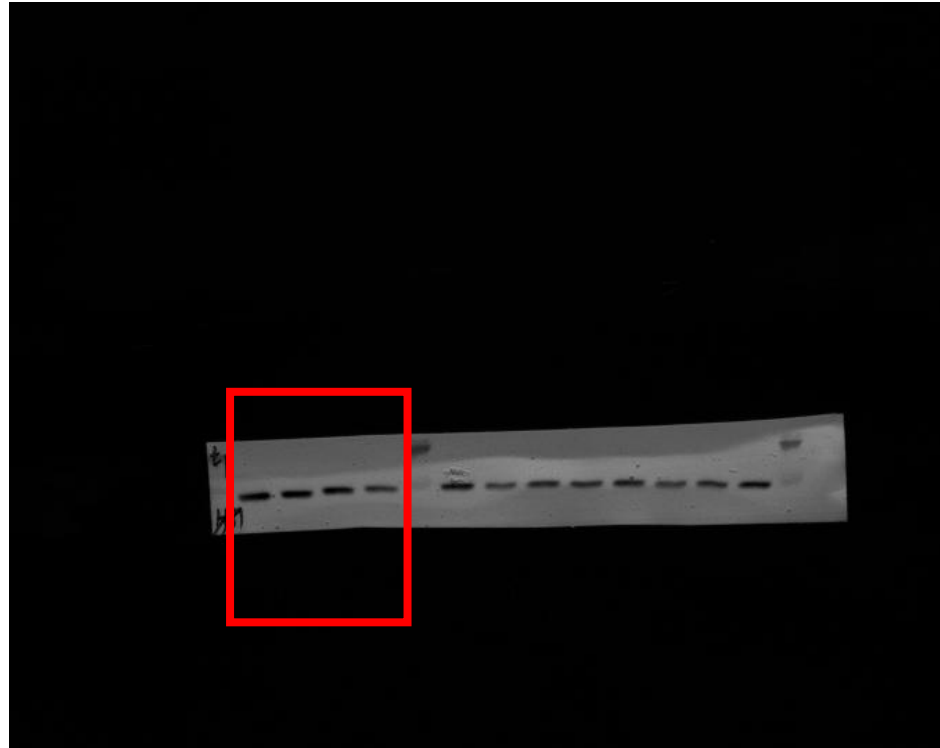

Fig 5 E tublin lane2-5

Supplement: S1 Raw images — (ZIP) [file pone.0322746.s004.zip › S1_RAW_images/Fig 5 raw data.pdf]

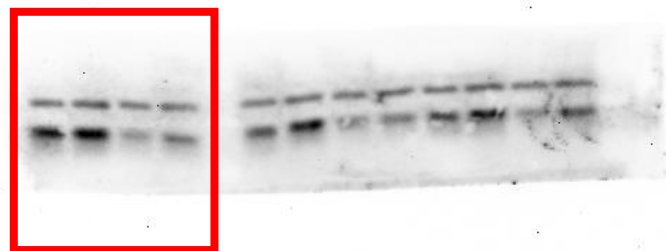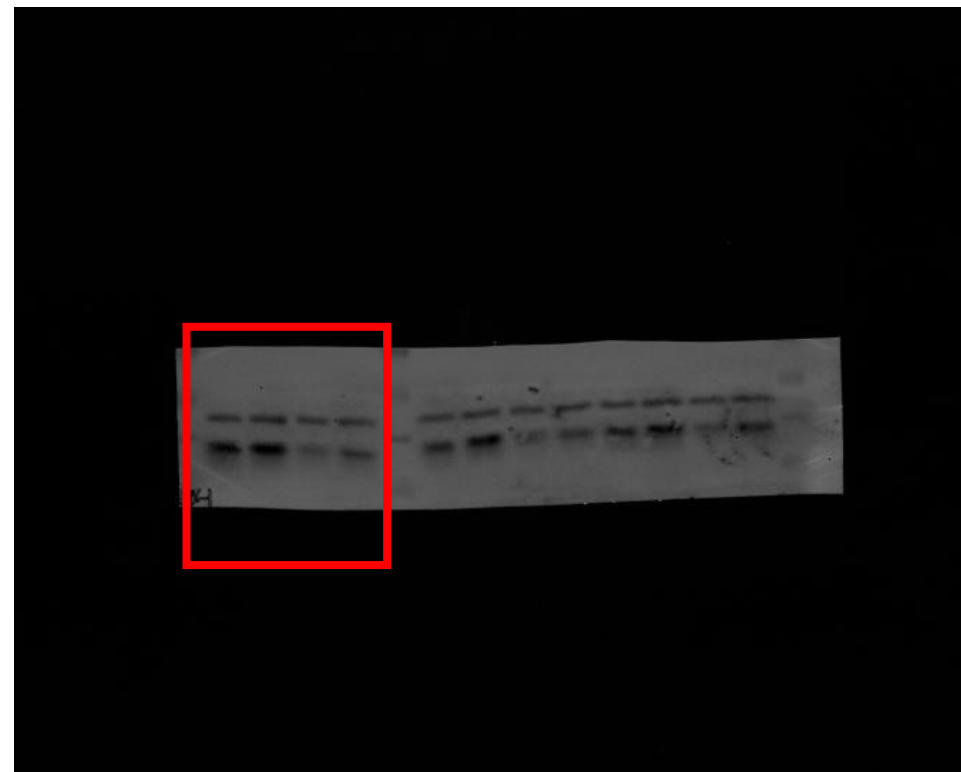

Figs 3 A GPX4 lane2-5

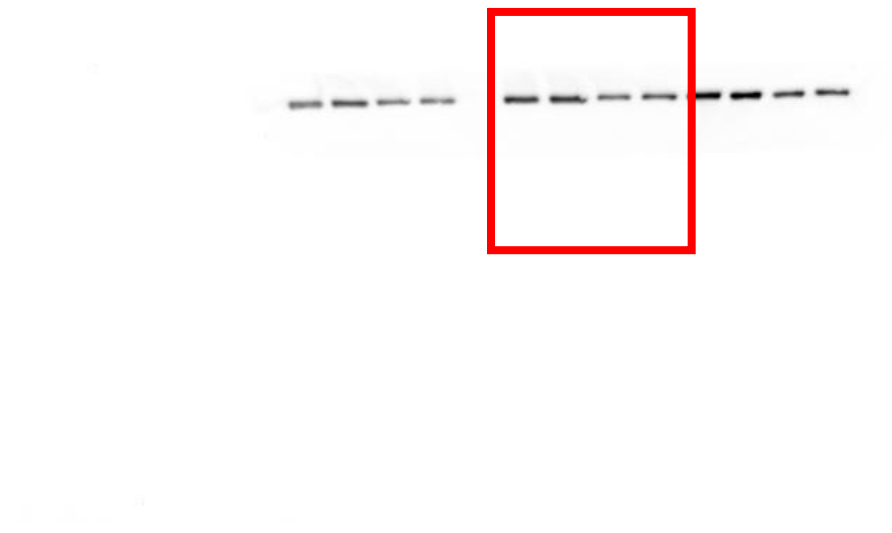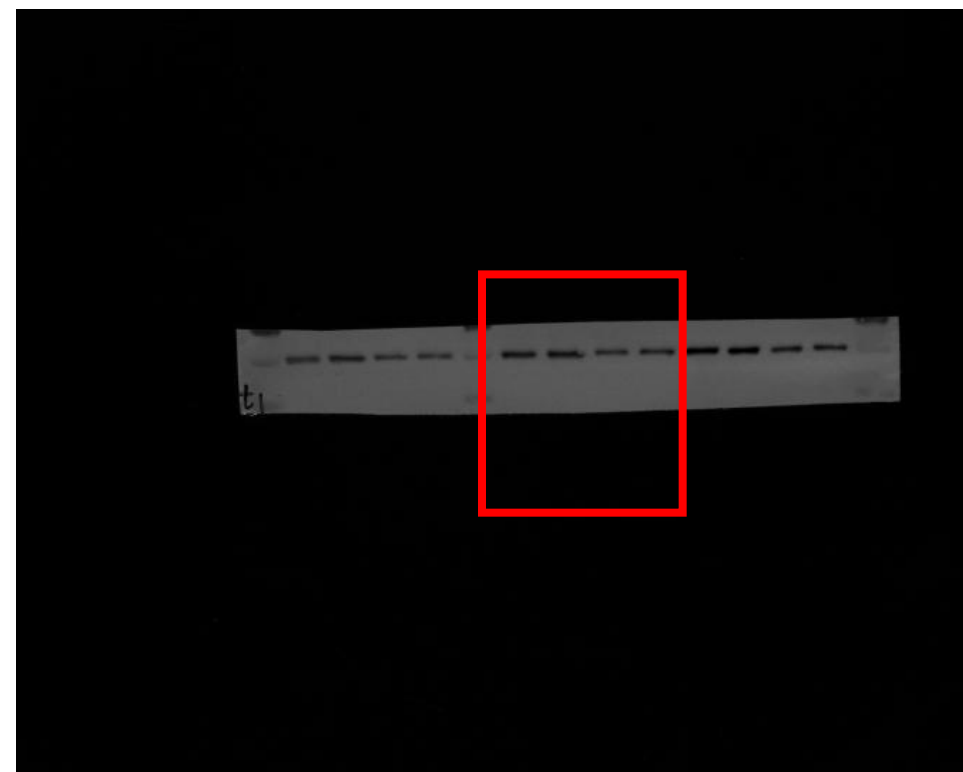

Figs 3 A TUBLIN lane7-10

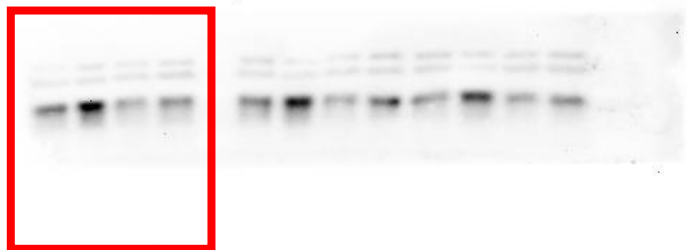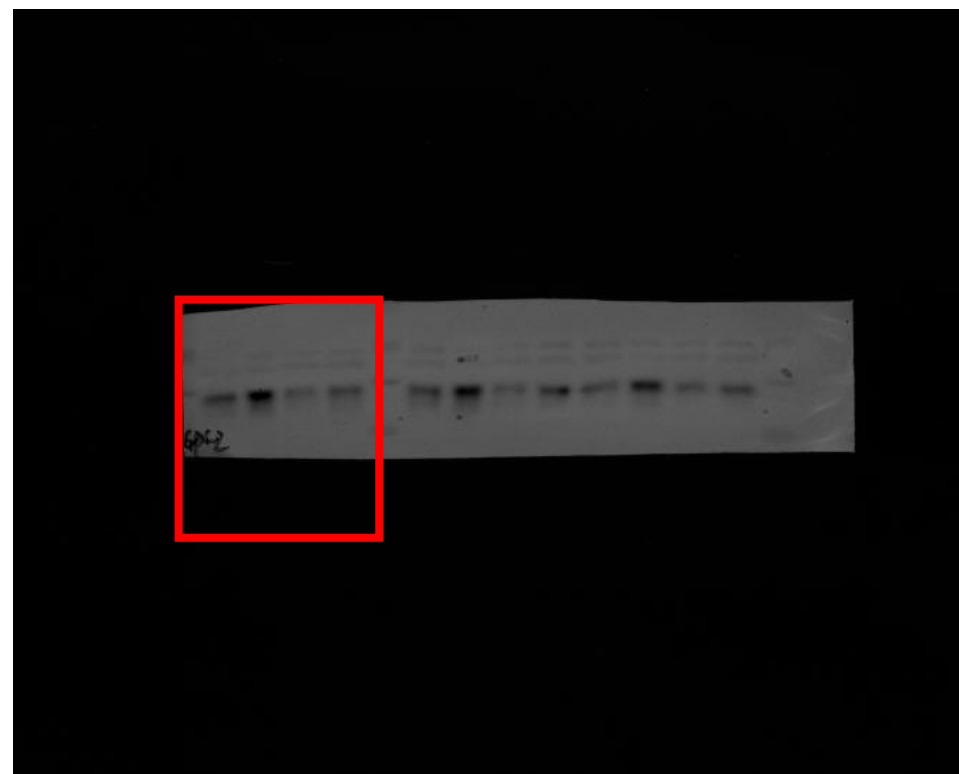

Figs 3 B GPX4 lane2-5

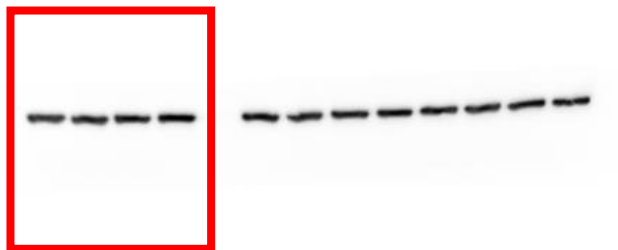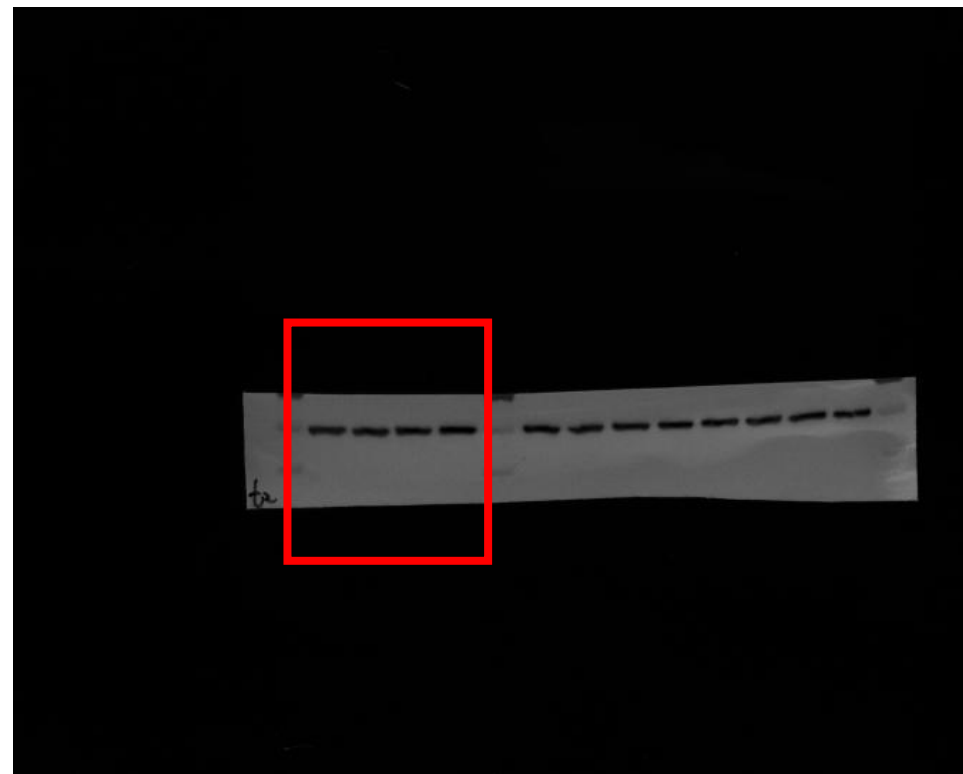

Figs 3 B TUBLIN lane2-5

Supplement: S1 Raw images — (ZIP) [file pone.0322746.s004.zip › S1_RAW_images/FigS 3 raw data.pdf]

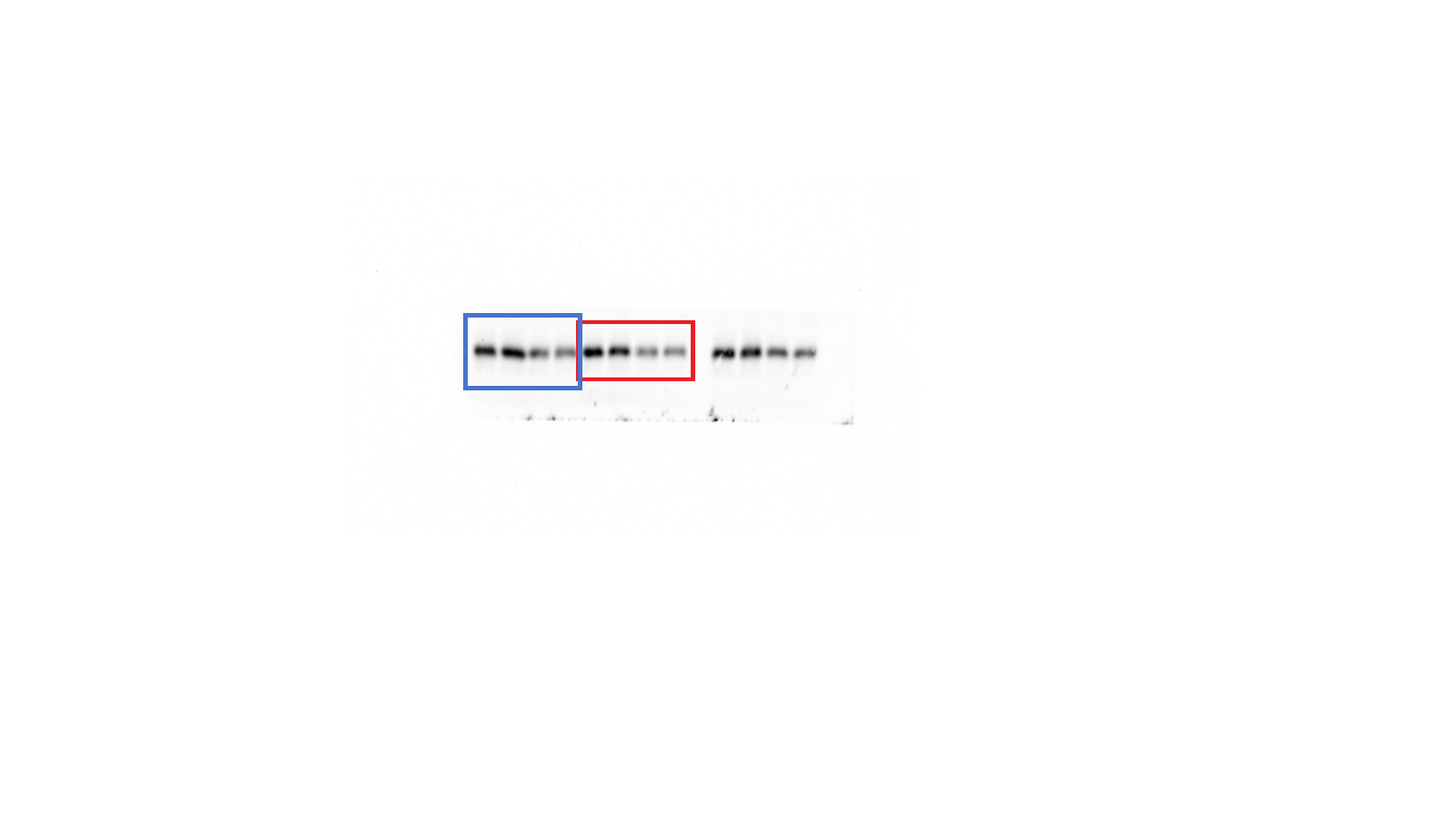

Supplement: S1 Raw images — (ZIP) [file pone.0322746.s004.zip › S1_RAW_images/fig 6 raw data/Fig 6 E GPX4 lane 6-9.tif]

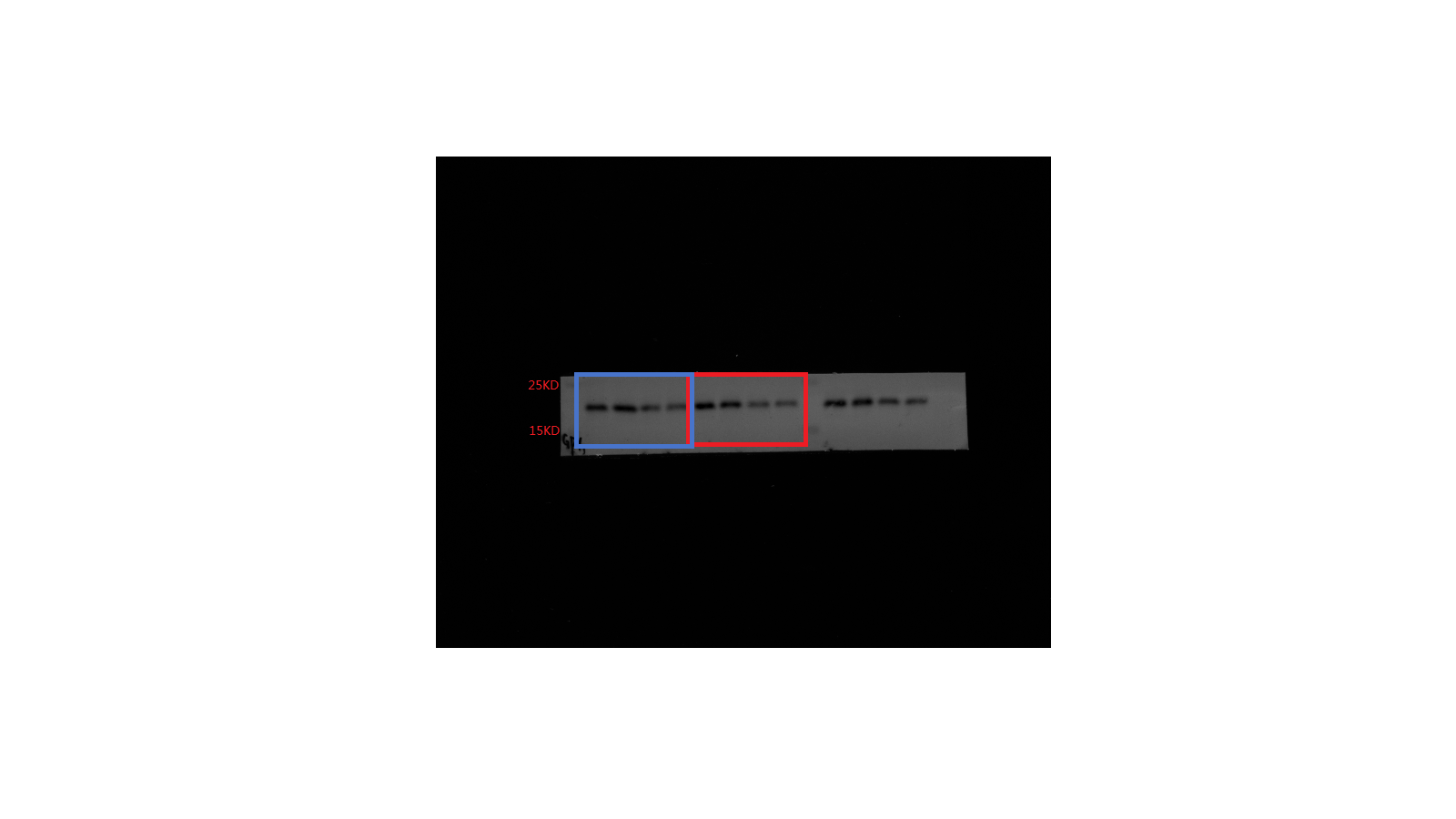

Supplement: S1 Raw images — (ZIP) [file pone.0322746.s004.zip › S1_RAW_images/fig 6 raw data/Fig 6 E GPX4 lane 6-9B.tif]

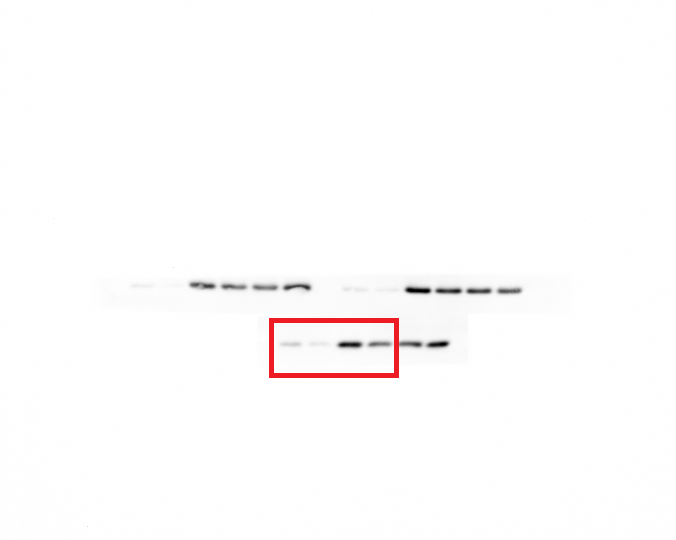

Supplement: S1 Raw images — (ZIP) [file pone.0322746.s004.zip › S1_RAW_images/fig 6 raw data/Fig 6 E HO-1 lane 2-5.tif]

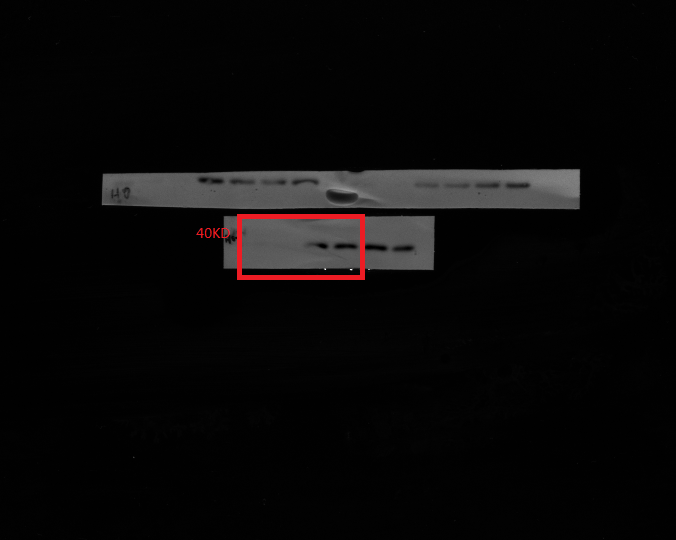

Supplement: S1 Raw images — (ZIP) [file pone.0322746.s004.zip › S1_RAW_images/fig 6 raw data/Fig 6 E HO-1 lane 2-5B.tif]

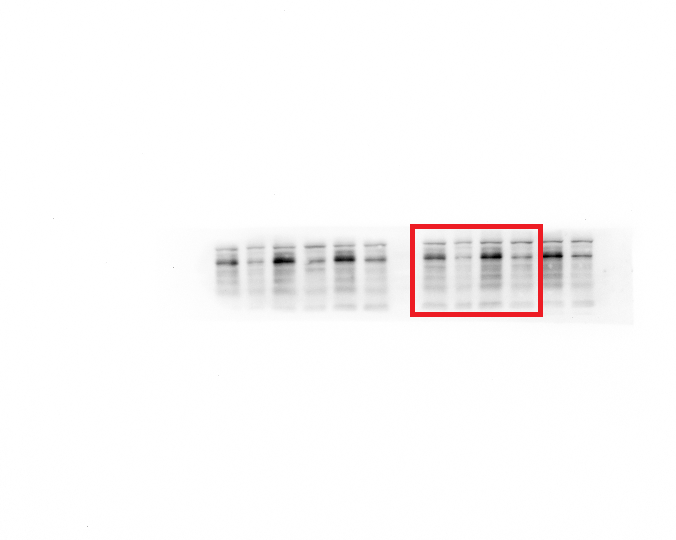

Supplement: S1 Raw images — (ZIP) [file pone.0322746.s004.zip › S1_RAW_images/fig 6 raw data/Fig 6 E NRF2 lane 9-12.tif]

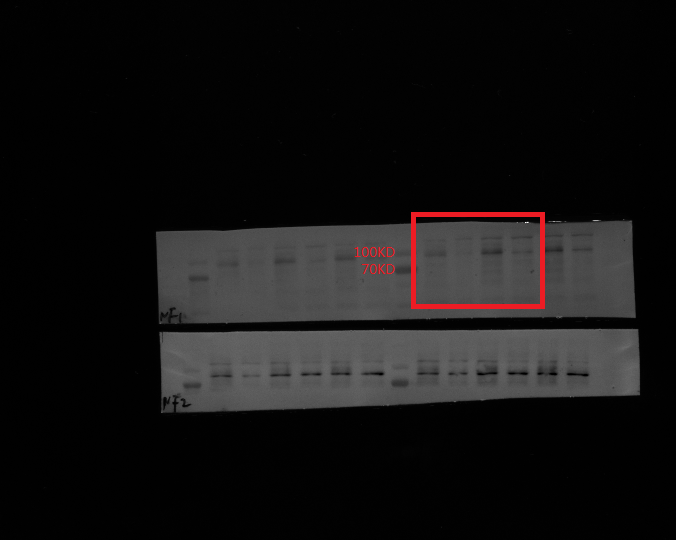

Supplement: S1 Raw images — (ZIP) [file pone.0322746.s004.zip › S1_RAW_images/fig 6 raw data/Fig 6 E NRF2 lane 9-12B.tif]

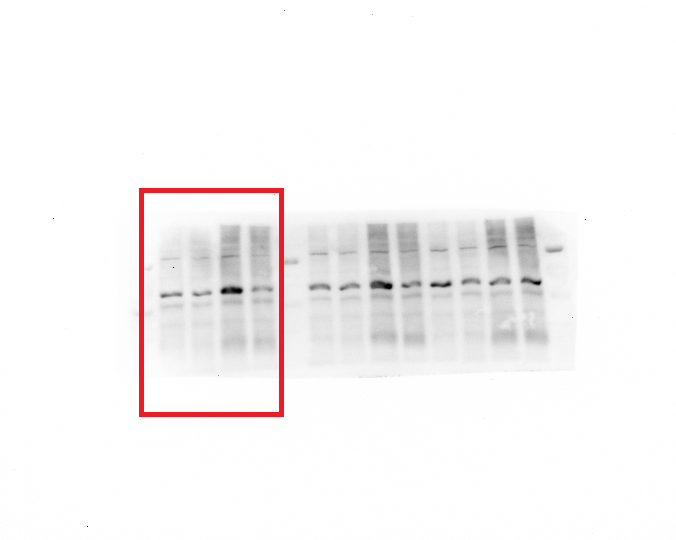

Supplement: S1 Raw images — (ZIP) [file pone.0322746.s004.zip › S1_RAW_images/fig 6 raw data/Fig 6 E lane 2-5SLC2.tif]

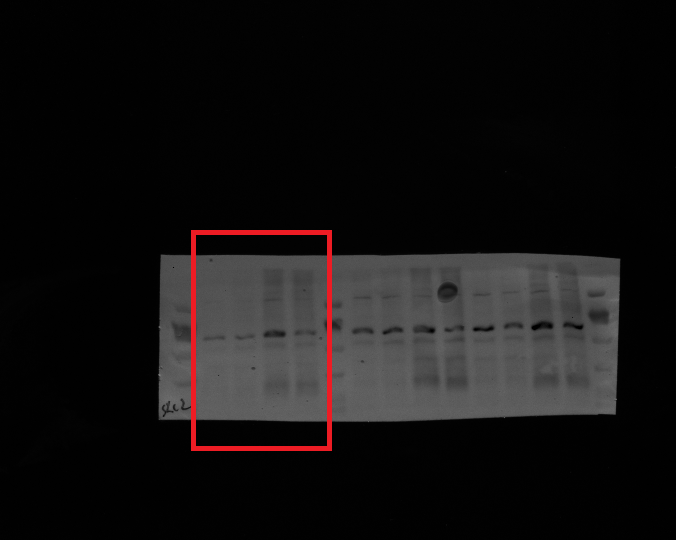

Supplement: S1 Raw images — (ZIP) [file pone.0322746.s004.zip › S1_RAW_images/fig 6 raw data/Fig 6 E lane 2-5SLC2B.tif]

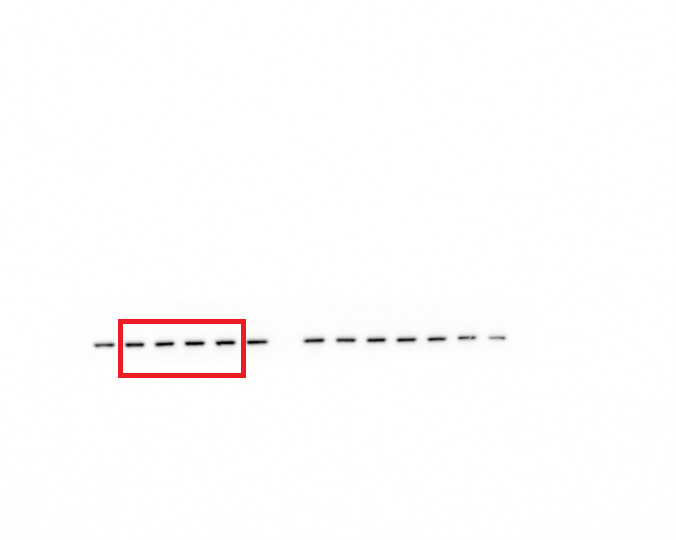

Supplement: S1 Raw images — (ZIP) [file pone.0322746.s004.zip › S1_RAW_images/fig 6 raw data/Fig 6 E β-Tubulin lane 2-5.tif]

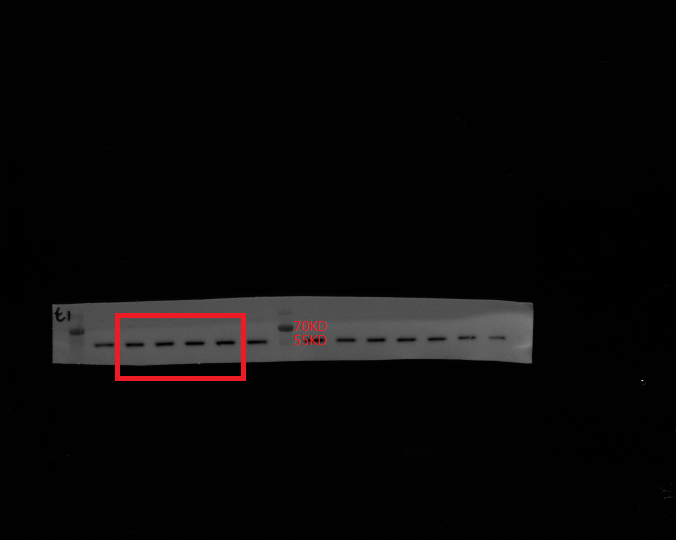

Supplement: S1 Raw images — (ZIP) [file pone.0322746.s004.zip › S1_RAW_images/fig 6 raw data/Fig 6 E β-Tubulin lane 2-5B.tif]

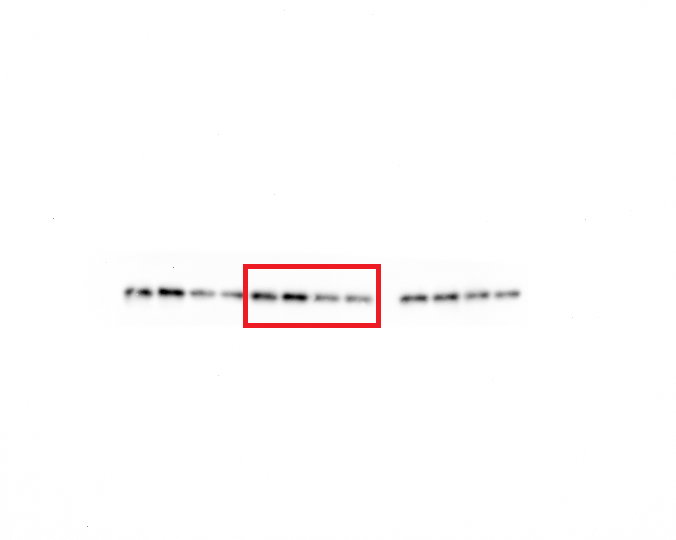

Supplement: S1 Raw images — (ZIP) [file pone.0322746.s004.zip › S1_RAW_images/fig 6 raw data/Fig 6 F GPX4 lane 6-9.tif]

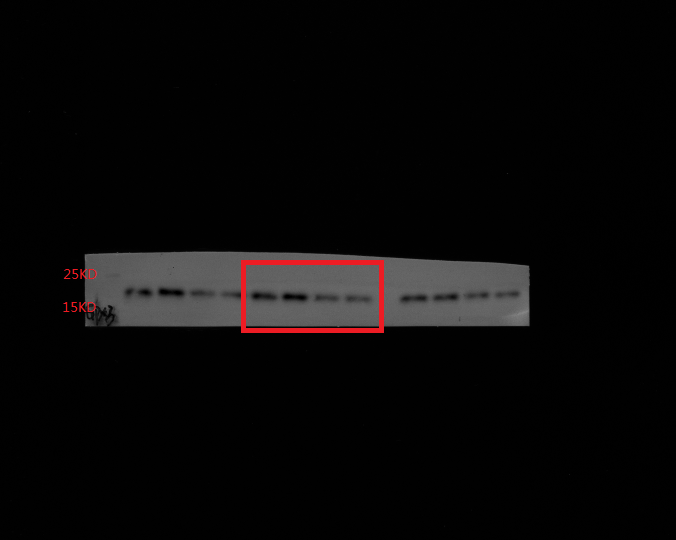

Supplement: S1 Raw images — (ZIP) [file pone.0322746.s004.zip › S1_RAW_images/fig 6 raw data/Fig 6 F GPX4 lane 6-9B.tif]

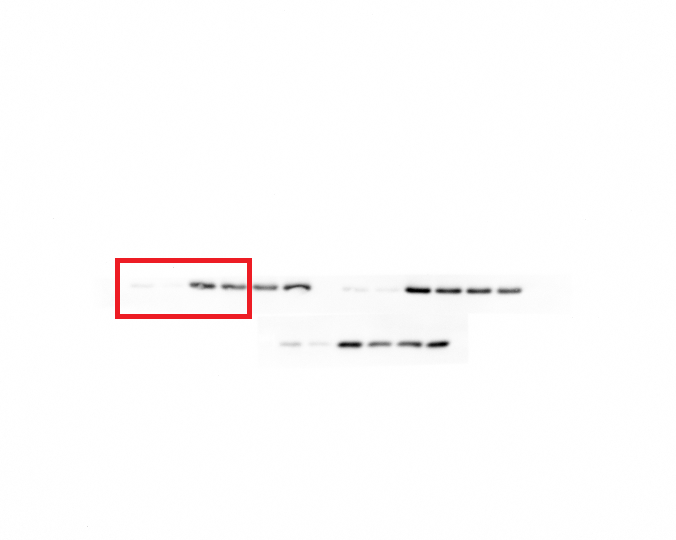

Supplement: S1 Raw images — (ZIP) [file pone.0322746.s004.zip › S1_RAW_images/fig 6 raw data/Fig 6 F HO-1 lane 2-5.tif]

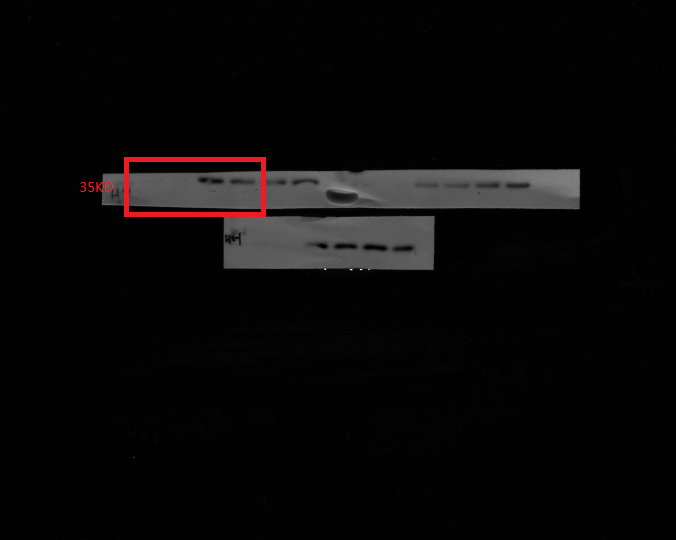

Supplement: S1 Raw images — (ZIP) [file pone.0322746.s004.zip › S1_RAW_images/fig 6 raw data/Fig 6 F HO-1 lane 2-5B.tif]

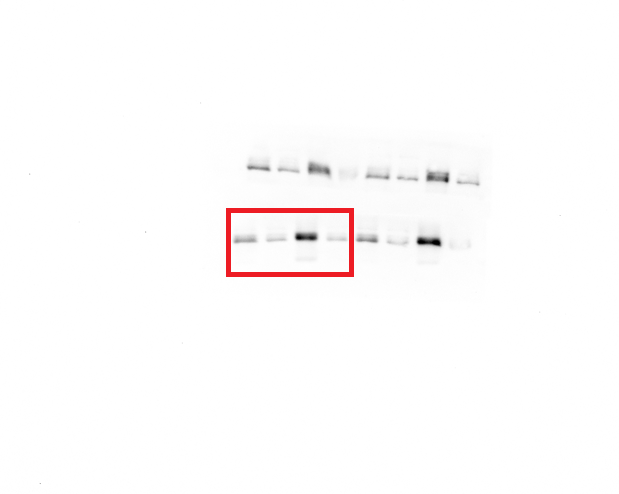

Supplement: S1 Raw images — (ZIP) [file pone.0322746.s004.zip › S1_RAW_images/fig 6 raw data/Fig 6 F NRF2 lane 2-5.tif]

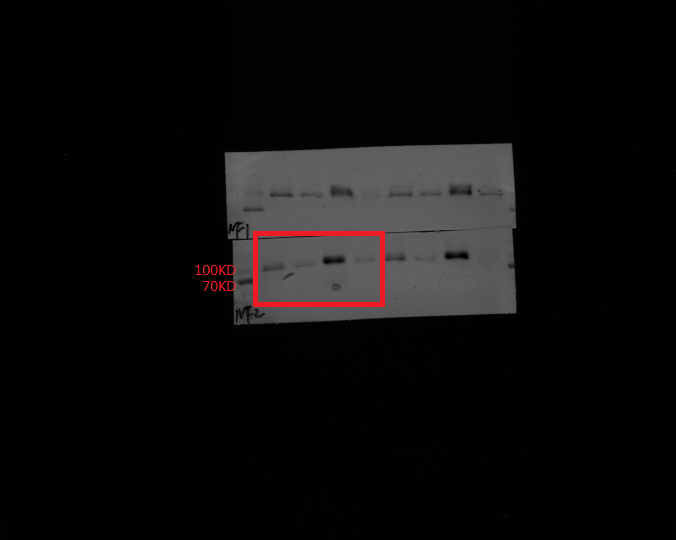

Supplement: S1 Raw images — (ZIP) [file pone.0322746.s004.zip › S1_RAW_images/fig 6 raw data/Fig 6 F NRF2 lane 2-5B.tif]

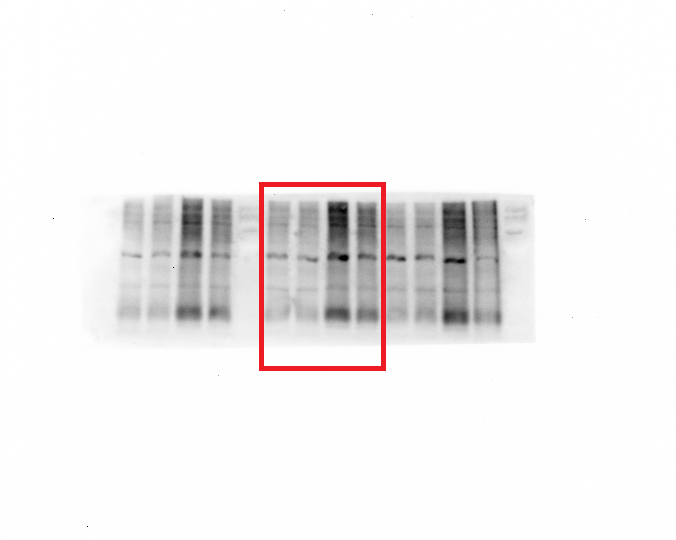

Supplement: S1 Raw images — (ZIP) [file pone.0322746.s004.zip › S1_RAW_images/fig 6 raw data/Fig 6 F SLC7A11 lane 6-9.tif]

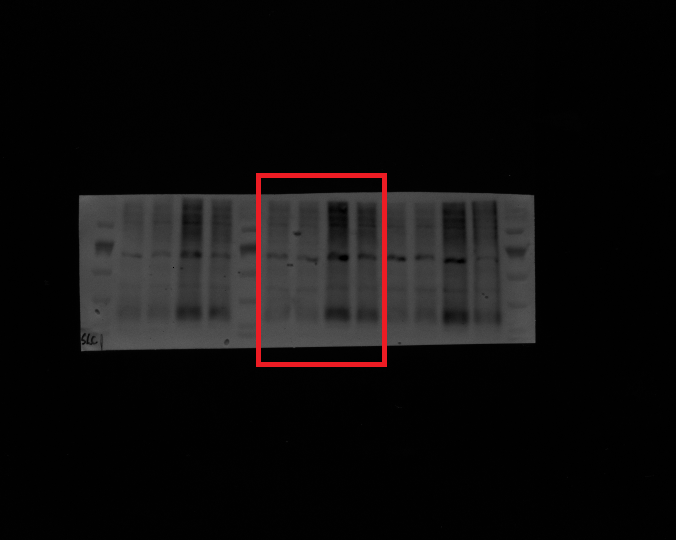

Supplement: S1 Raw images — (ZIP) [file pone.0322746.s004.zip › S1_RAW_images/fig 6 raw data/Fig 6 F SLC7A11 lane 6-9B.tif]

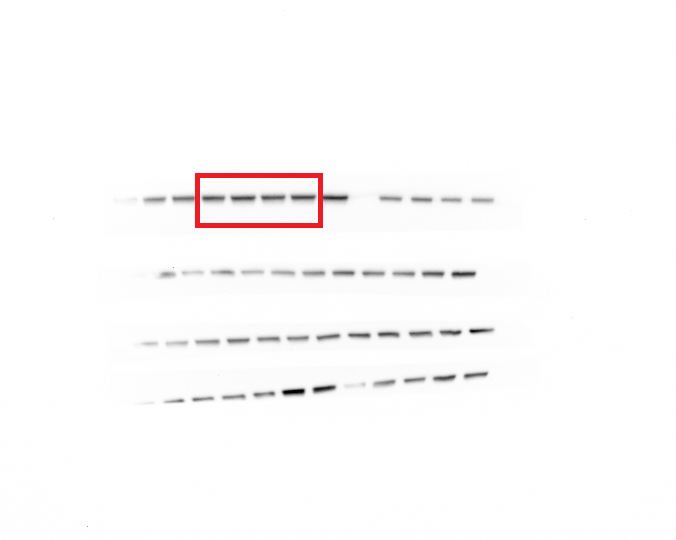

Supplement: S1 Raw images — (ZIP) [file pone.0322746.s004.zip › S1_RAW_images/fig 6 raw data/Fig 6 F β-Tubulin lane 4-7.tif]

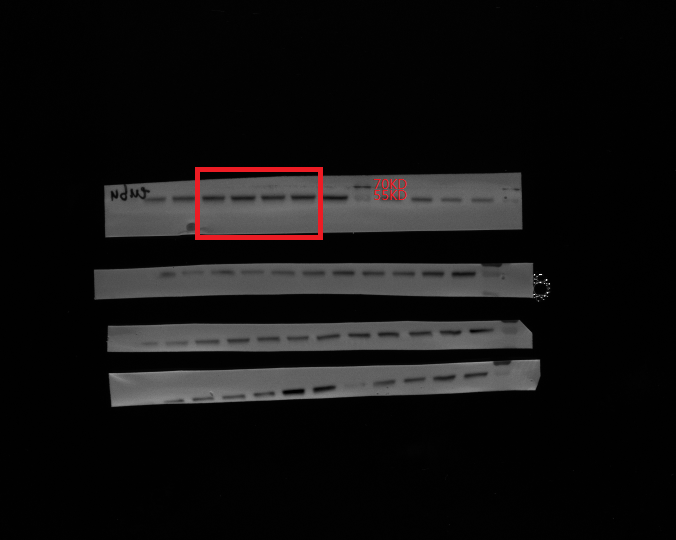

Supplement: S1 Raw images — (ZIP) [file pone.0322746.s004.zip › S1_RAW_images/fig 6 raw data/Fig 6 F β-Tubulin lane 4-7B.tif]

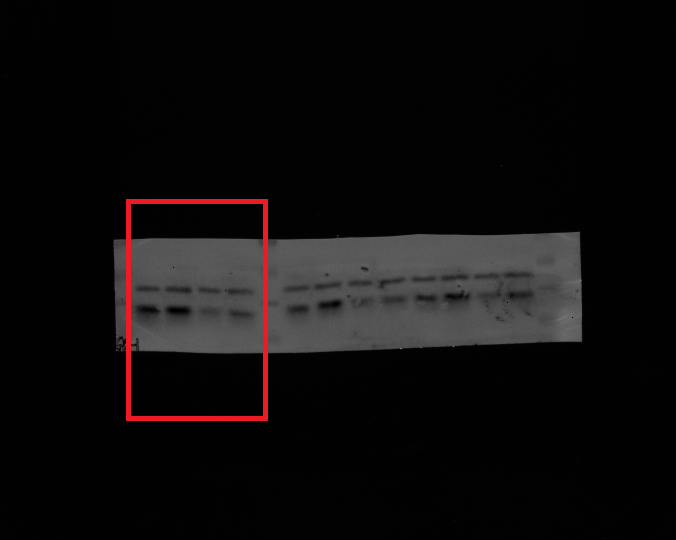

Supplement: S1 Raw images — (ZIP) [file pone.0322746.s004.zip › S1_RAW_images/figs 3 raw data/FIGS 3A GPX4 lane 2-5 B.tif]

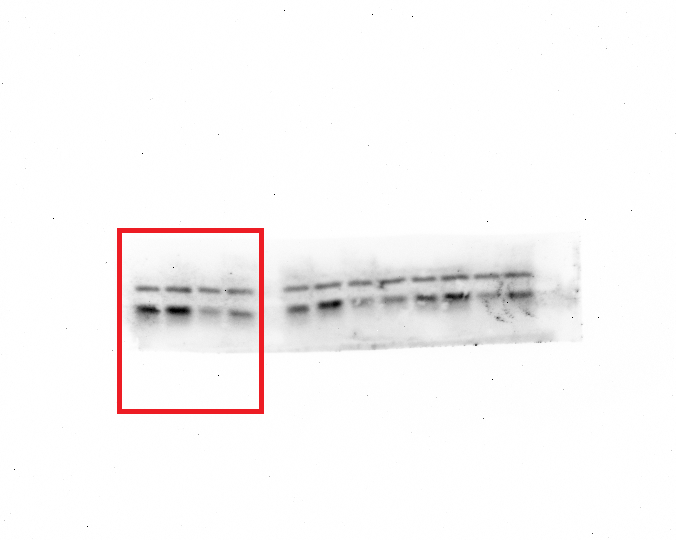

Supplement: S1 Raw images — (ZIP) [file pone.0322746.s004.zip › S1_RAW_images/figs 3 raw data/FIGS 3A GPX4 lane2-5.tif]

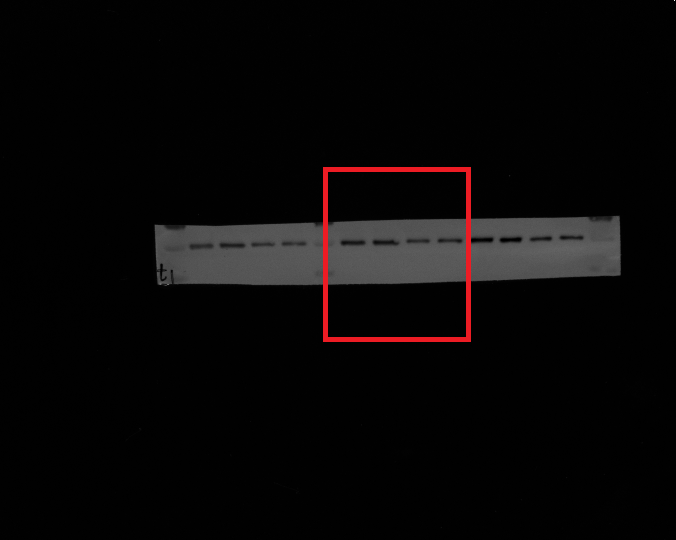

Supplement: S1 Raw images — (ZIP) [file pone.0322746.s004.zip › S1_RAW_images/figs 3 raw data/FIGS 3A TUBIN11 lane7-10 B.tif]

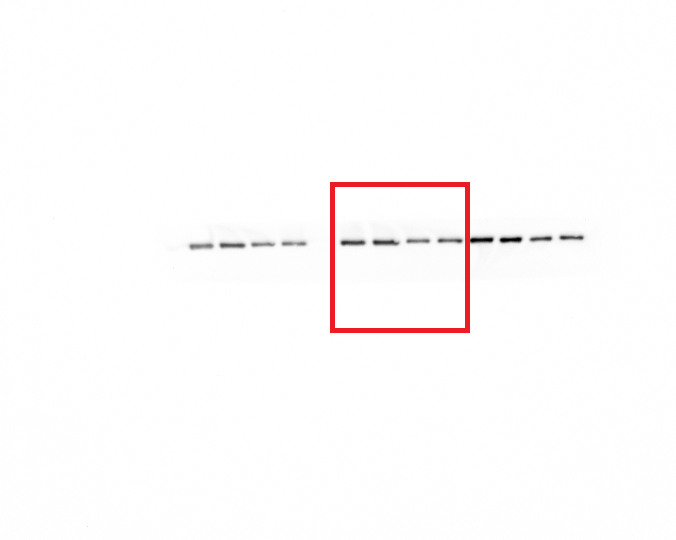

Supplement: S1 Raw images — (ZIP) [file pone.0322746.s004.zip › S1_RAW_images/figs 3 raw data/FIGS 3A TUBIN11 lane7-10.tif]

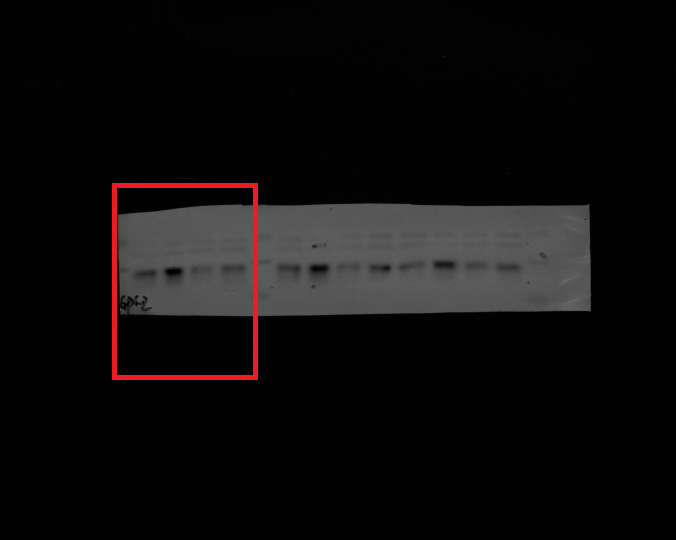

Supplement: S1 Raw images — (ZIP) [file pone.0322746.s004.zip › S1_RAW_images/figs 3 raw data/FIGS 3B GPX4 lane2-5 B.tif]

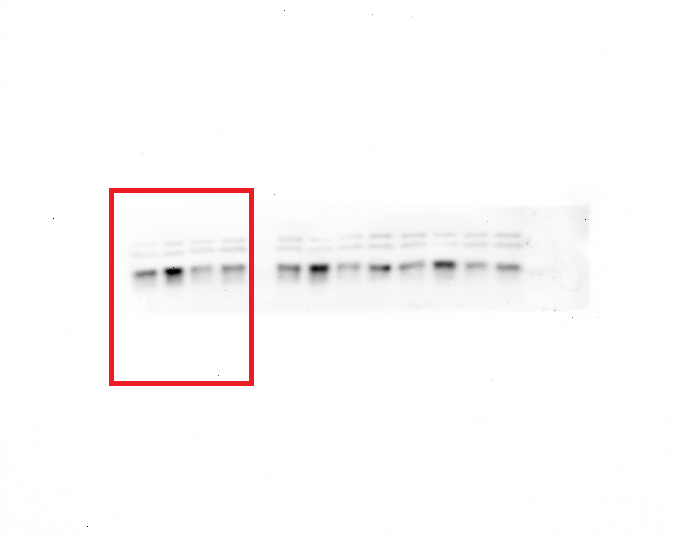

Supplement: S1 Raw images — (ZIP) [file pone.0322746.s004.zip › S1_RAW_images/figs 3 raw data/FIGS 3B GPX4 lane2-5.tif]

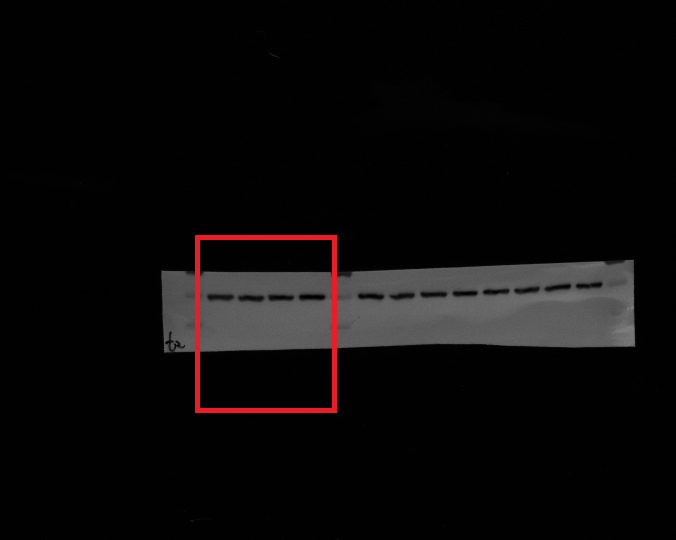

Supplement: S1 Raw images — (ZIP) [file pone.0322746.s004.zip › S1_RAW_images/figs 3 raw data/FIGS 3B TUBLIN2 lane2-5 B.tif]

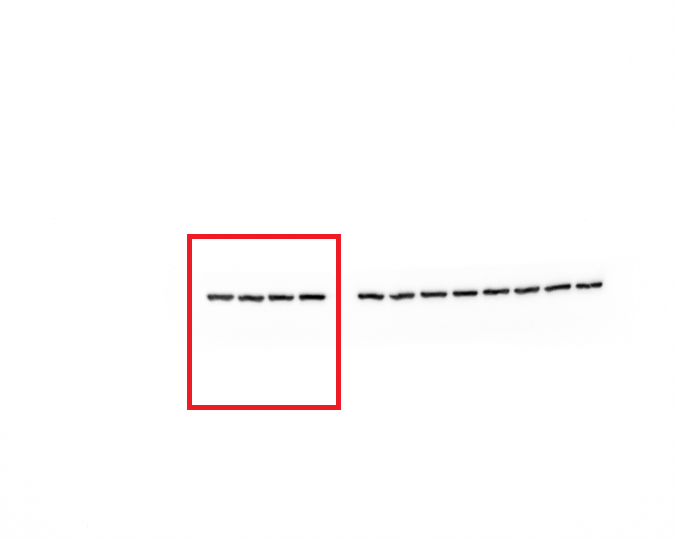

Supplement: S1 Raw images — (ZIP) [file pone.0322746.s004.zip › S1_RAW_images/figs 3 raw data/FIGS 3B TUBLIN2 lane2-5.tif]
