# Supplementary material for: Silencing NRF2 enhances arsenic trioxide-induced ferroptosis in hepatocellular carcinoma cells
Source: PLoS One. 2025 May 22;20(5):e0322746. doi: 10.1371/journal.pone.0322746 (PMC12097587; doi:10.1371/journal.pone.0322746)
Supplement: S1 Raw images — (ZIP) [file pone.0322746.s004.zip › S1_RAW_images/Fig 4 raw data.pdf]

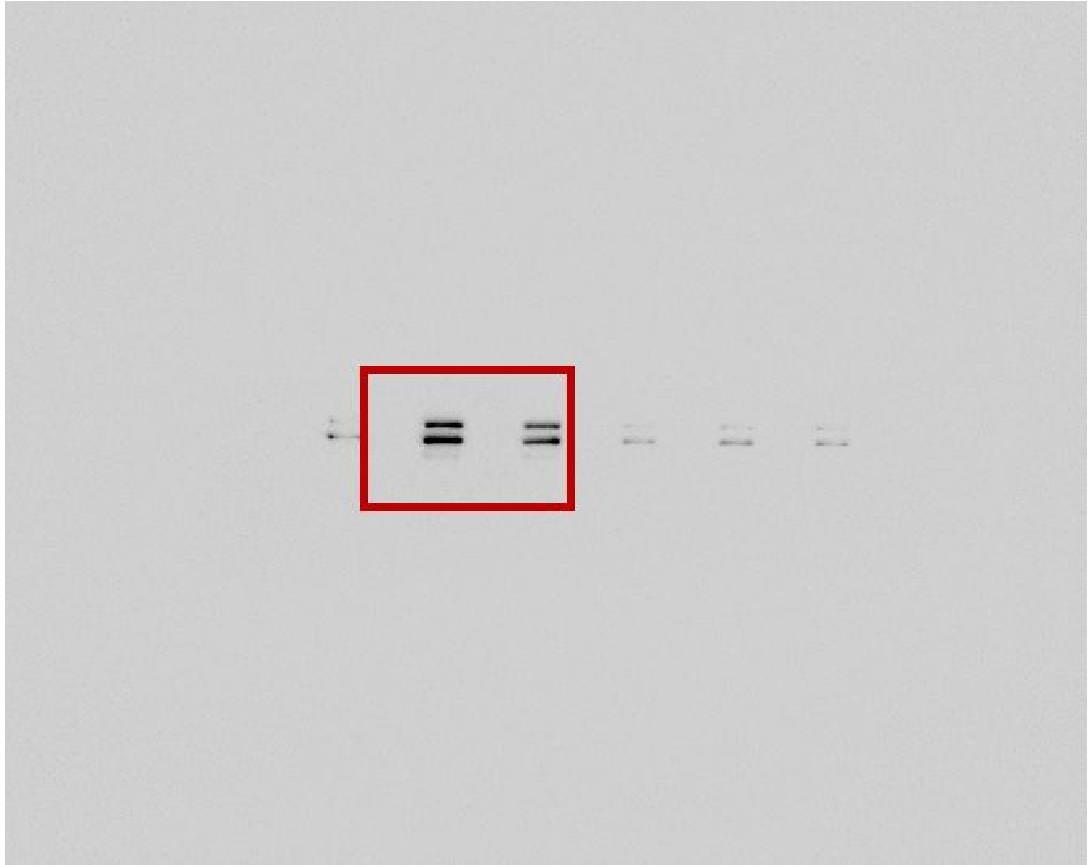

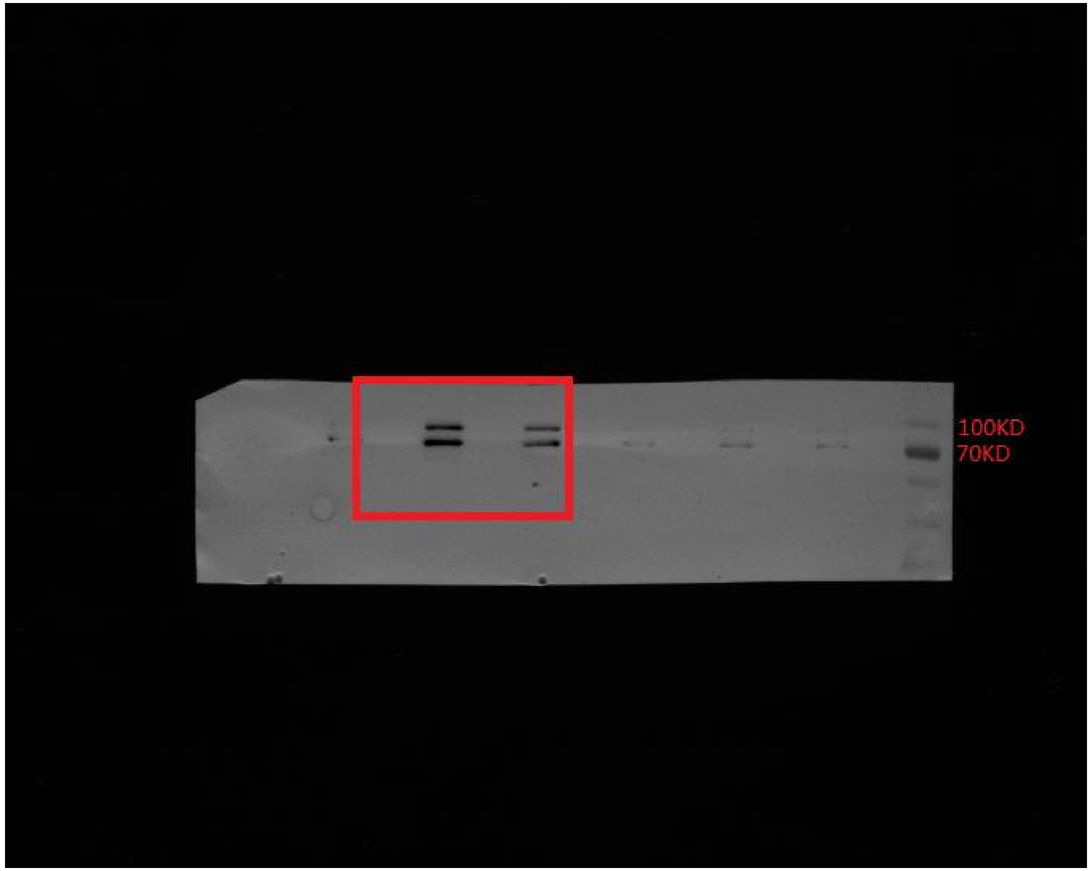

Fig 4 c LaminA lane 3-6

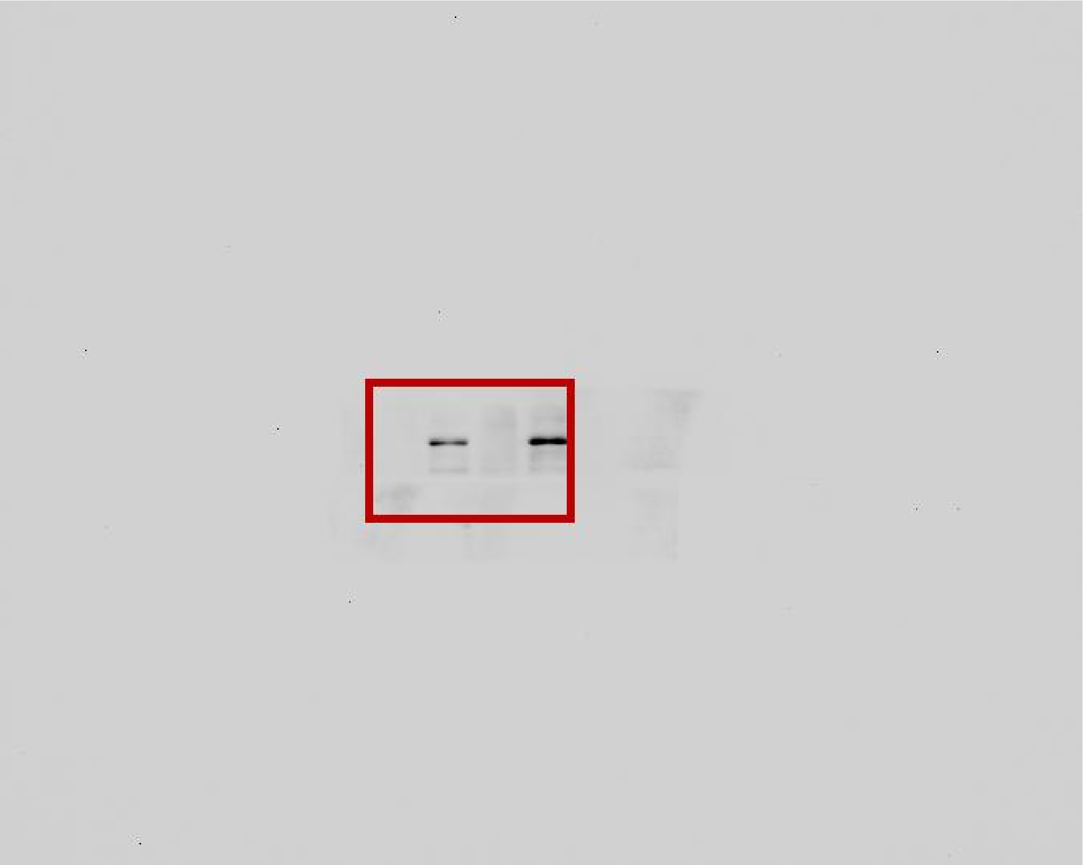

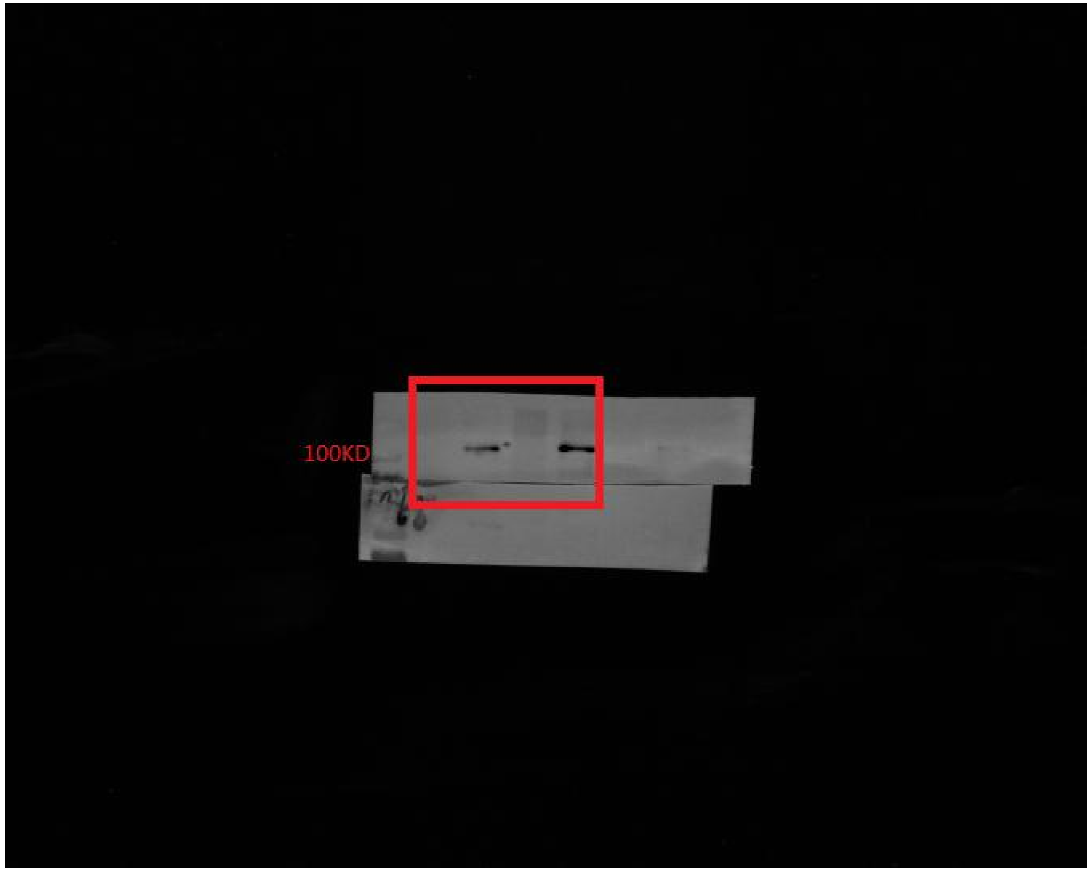

Fig 4 c NRF2 lane 2-5

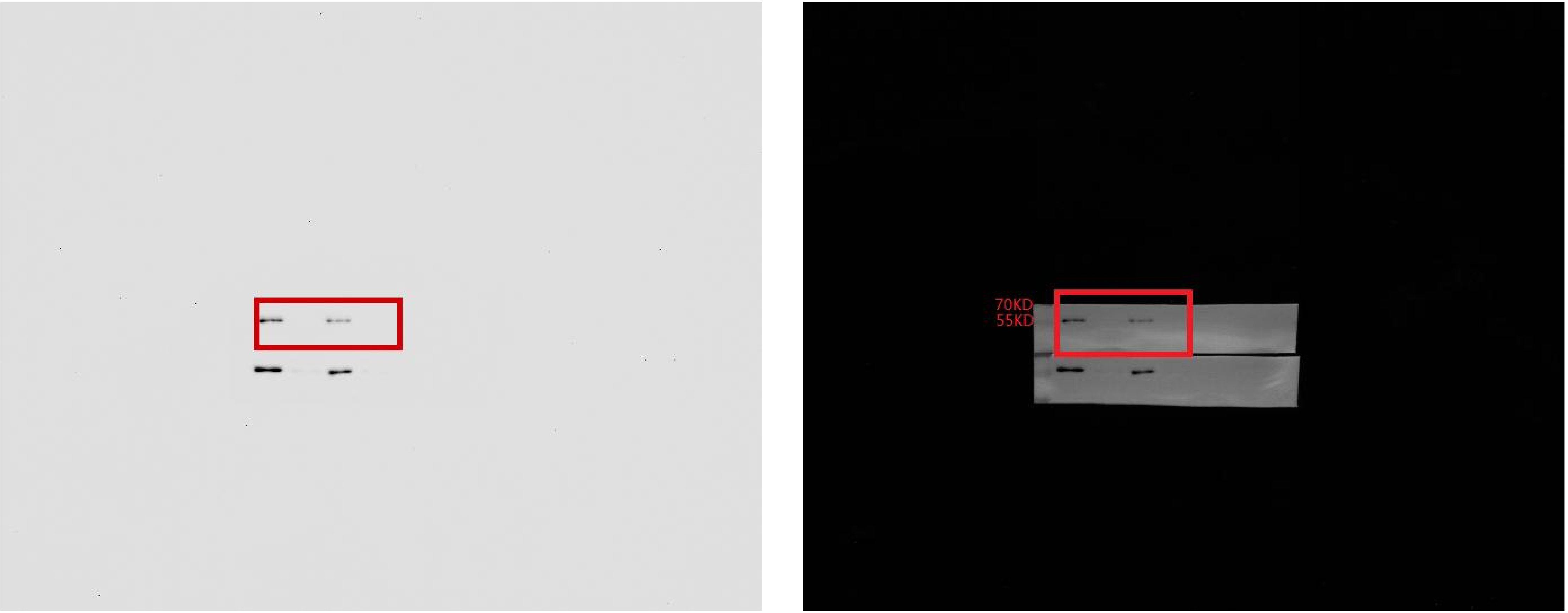

Fig 4 c β-Tubulin lane 2-5

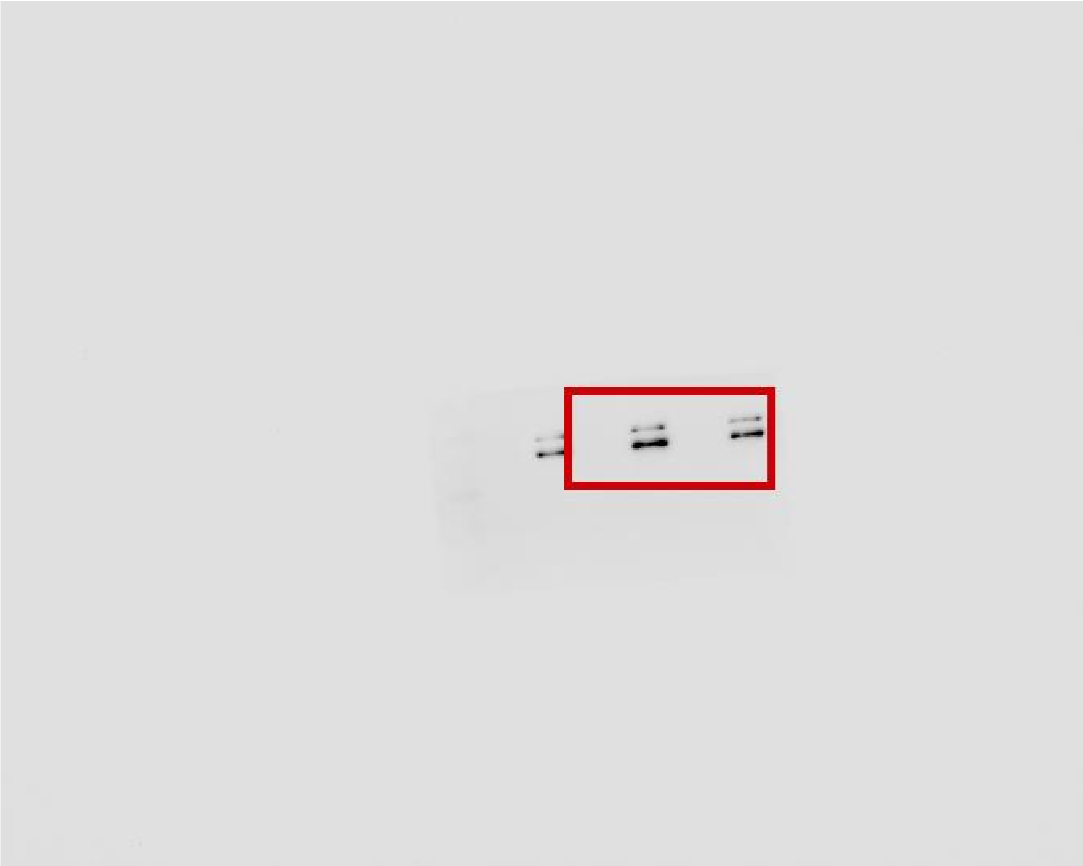

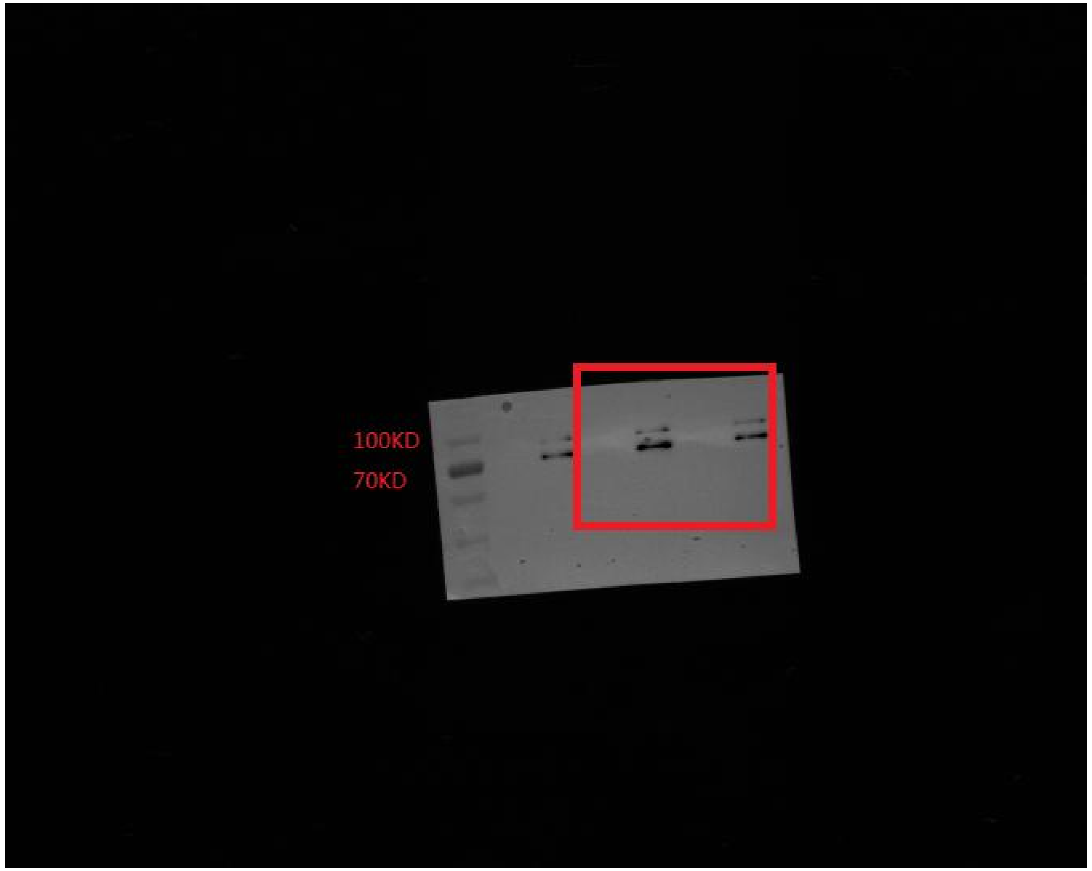

Fig 4 d LaminA lane 4-7

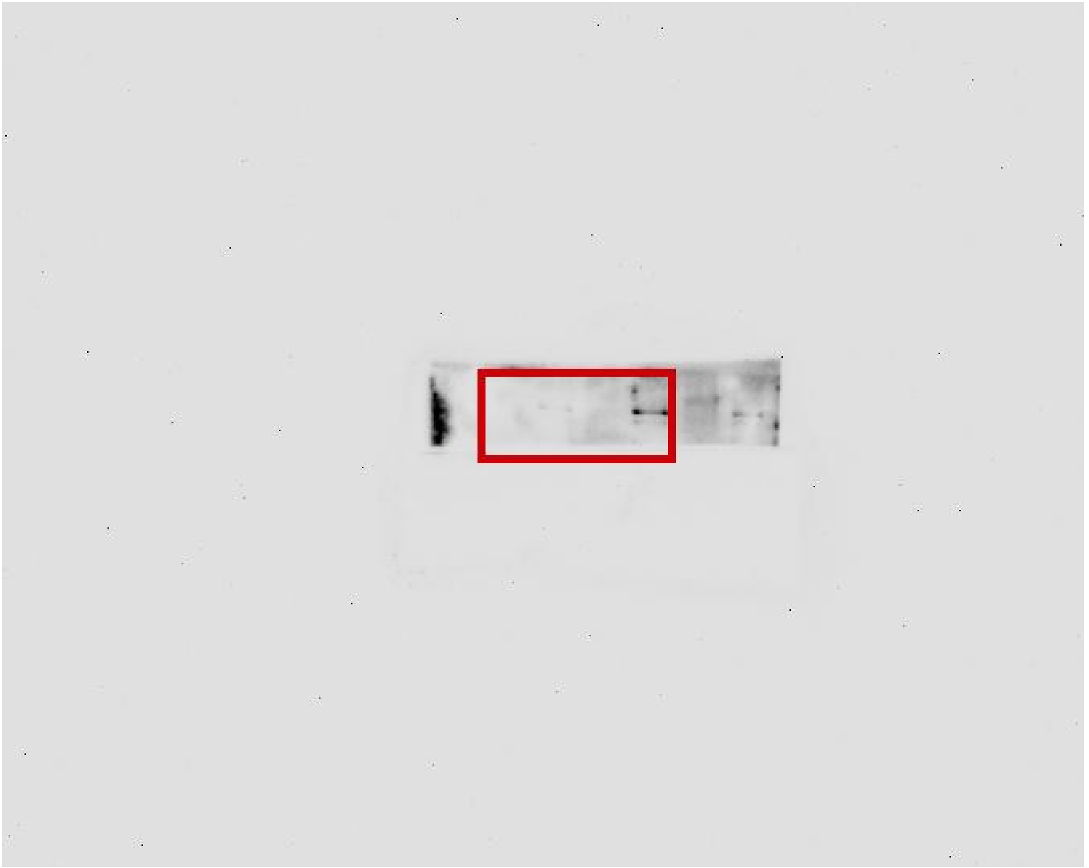

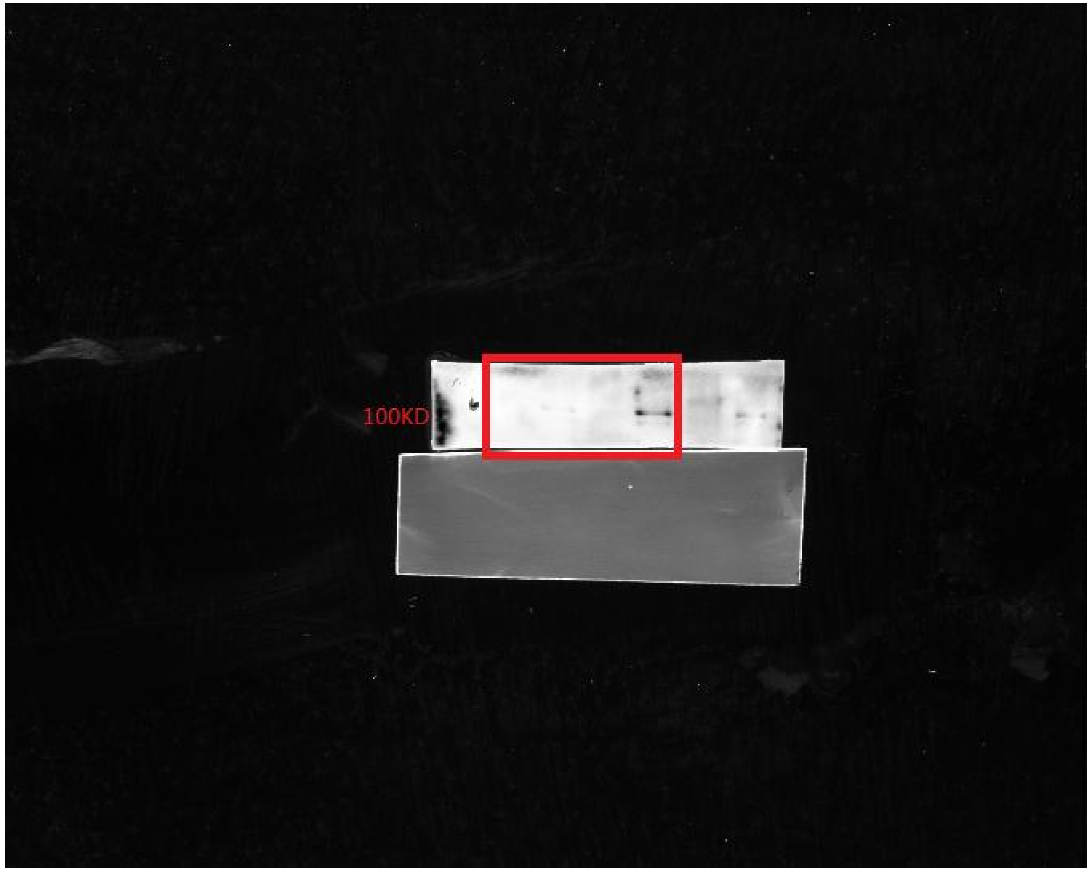

Fig 4 d NRF2 lane 2-5

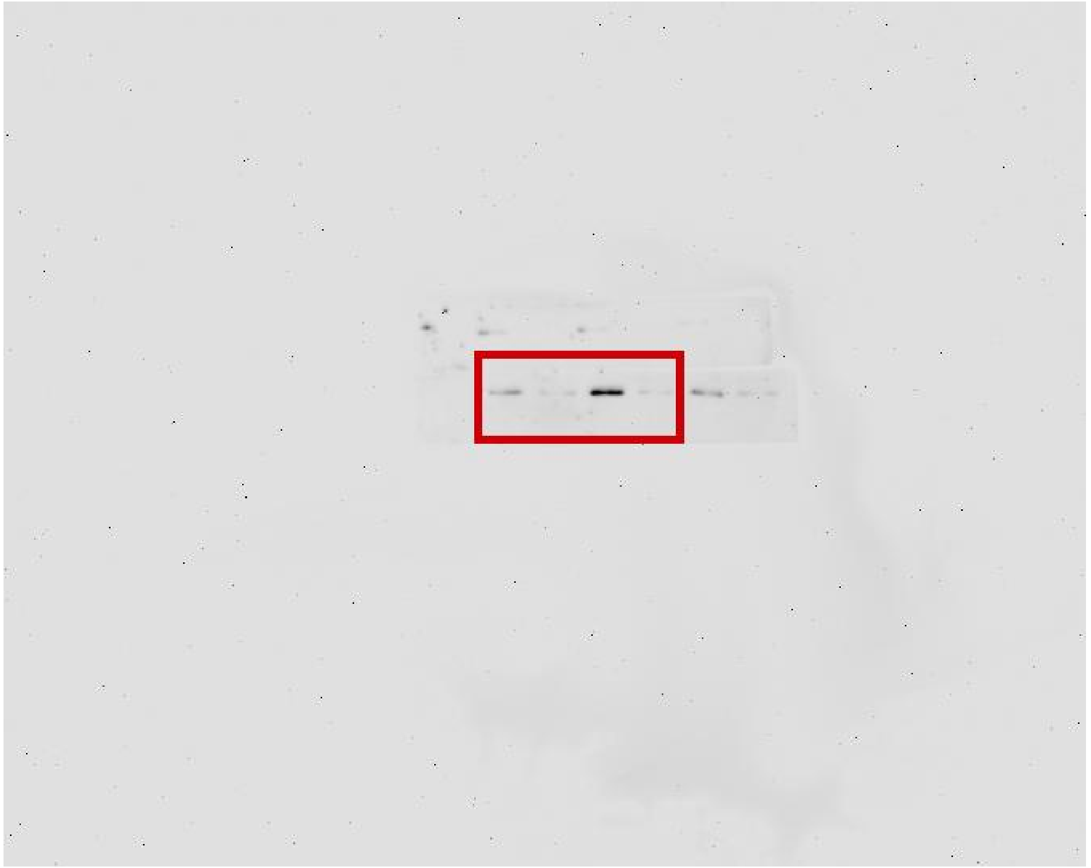

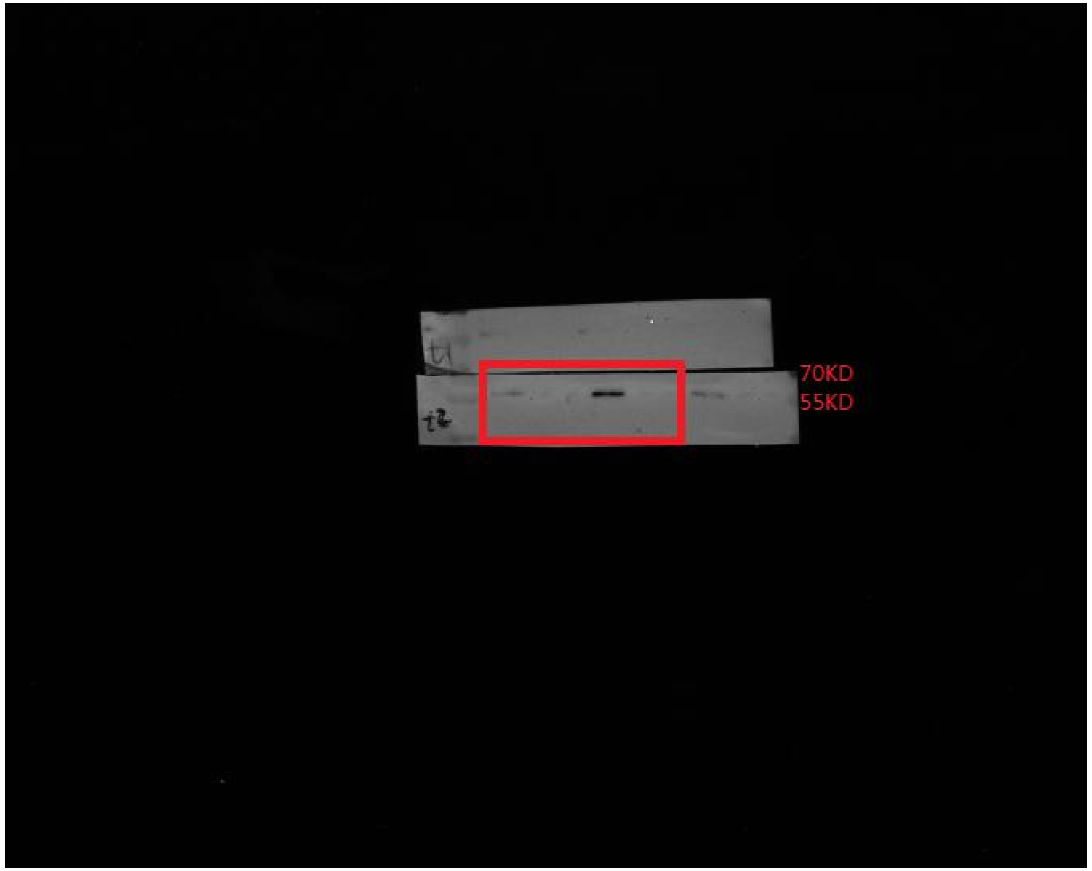

Fig 4 d β-Tubulin lane 2-5

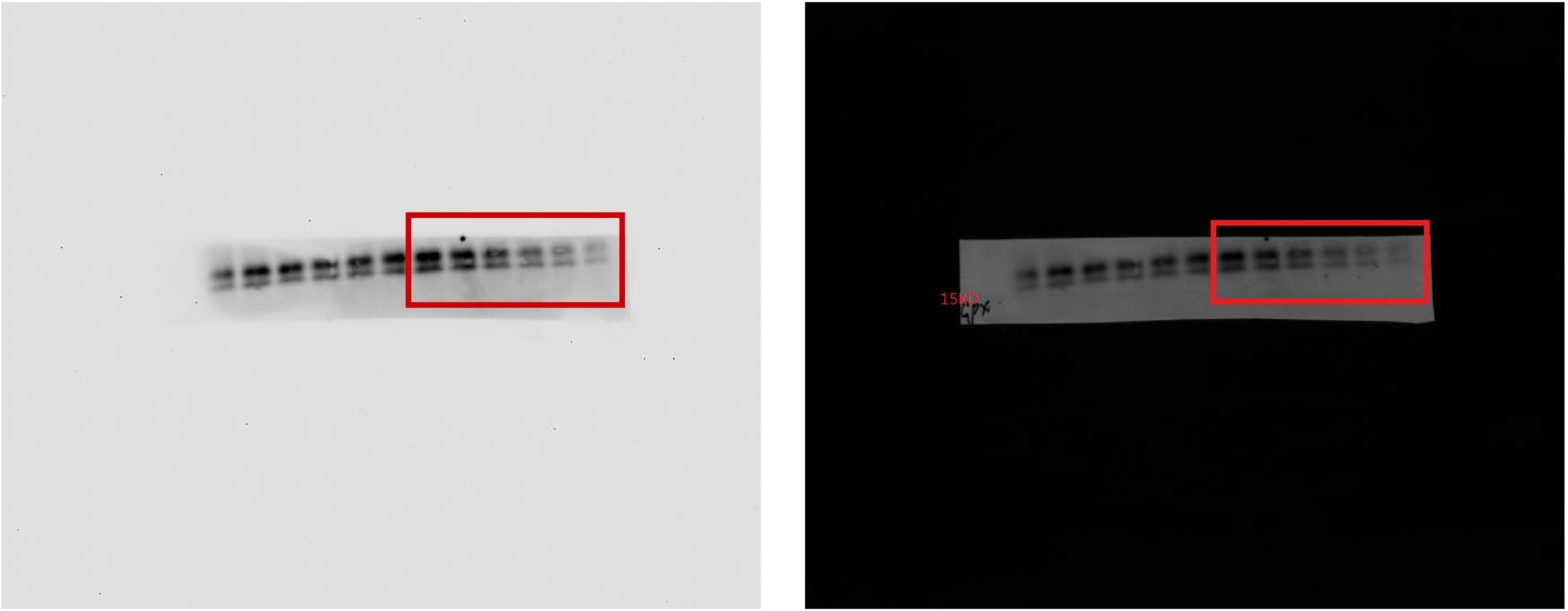

Fig 4 e GPX4 lane 8-13

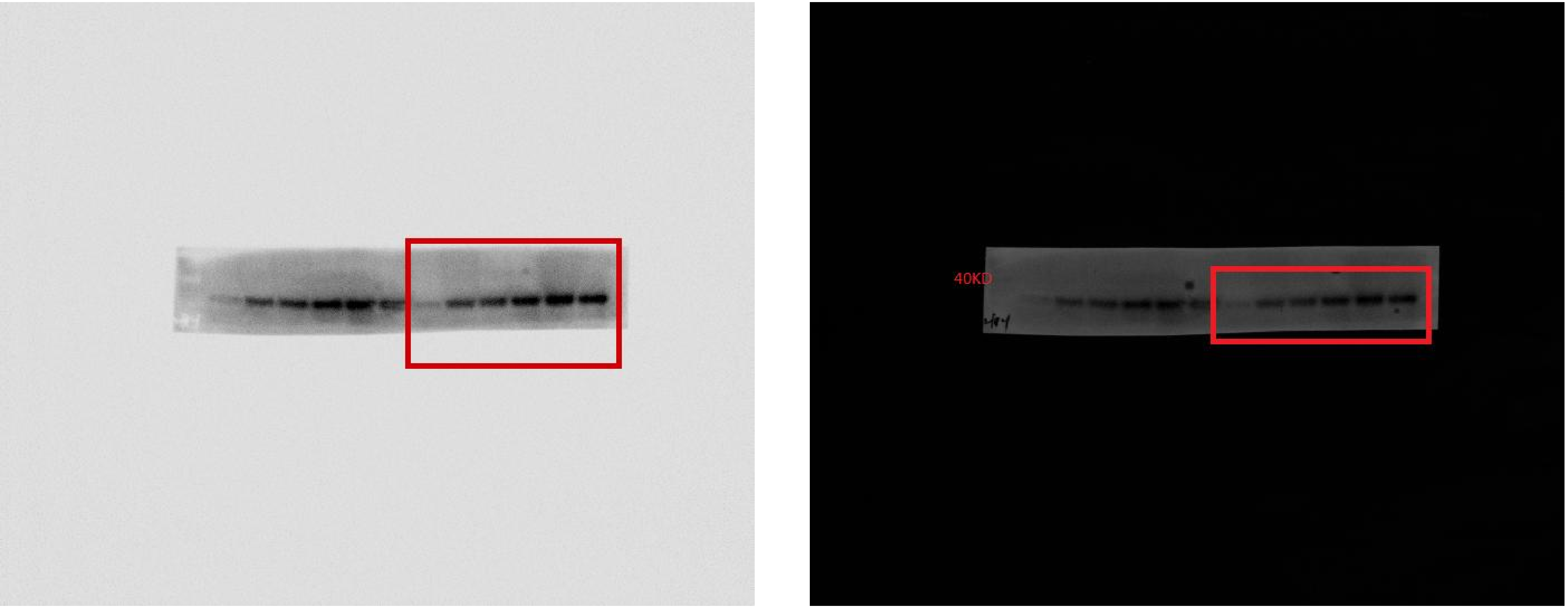

Fig 4 e HO-1 lane 8-13

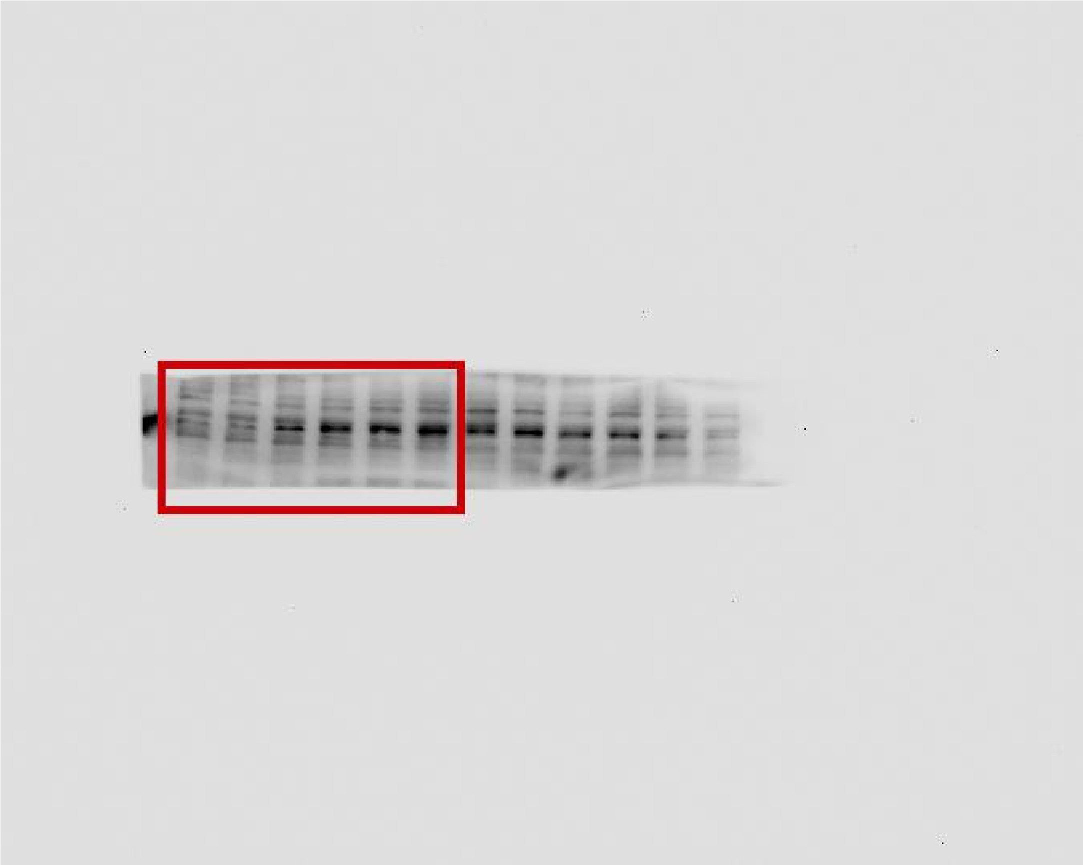

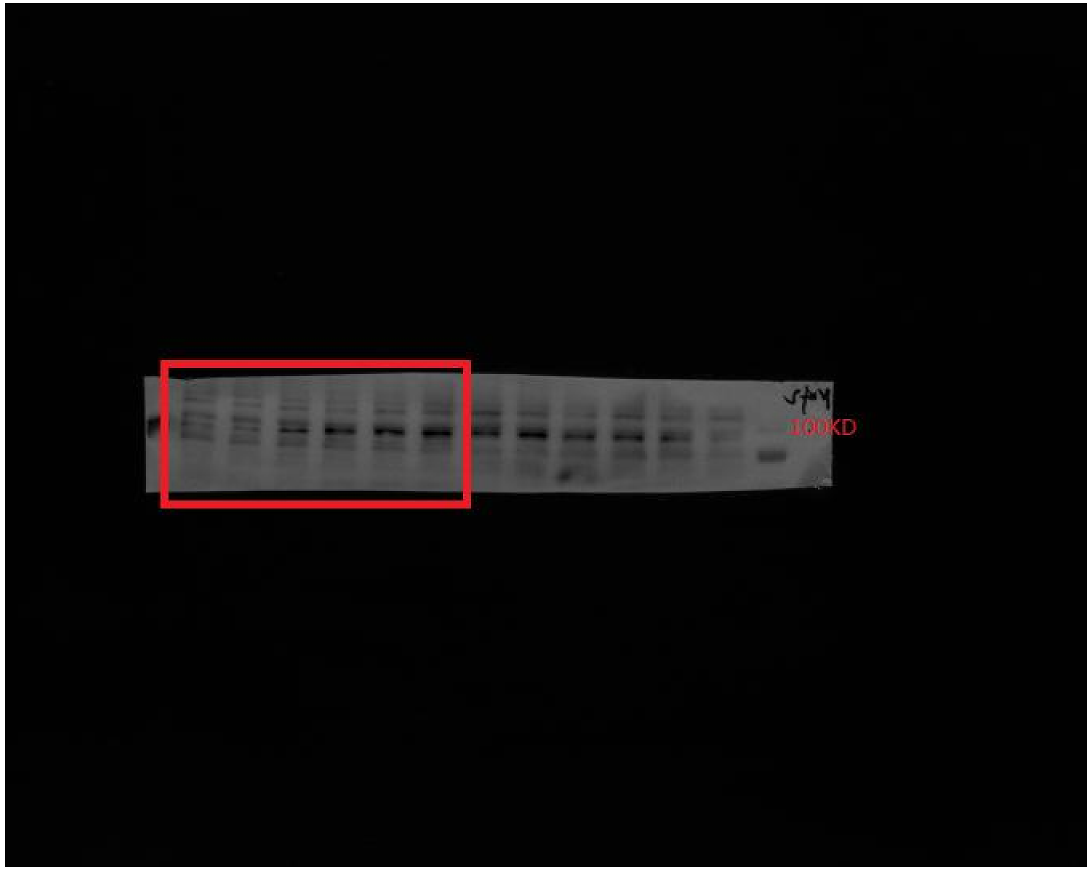

Fig 4 e NRF2 lane 2-7

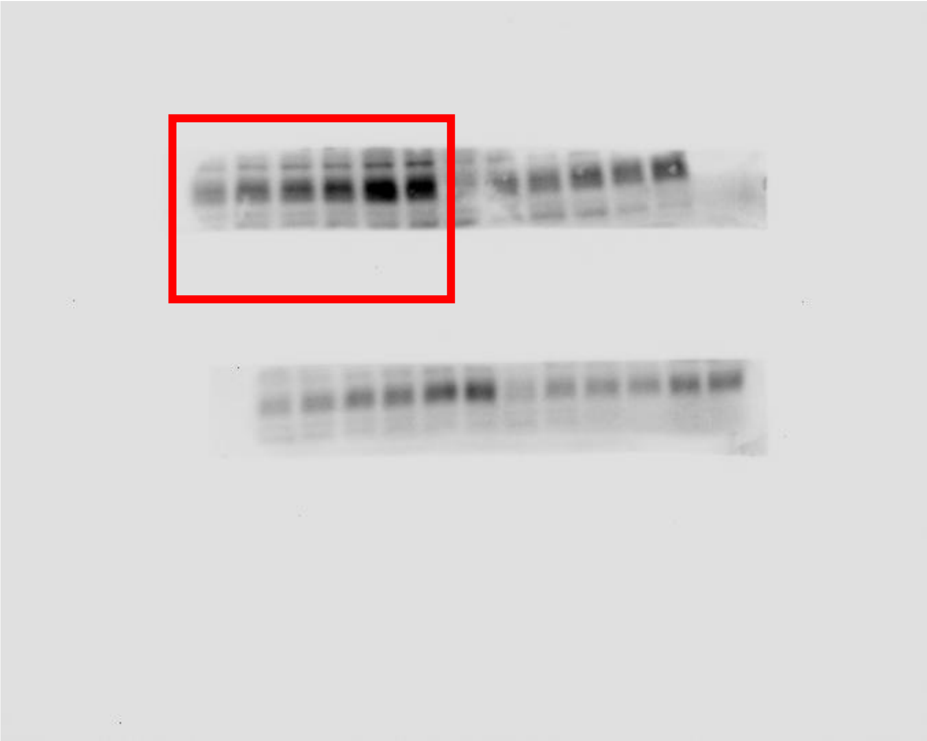

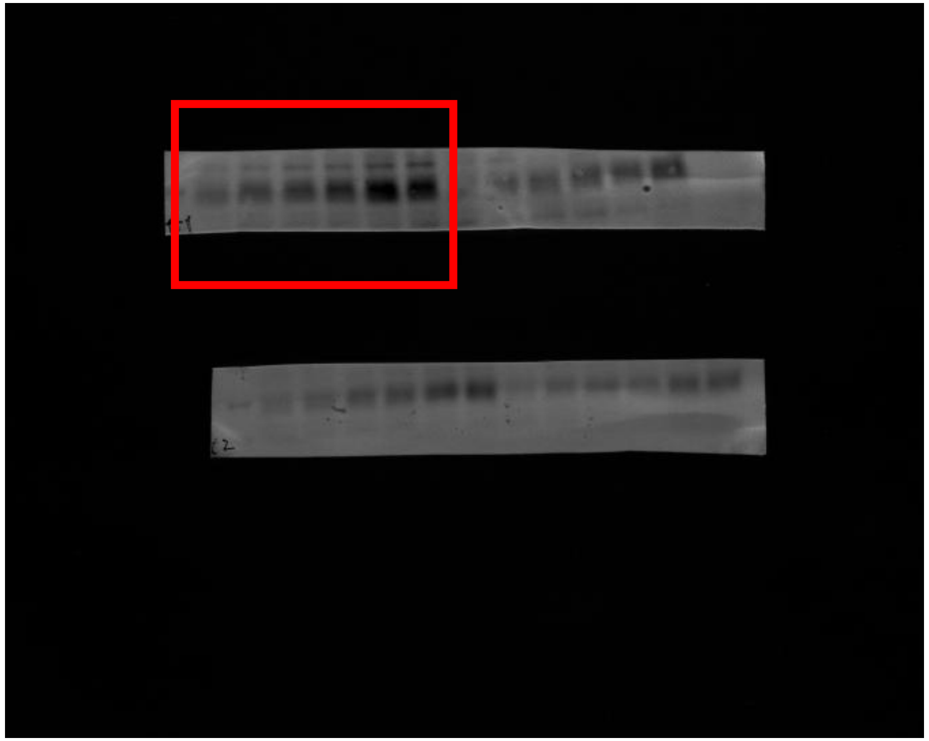

Fig 4 e SLC7A11 lane 2-7

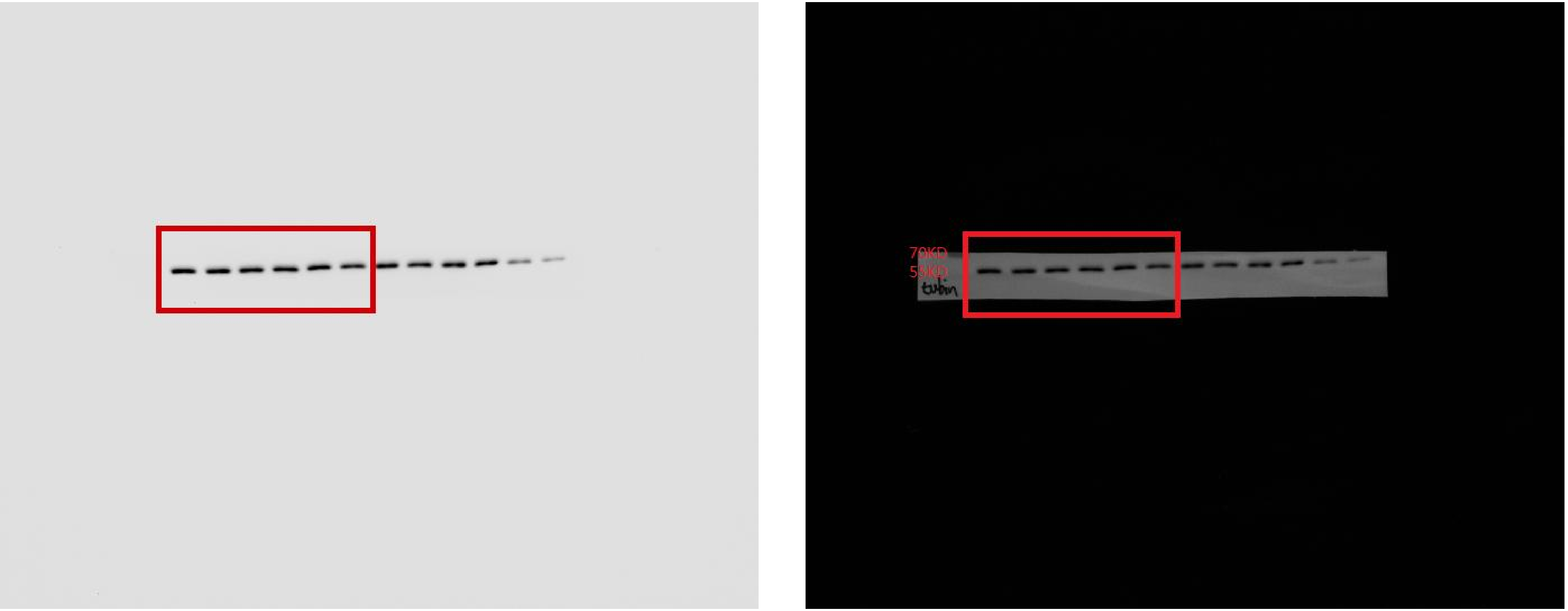

Fig 4 e β-Tubulin lane 2-7

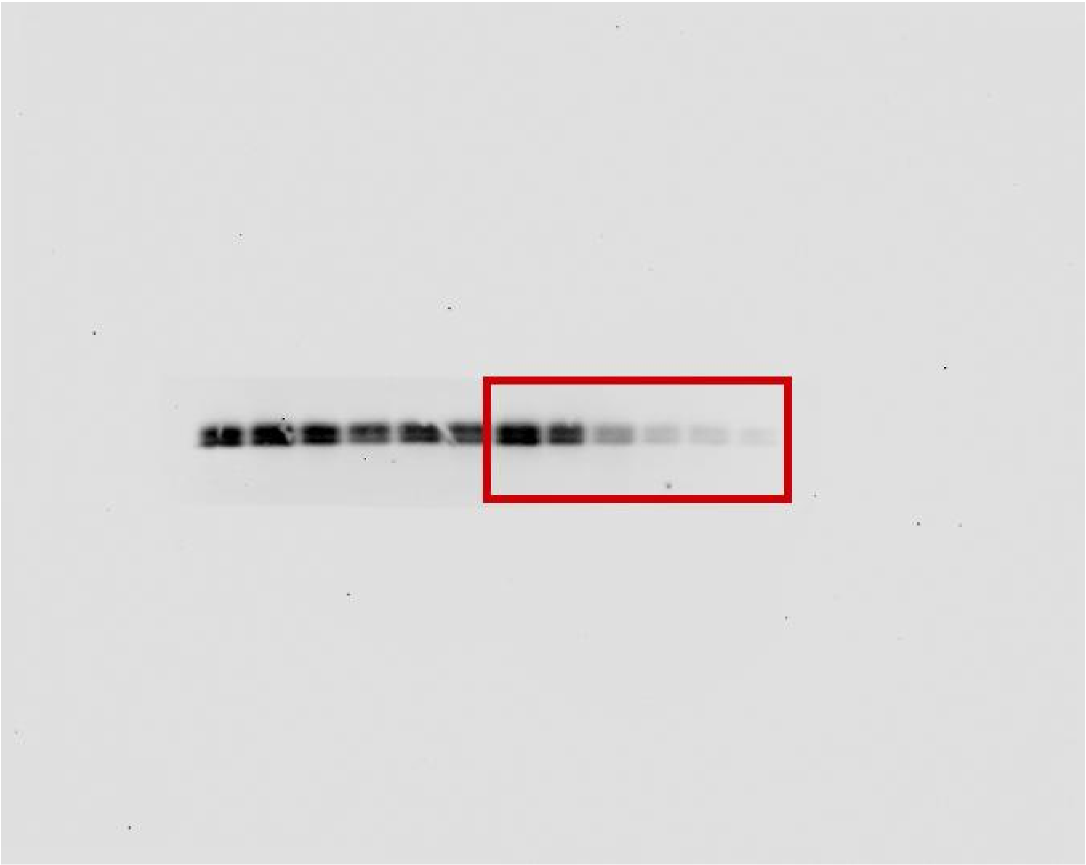

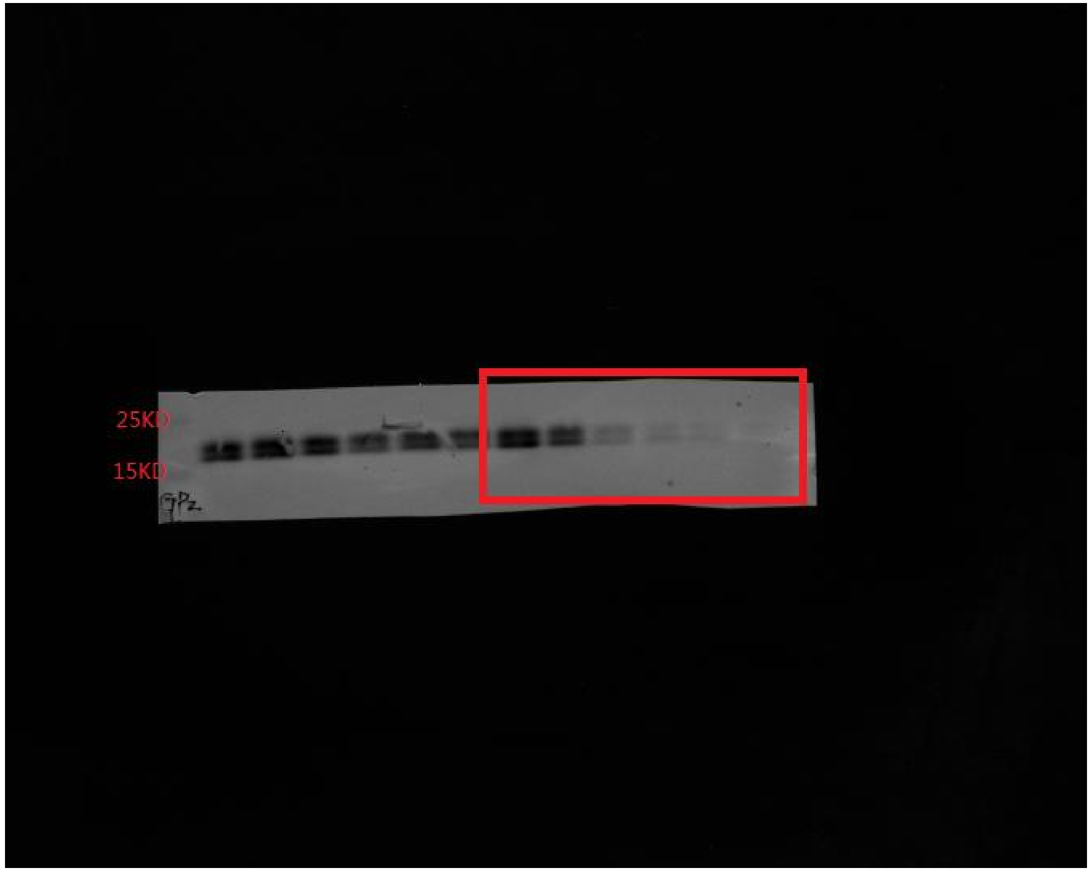

Fig 4 f GPX4 lane 8-13

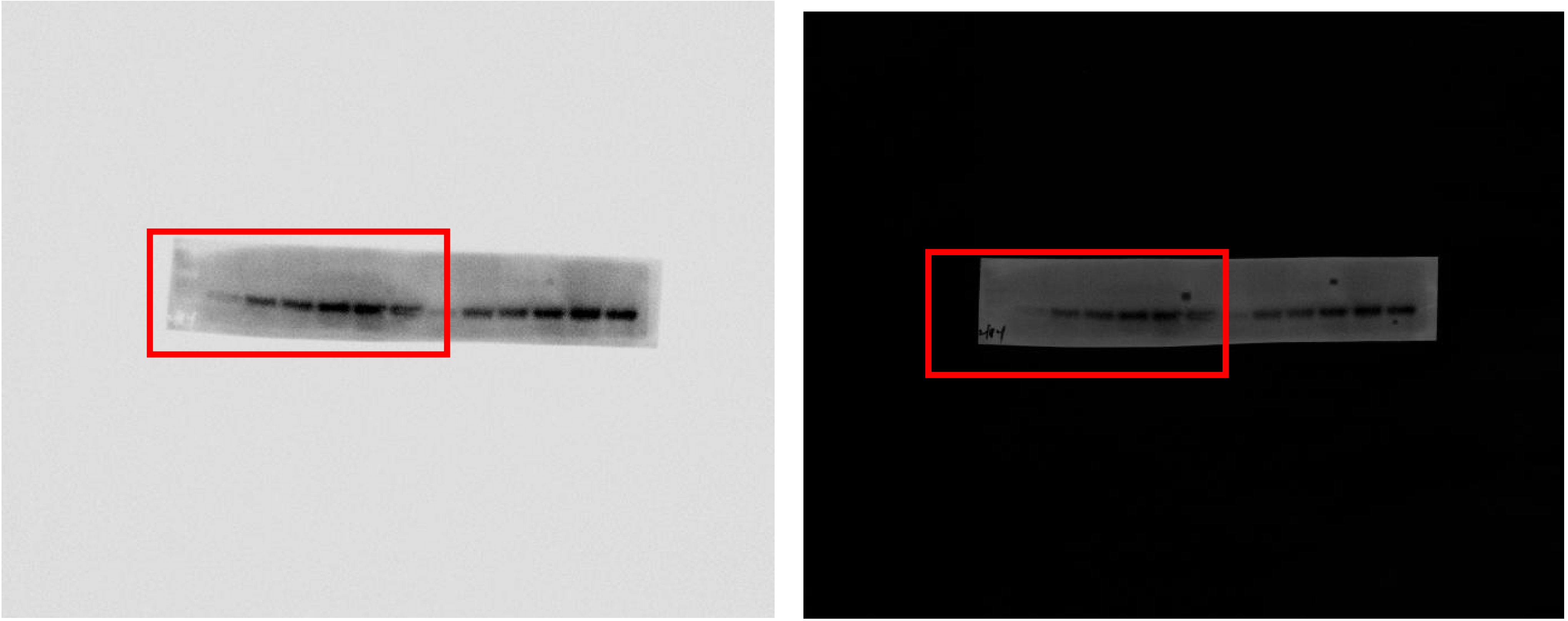

Fig 4 f HO-1 lane 2-7

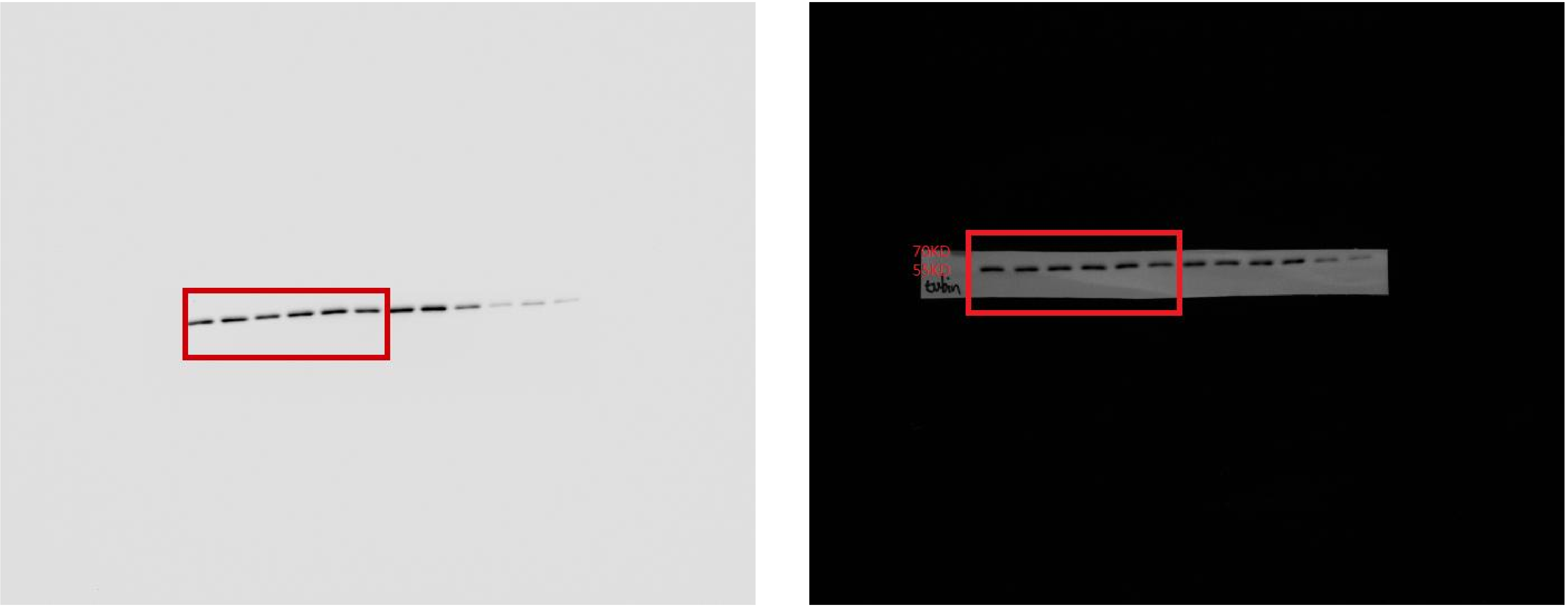

Fig 4 f β-Tubulin lane 2-7

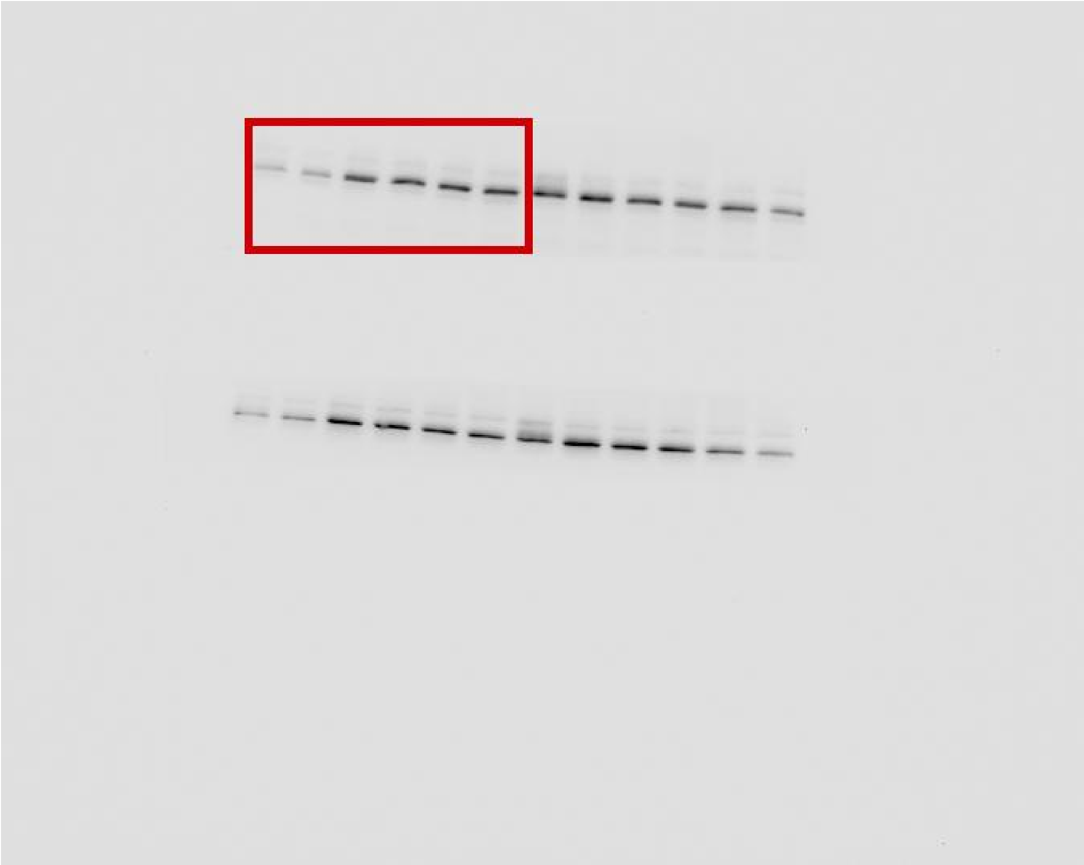

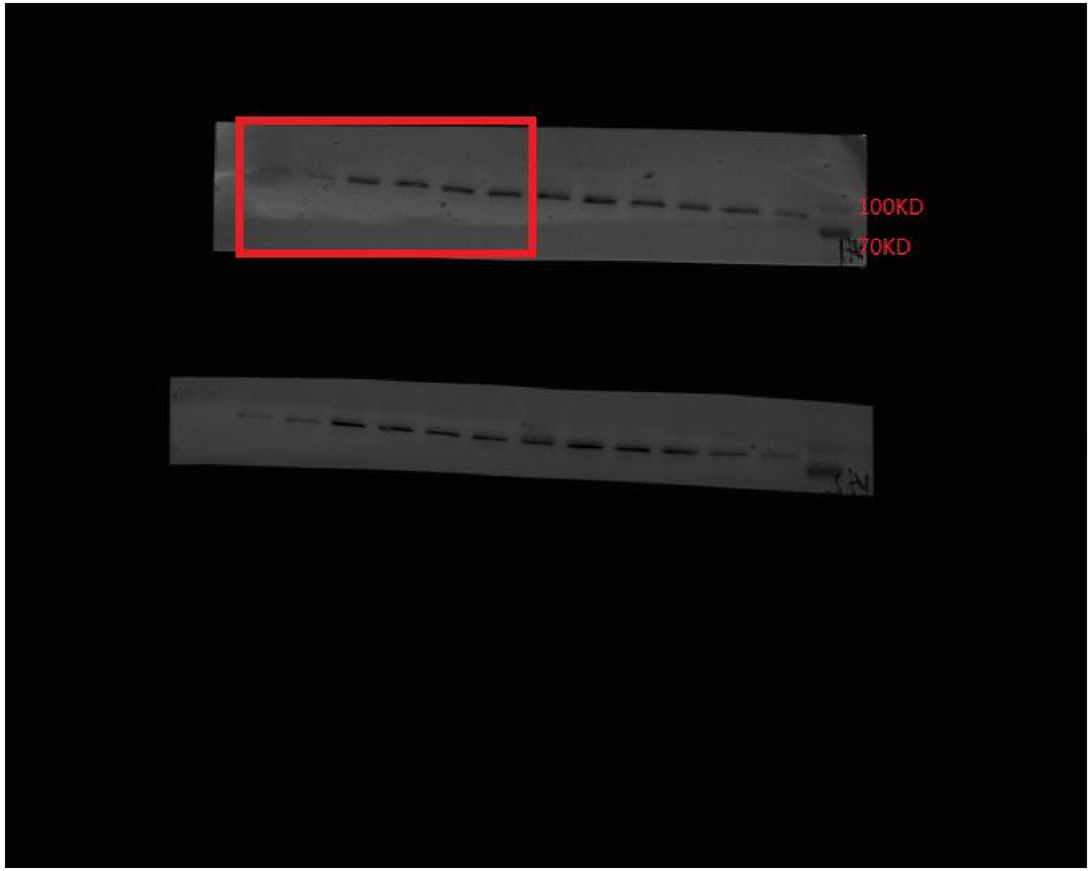

Fig 4 f NRF2 lane 2-7

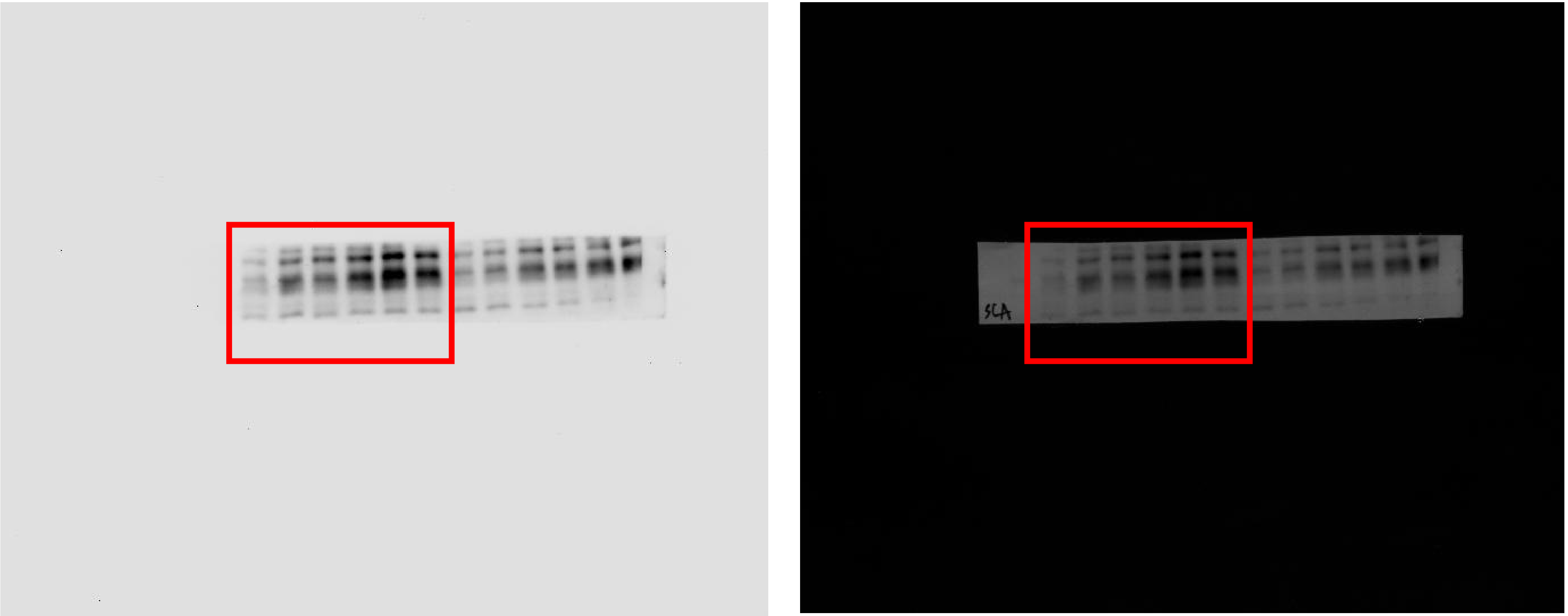

Fig 4 f SLC7A11 lane 2-7
