# Supplementary material for: Silencing NRF2 enhances arsenic trioxide-induced ferroptosis in hepatocellular carcinoma cells
Source: PLoS One. 2025 May 22;20(5):e0322746. doi: 10.1371/journal.pone.0322746 (PMC12097587; doi:10.1371/journal.pone.0322746)
Supplement: S1 Raw images — (ZIP) [file pone.0322746.s004.zip › S1_RAW_images/Fig 6 raw data.pdf]

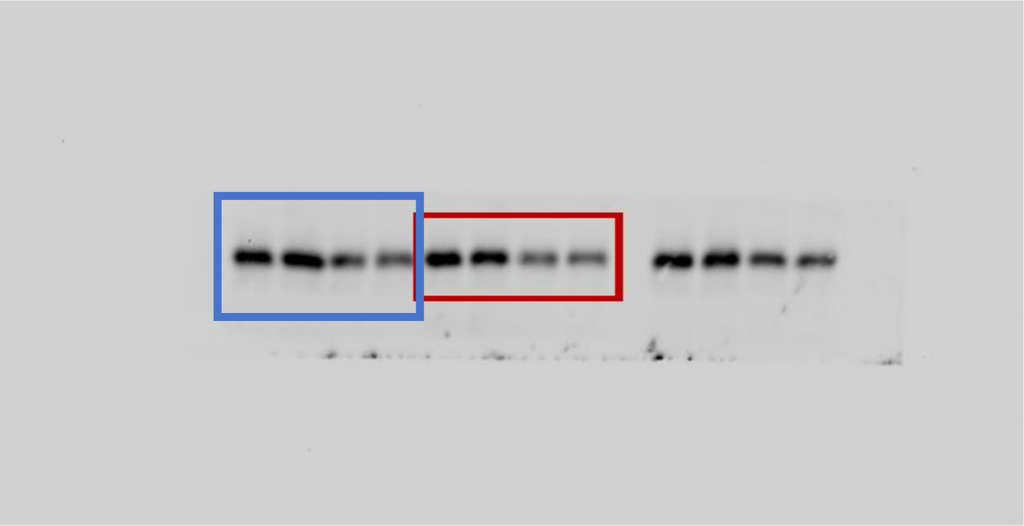

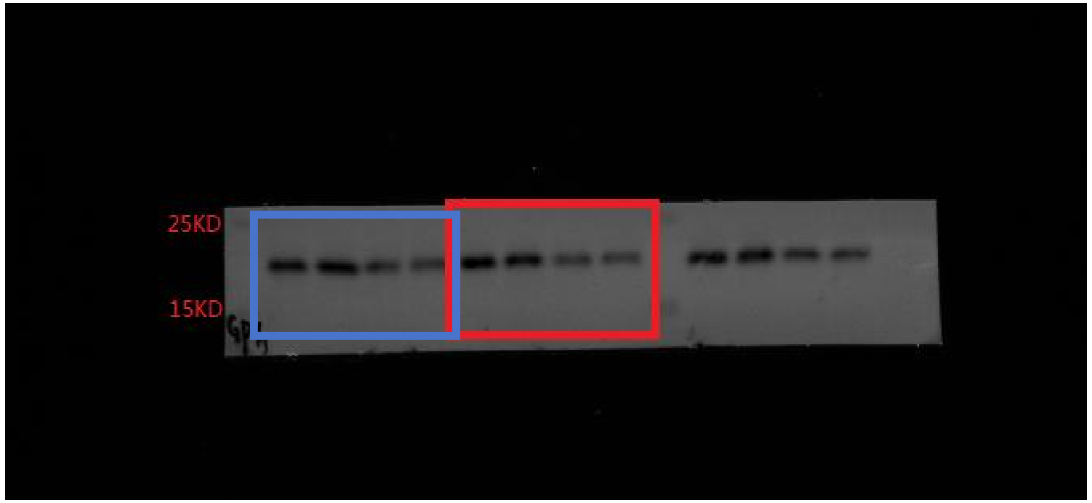

Fig 6 E GPX4 lane 2-5

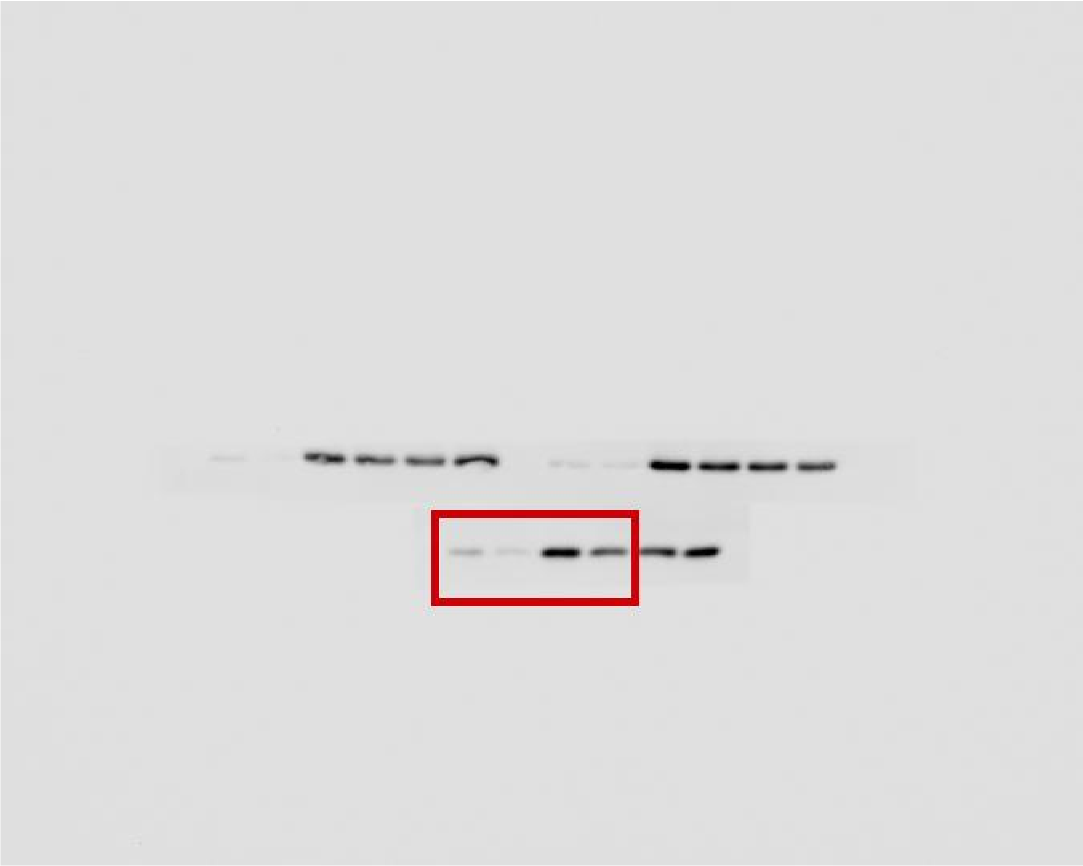

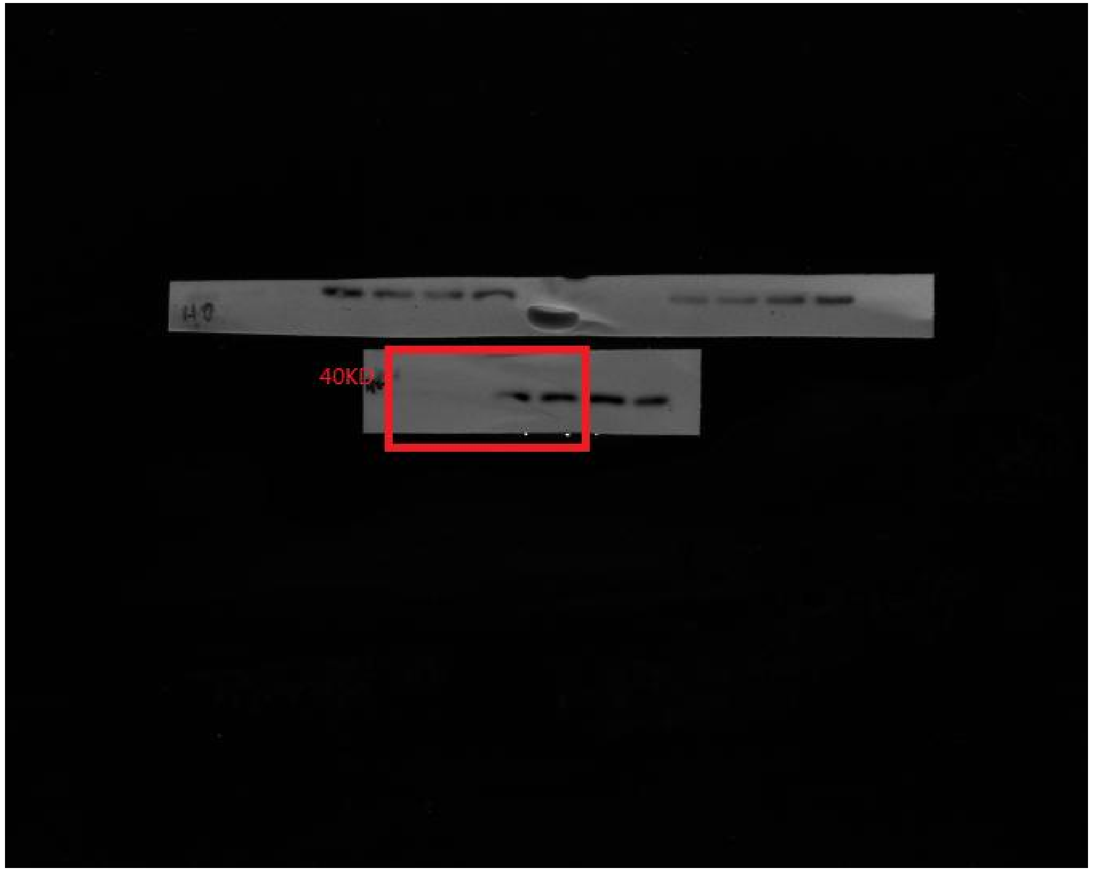

Fig 6 E HO-1 lane 2-5

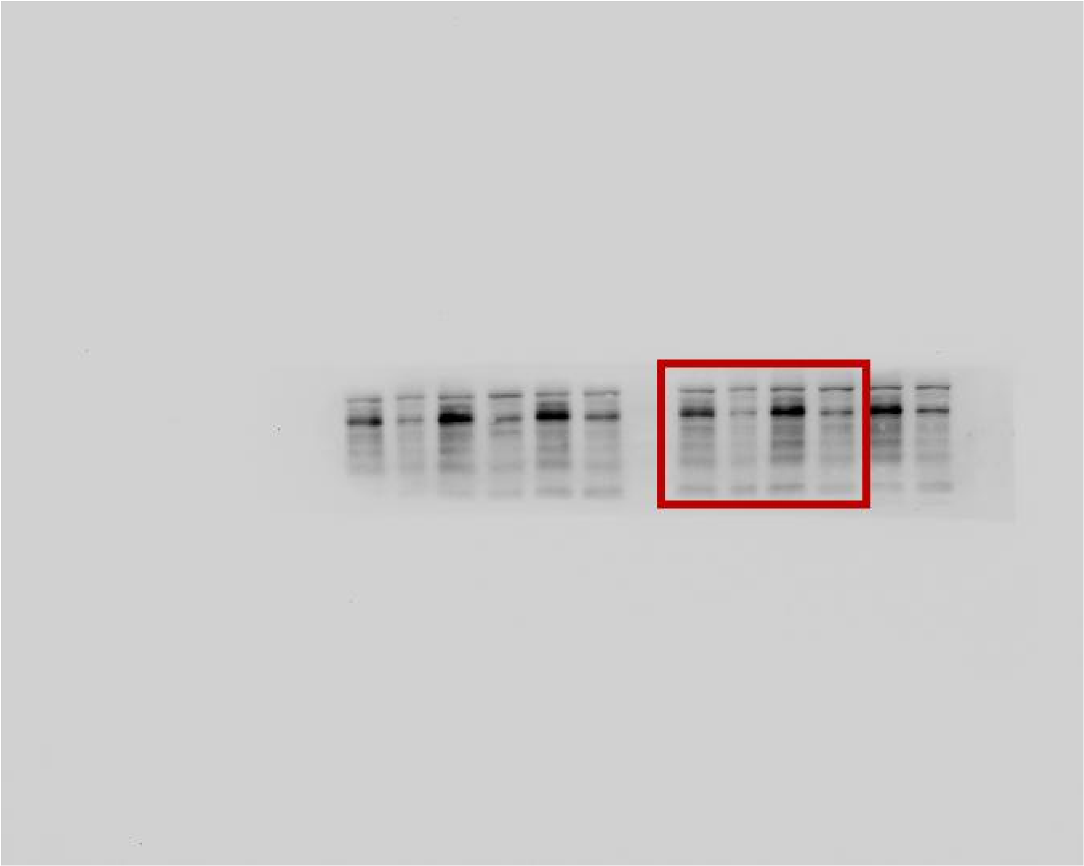

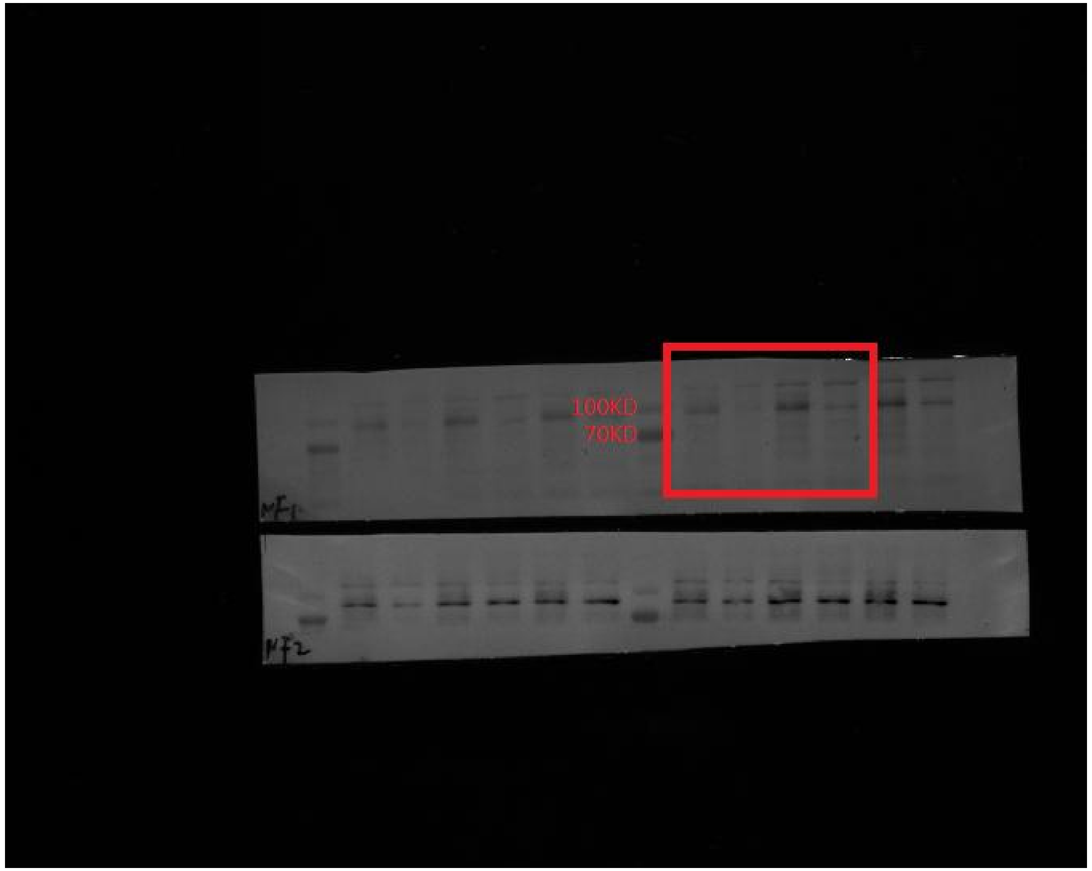

Fig 6 E NRF2 lane 9-12

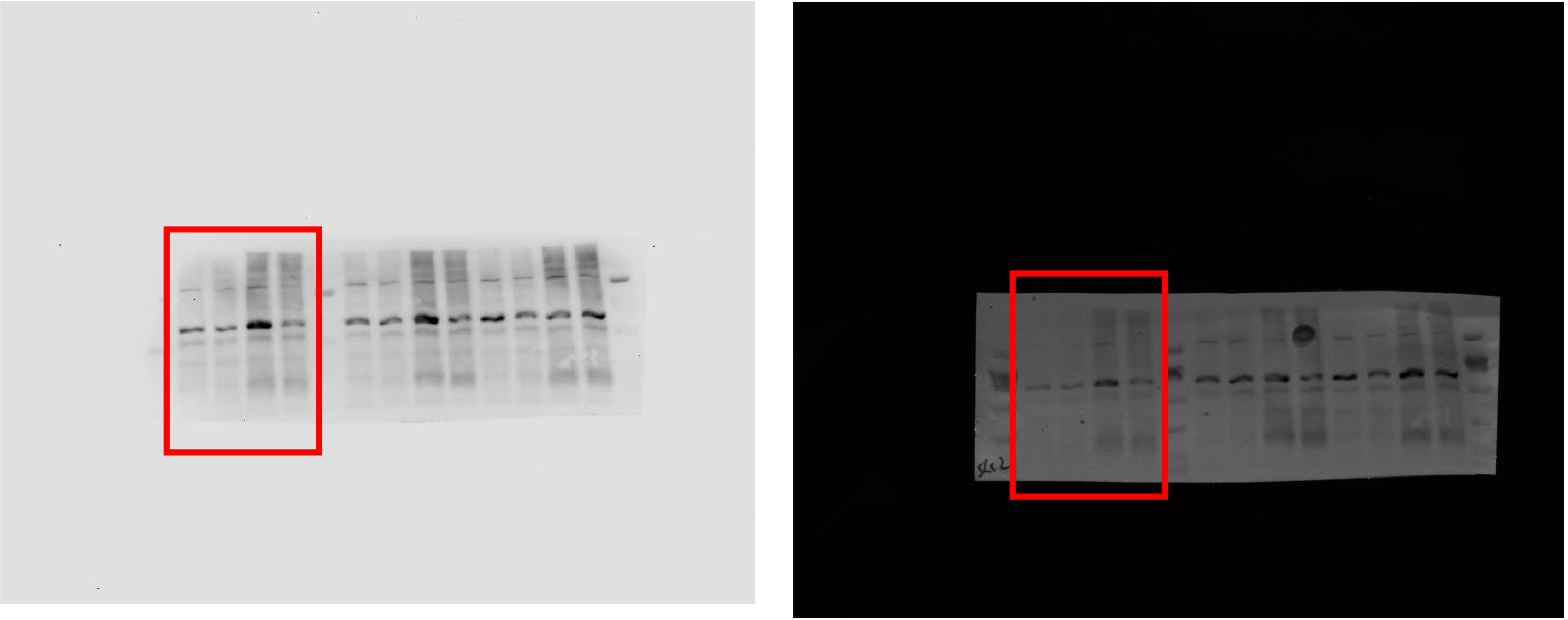

Fig 6 E SLC7A11 lane 2-5

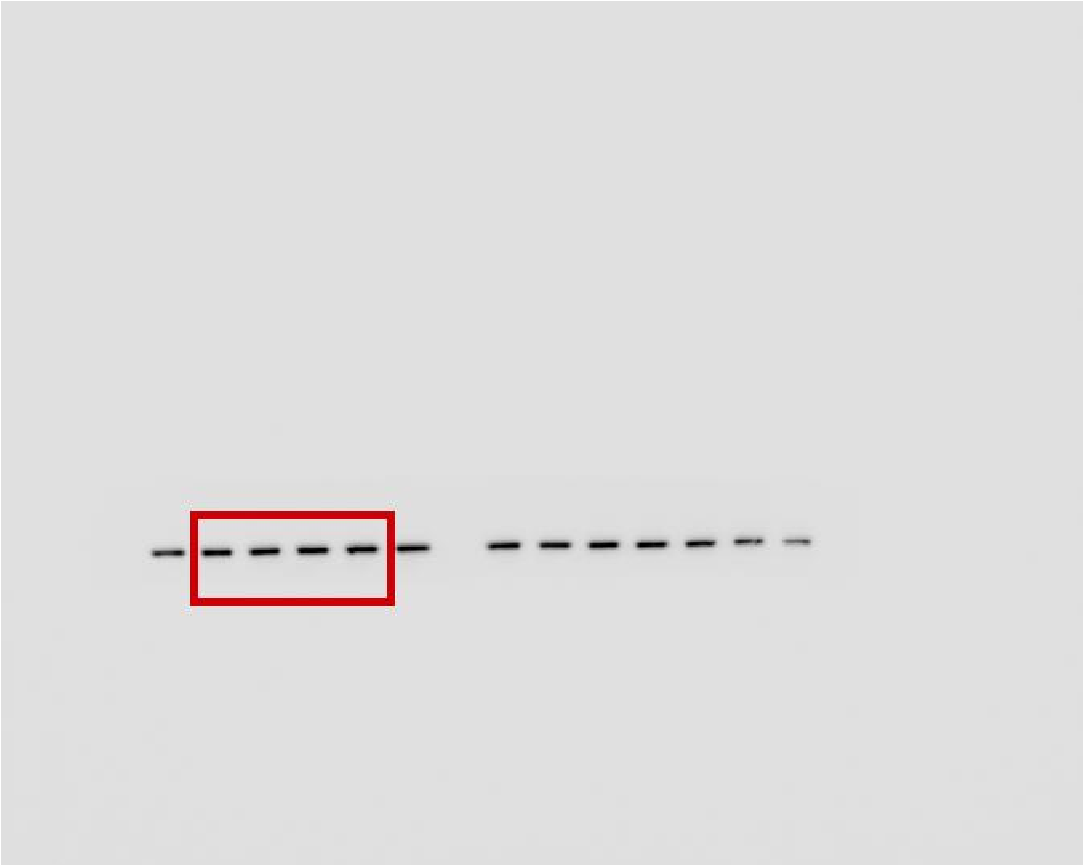

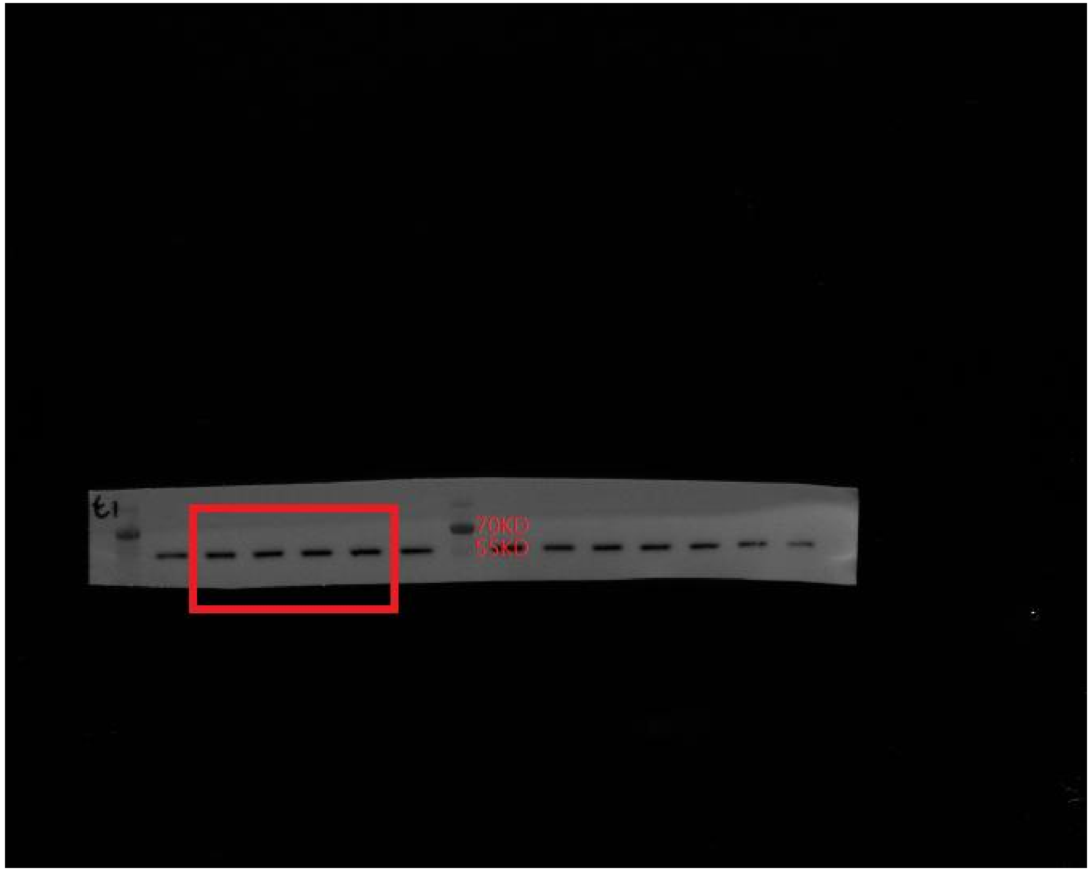

Fig 6 E β-Tubulin lane 3-6

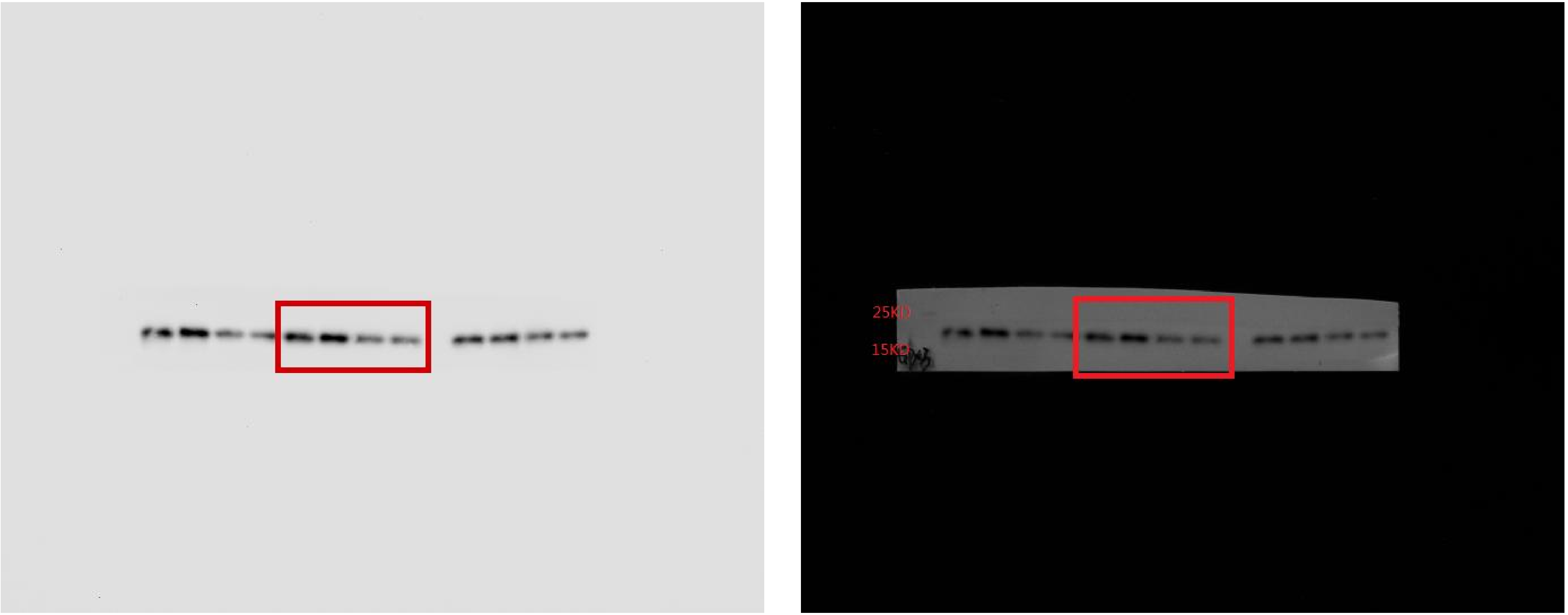

Fig 6 F GPX4 lane 6-9

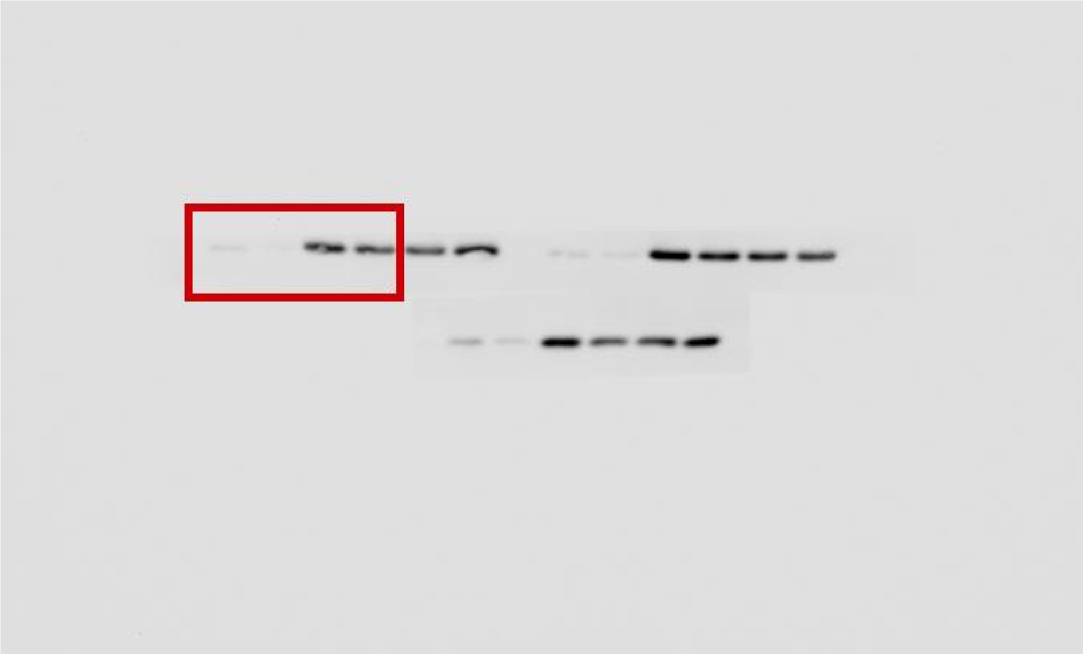

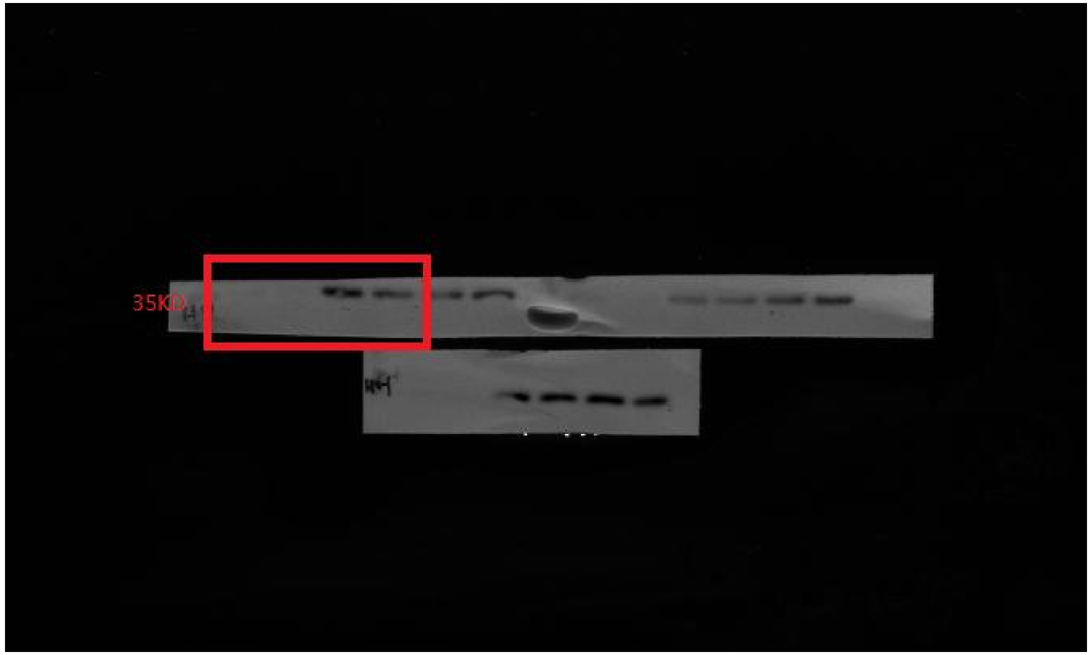

Fig 6 F HO-1 lane 2-5

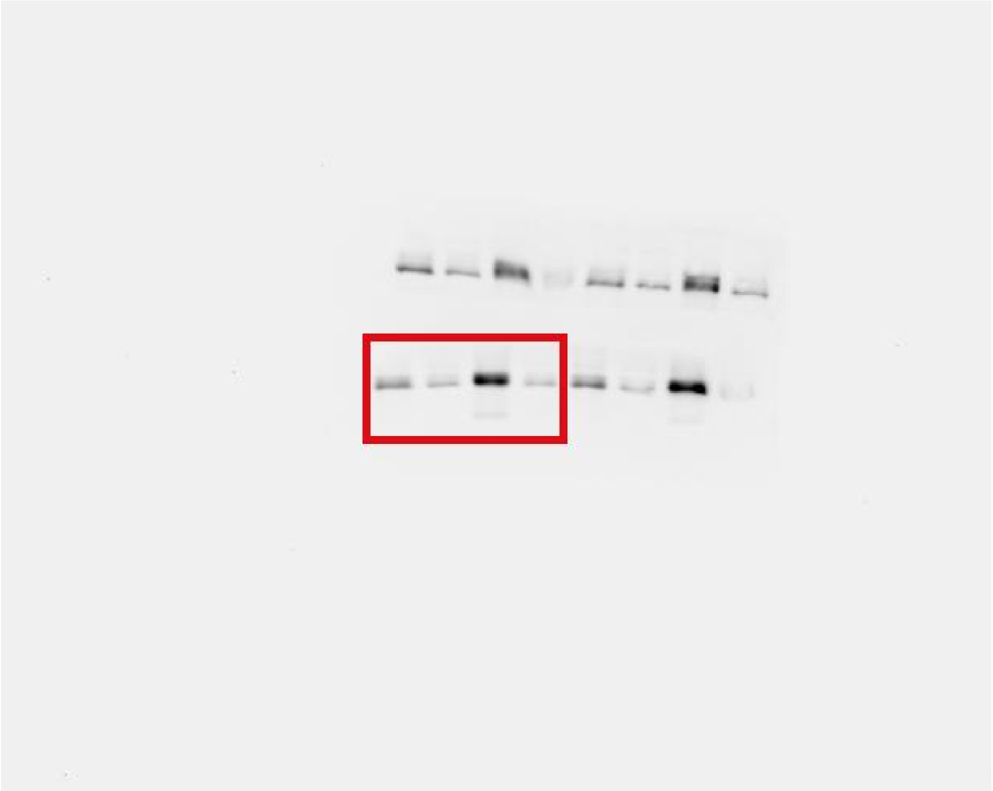

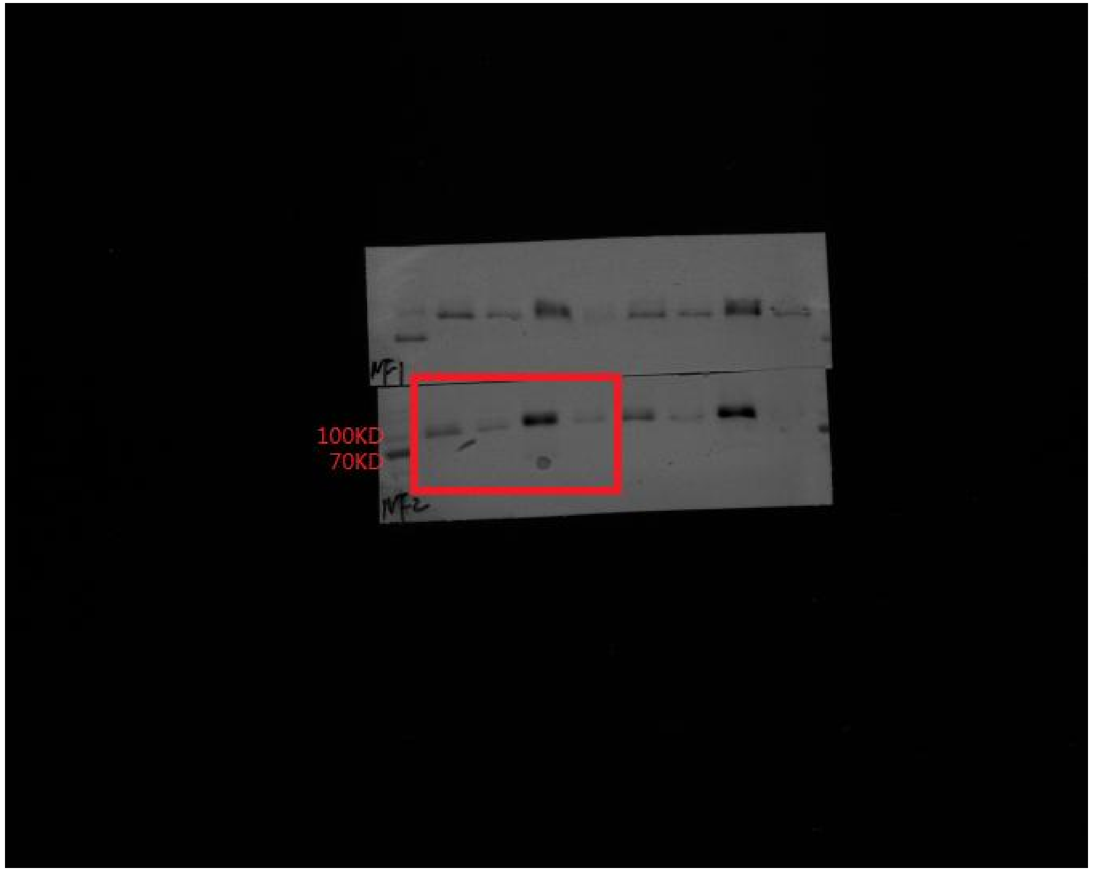

Fig 6 F NRF2 lane 2-5

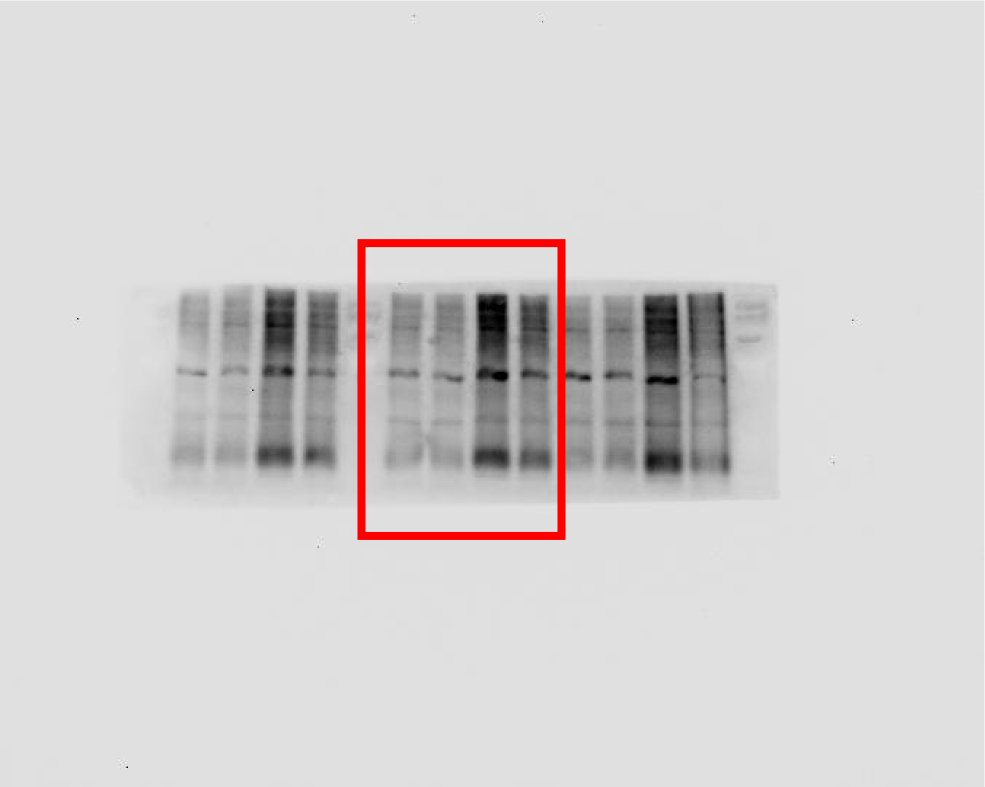
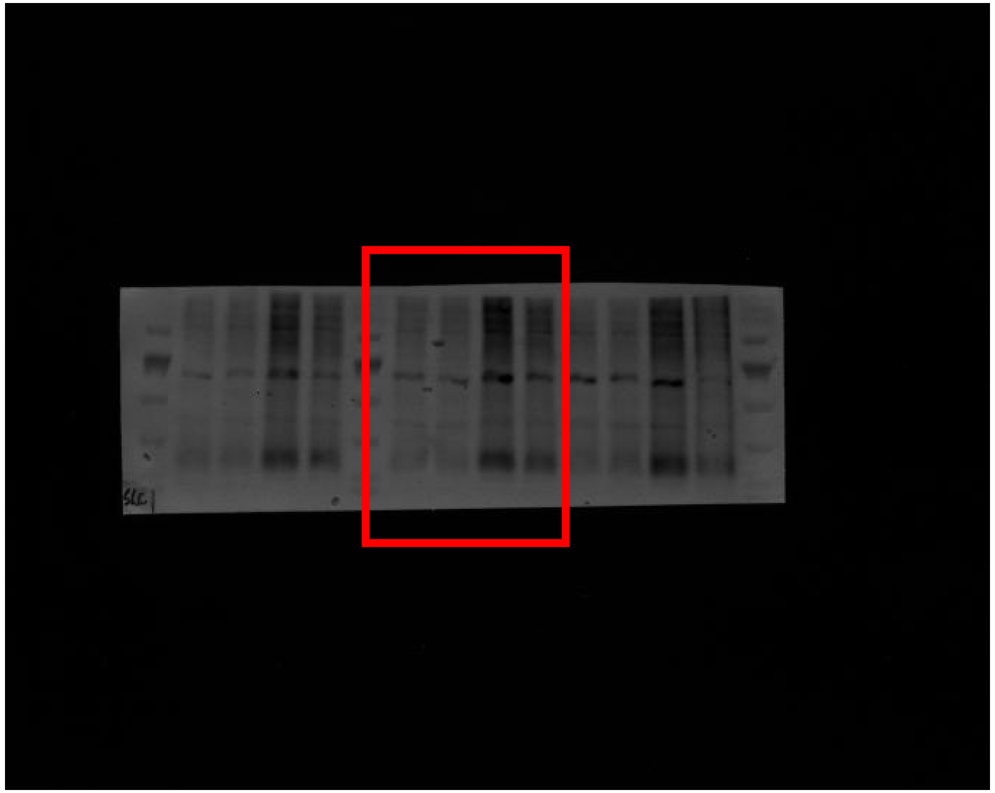

Fig 6 F SLC7A11 lane 7-10

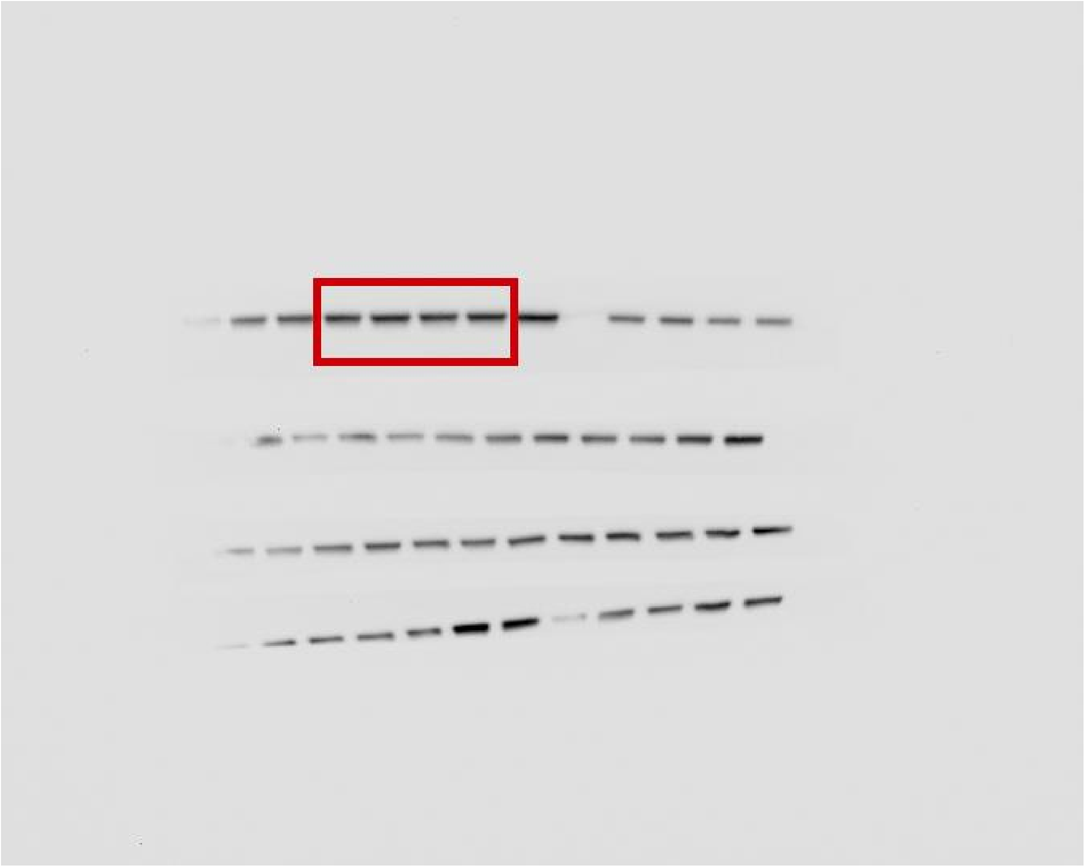

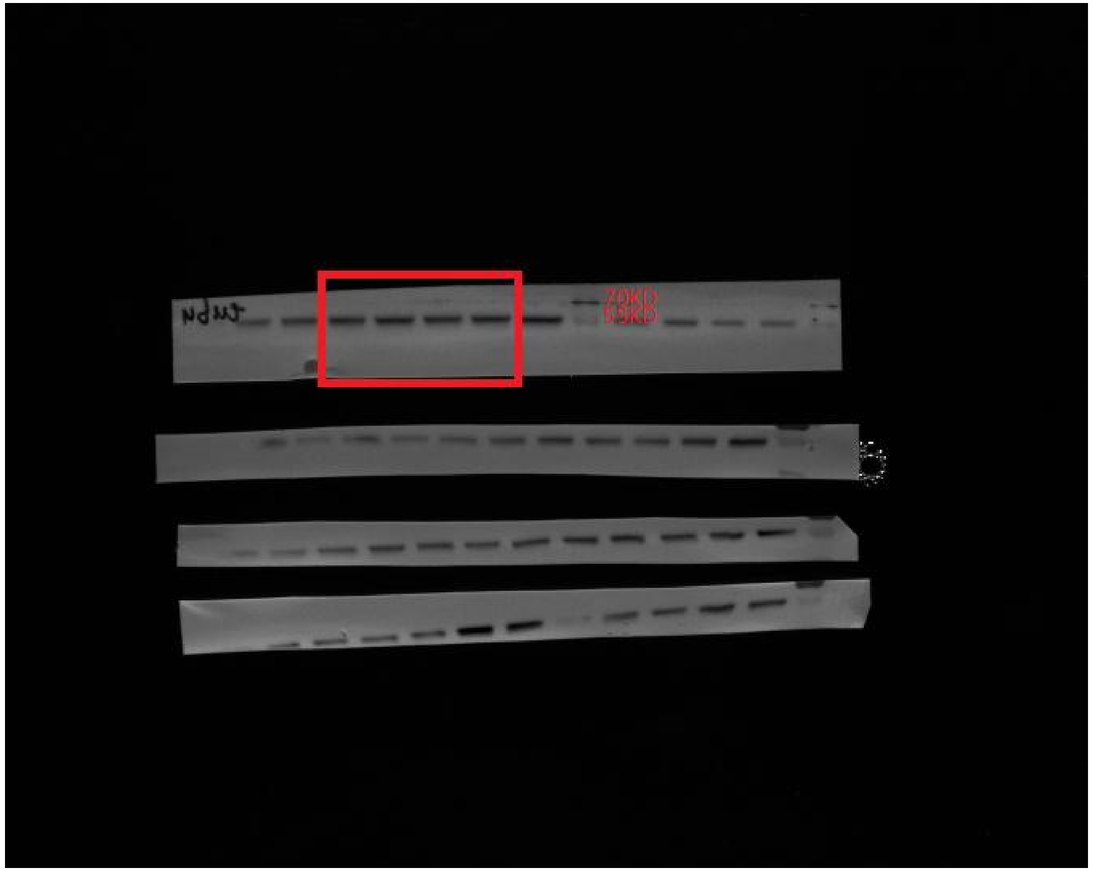

Fig 6 F β-Tubulin lane 4-7
