# Supplementary material for: Silencing NRF2 enhances arsenic trioxide-induced ferroptosis in hepatocellular carcinoma cells
Source: PLoS One. 2025 May 22;20(5):e0322746. doi: 10.1371/journal.pone.0322746 (PMC12097587; doi:10.1371/journal.pone.0322746)
Supplement: Attachment — Submitted filename: response to review.docx [file pone.0322746.s007.docx]

The point-to-point responses are as follow:

Reviewer #1: The Bodipy flow plots (Fig. 2A, 2B, and Fig. 5A, 5C) have been converted into percentage lipid peroxidation (Fig. 2C, 2D, and Fig. 5B, 5D). Please calculate the mean fluorescence intensity for the Bodipy flow plots and show the statistical significance.- this is not addressed satisfactorily

Response：

Thanks. We apologize for any confusion caused by the unclear description in the previous response. The lipid peroxidation sensor C11-BODIPY was used according to the manuscript. Oxidation of the polyunsaturated butadienyl portion of C11-BODIPY resulted in a shift of the fluorescence emission peak from ~590 nm to ~510 nm, proportional to lipid peroxidation generation, and was analyzed using a flow cytometer. C11-BODIPY fluorescence was detected through the FITC channel (510 nm). Mean fluorescence intensity was quantified using FlowJo software, and bar graphs in Figures 2C-D and 5B-D were generated based on the mean fluorescence intensity values from each experimental group. In these graphical representations, the mean fluorescence intensity values directly correspond to the lipid peroxidation levels of individual samples, serving as a quantitative measure of cellular membrane oxidation status across experimental conditions.

Fig. 5G and Fig. 5H – The Nrf2, HO-1, GPX4, and SLC7A11 blots appear identical for both cell lines, HepG2 and Huh7.- the response to this comment is not convincing

Response：

Thanks. We apologize for the misunderstanding in our previous response and for the inaccurate explanation provided. Despite the data originating from different samples of two cell lines, the blots look identical. To address potential misinterpretation, we have replaced the Western blot image with repeated experimental data. The results are presented in Fig. 6 of the revised manuscript.

Additionally, All the original figures of the western blots have been submitted to the journal. The full uncropped original Western blot images for this figure are provided below:
